# Expression of *Idh1*^R132H^ in the Murine Subventricular Zone Stem Cell Niche Recapitulates Features of Early Gliomagenesis

**DOI:** 10.1016/j.ccell.2016.08.017

**Published:** 2016-10-10

**Authors:** Chiara Bardella, Osama Al-Dalahmah, Daniel Krell, Pijus Brazauskas, Khalid Al-Qahtani, Marketa Tomkova, Julie Adam, Sébastien Serres, Helen Lockstone, Luke Freeman-Mills, Inga Pfeffer, Nicola Sibson, Robert Goldin, Benjamin Schuster-Böeckler, Patrick J. Pollard, Tomoyoshi Soga, James S. McCullagh, Christopher J. Schofield, Paul Mulholland, Olaf Ansorge, Skirmantas Kriaucionis, Peter J. Ratcliffe, Francis G. Szele, Ian Tomlinson

**Affiliations:** 1 Molecular & Population Genetics Laboratory, Wellcome Trust Centre for Human Genetics, University of Oxford, Oxford OX3 7BN, UK; 2 Department of Physiology, Anatomy and Genetics, University of Oxford, Oxford OX1 3QX, UK; 3 Nuffield Department of Clinical Medicine, Ludwig Institute for Cancer Research, University of Oxford, Oxford OX3 7DQ, UK; 4 Chemistry Research Laboratory, University of Oxford, Oxford OX1 3TA, UK; 5 Hypoxia Biology Laboratory, Henry Wellcome Building for Molecular Physiology, University of Oxford, Oxford OX3 7BN, UK; 6 Radcliffe Department of Medicine, OCDEM, Churchill Hospital, Oxford OX3 7LJ, UK; 7 Department of Oncology, Cancer Research UK and MRC Oxford Institute for Radiation Oncology, University of Oxford, Oxford OX3 7LE, UK; 8 School of Life Sciences, The Medical School, University of Nottingham, Nottingham NG7 2UH, UK; 9 Bioinformatics, Wellcome Trust Centre for Human Genetics, University of Oxford, Oxford OX3 7BN, UK; 10 Centre for Pathology, St Mary's Hospital, Imperial College, London W2 1NY, UK; 11 Department of Physiology, Institute of Neuroscience and Physiology, Sahlgrenska Academy at University of Gothenburg, 405 30 Gothenburg, Sweden; 12 Institute for Advanced Biosciences, Keio University, 246-2 Mizukami, Kakuganji, Tsuruoka, Yamagata 997-0052, Japan; 13 Department of Oncology, University College London Hospital, London NW1 2BU, UK; 14 Nuffield Department of Clinical Neurosciences, Department of Neuropathology, John Radcliffe Hospital, Headley Way, Oxford OX3 9DU, UK

**Keywords:** glioma, isocitrate dehydrogenase, hydroxyglutarate, α-ketoglutarate, oncometabolite, subventricular zone, stem cell, hydroxylase, DNA (hydroxy)methylation, Wnt

## Abstract

Isocitrate dehydrogenase 1 mutations drive human gliomagenesis, probably through neomorphic enzyme activity that produces D-2-hydroxyglutarate. To model this disease, we conditionally expressed *Idh1*^R132H^ in the subventricular zone (SVZ) of the adult mouse brain. The mice developed hydrocephalus and grossly dilated lateral ventricles, with accumulation of 2-hydroxyglutarate and reduced α-ketoglutarate. Stem and transit amplifying/progenitor cell populations were expanded, and proliferation increased. Cells expressing SVZ markers infiltrated surrounding brain regions. SVZ cells also gave rise to proliferative subventricular nodules. DNA methylation was globally increased, while hydroxymethylation was decreased. Mutant SVZ cells overexpressed Wnt, cell-cycle and stem cell genes, and shared an expression signature with human gliomas. *Idh1*^R132H^ mutation in the major adult neurogenic stem cell niche causes a phenotype resembling gliomagenesis.

## Highlights

• *Idh1*^R132H^ knockin in the mouse brain SVZ recapitulates features of gliomagenesis
• Self-renewal and proliferation of neural stem cells and progenitors increase
• SVZ cells proliferate ectopically, infiltrate the brain parenchyma, and form nodules
• Increases occur in 2HG levels, Wnt and telomere pathway activity, and DNA methylation

## Graphical Abstract

**Figure:**
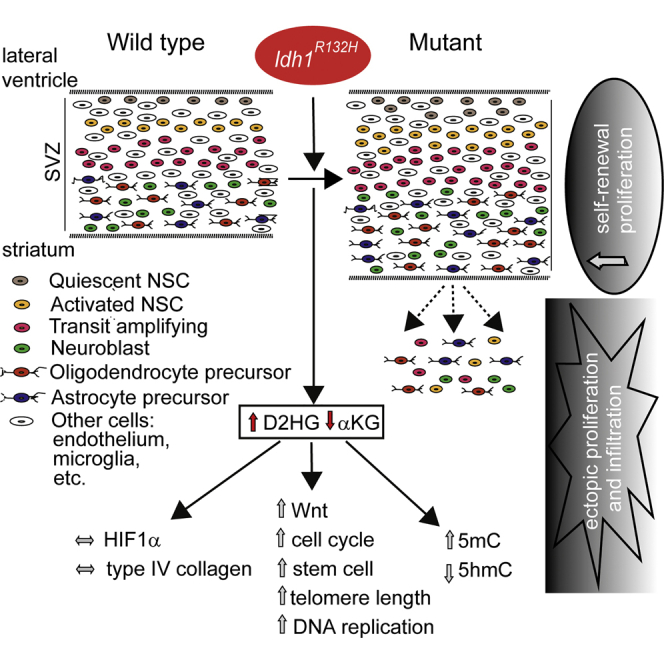

## Teaser

Bardella et al. report that expression of *IDH1*^R132H^ in the subventricular zone of the adult mouse brain causes features of gliomagenesis, including increased numbers of neural stem cells, cellular infiltration into surrounding brain regions, and a gene expression profile overlapping that of human gliomas.

## Significance

**Few curative treatments are available for human brain tumors. *IDH1***^**R132H**^ **is a driver mutation in gliomas and other malignancies, probably causing tumorigenesis through D-2-hydroxyglutarate accumulation, although the downstream mechanisms remain unclear. We found that adult mice expressing *Idh1***^**R132H**^ **in the brain subventricular zone (SVZ) develop features of gliomagenesis, including increased numbers of neural stem cells and their progeny. Other abnormalities included cellular infiltration into surrounding brain regions, reminiscent of tumor invasion. The gene expression profile of the *Idh1***^**R132H**^ **SVZ closely overlaps those of human gliomas. Likely, non-exclusive tumorigenic mechanisms included promotion of a neural stem cell phenotype, Wnt pathway activation, maintenance of telomeres, and DNA hypermethylation. Our *Idh1***^**R132H**^ **mouse provides a system for assessing brain tumor therapies in vivo.**

## Introduction

Gliomas are the most frequent primary brain tumor. They have diverse morphology, genetic status, and response to therapy. Grade I gliomas are characterized by slow growth. Grade II/III gliomas are invasive and progress to higher-grade lesions with poor prognosis. Glioblastoma (grade IV, GBM), the most common and most aggressive glioma, may develop rapidly without evidence of a less malignant precursor lesion (primary), or less often by progression of a lower grade tumor (secondary). Most grade II/III gliomas and secondary GBMs carry mutations in one of isocitrate dehydrogenase genes *IDH1* or *IDH2* (Losman and Kaelin, 2013). *IDH1* or *IDH2* mutations are early events in gliomagenesis, are negatively associated with *PTEN* mutation and *EGFR* amplification, and in the astrocytoma sub-type of glioma, are positively associated with *TP53* mutation (Gupta et al., 2011, Reitman and Yan, 2010). *IDH* driver mutations are also found in acute myeloid leukemia (AML) (Mardis et al., 2009), cholangiocarcinoma (Borger et al., 2012), enchondroma (Amary et al., 2011, Pansuriya et al., 2011), chondrosarcoma (Amary et al., 2011), and occasionally, other tumors. How *IDH* mutations contribute to tumorigenesis is largely unknown.

IDH1 and IDH2 convert isocitrate to α-ketoglutarate (αKG) by oxidative decarboxylation, also generating NADPH. These enzymes share sequence and functional homology. IDH1 is found in the cytoplasm and peroxisome where it acts in lipid and glucose metabolism and protects against reactive oxygen species (ROS). IDH2 localizes to the mitochondria, where it regulates the tricarboxylic acid (TCA) cycle (Reitman and Yan, 2010).

In gliomas, *IDH1*^R132H^ is the most common mutation (Parsons et al., 2008). Initial in vitro studies found that mutant IDH1 had decreased affinity for isocitrate (Zhao et al., 2009) and led to reduced αKG and NADPH levels (Yan et al., 2009, Zhao et al., 2009). Thus, *IDH1* mutations were initially thought to cause tumors by loss of function. Subsequent studies showed mutant IDH1 gained an enzymatic function that converts αKG to D-2-hydroxyglutarate (D2HG) (Dang et al., 2009). D2HG was postulated to be an “oncometabolite” based on reports of brain tumors in patients with congenital *L2HGDH* (L-2-hydroxyglutarate dehydrogenase) deficiency, in whom L2HG accumulates because it cannot be metabolized to αKG (Aghili et al., 2009, Moroni et al., 2004, Patay et al., 2012, Patay et al., 2015). Further in vitro data showed that provision of D2HG in *IDH1/2-*wild-type hematopoietic cells had similar leukemogenic activity to *IDH1* mutations (Losman and Kaelin, 2013). The tumorigenic effects of D2HG may derive from modulating αKG-dependent enzymes such as JmjC domain histone demethylases (JHDMs), TET 5-methylcytosine hydroxylases that convert 5′-methylcytosine (5mC) to 5′-hydroxymethylcytosine (5hmC) (Xu et al., 2011), and prolyl hydroxylases (PHDs) that have targets such as HIF1α and collagen (Borger et al., 2012, Chowdhury et al., 2011, Duncan et al., 2012, Figueroa et al., 2010, Hirata et al., 2015, Lu et al., 2012, Sasaki et al., 2012b, Tarhonskaya et al., 2014, Xu et al., 2011). Evidence for these possibilities varies: for example, HIF pathway changes reported in *IDH1* mutants vary from activation through no change to inactivation.

Several mice carrying pathogenic *Idh1* or *Idh2* mutations have been analyzed. Sasaki et al. (2012b) conditionally expressed *Idh1*^R132H^ in hematopoietic lineages, leading to raised progenitor cell numbers and extra-medullary hematopoiesis. Mutant cells showed increased histone and DNA methylation, consistent with findings in human AML (Figueroa et al., 2010). Knockin of *Idh1*^R132H^ in brain progenitors from E10.5 using nestin-Cre caused neonatal death due to brain hemorrhages and high levels of apoptosis (Sasaki et al., 2012a). No stem cell abnormalities were observed in these mice, but HIF1α was stabilized and collagen maturation was aberrant. Histone lysine methylation was unchanged, but in very early embryos, 5hmC levels were greatly reduced. In a parallel experiment using GFAP-Cre, which acts from E14.5 in neural stem cells (NSCs), 60% of mice suffered brain hemorrhages, and few survived to adulthood. Akbay et al. (2014) ubiquitously expressed mutant *Idh2* (R140Q or R172K) in 5-week-old mice, resulting in cardiomyopathy and white matter abnormalities throughout the CNS. None of these *Idh-*mutant mice developed phenotypes clearly related to brain tumorigenesis.

There is evidence that many human gliomas arise from the subventricular zone (SVZ, also known as the V-SVZ), the largest NSC niche in the adult mammalian brain (Ihrie and Alvarez-Buylla, 2011). In the canonical model of normal SVZ lineage progression, quiescent NSCs become activated and generate rapidly dividing transit amplifying progenitor (TAP) cells. These, in turn give rise to lineage committed neuroblasts or glioblasts that differentiate and in mice migrate to the olfactory bulb. How the stem cell niche generates brain tumors is not well understood, but evidence supports a link between SVZ NSCs and gliomas. First, Wnt, EGF, SHH, and PDGF signaling are necessary for SVZ stem cell self-renewal and proliferation, and are also implicated in tumorigenesis (Jackson and Alvarez-Buylla, 2008). Second, nestin and other SVZ stem cell markers are highly expressed in GBMs (Haskins et al., 2013, Singh et al., 2004). Finally, mutations or aberrant expression of genes such as *Kras, Pdgfb, Trp53, Ptch1*, and *Pten* in the SVZ of mice can elicit tumors (Fomchenko and Holland, 2006, Holland, 2001, Huse and Holland, 2009). Multiple genetic perturbations are often necessary for progression from hyperproliferation to full-blown SVZ tumors.

We wondered whether the existing *Idh1*^R132H^ mice had not developed features of brain tumors because the mutant protein was deleterious in embryos or young animals. Consistent with this notion, we noted that germline *IDH* mutations had not been reported in patients with gliomas or AML, and that the few patients with constitutional *IDH* mutations were mosaics (Amary et al., 2011). We therefore investigated the consequence of expressing *Idh1* R132H specifically in adult NSCs and progenitor cells in mice.

## Results

### Knockin of *Idh1*^*fl*(R132H*)/+*^ in the Adult Mouse SVZ Stem Cell Niche

**Figure 1:**
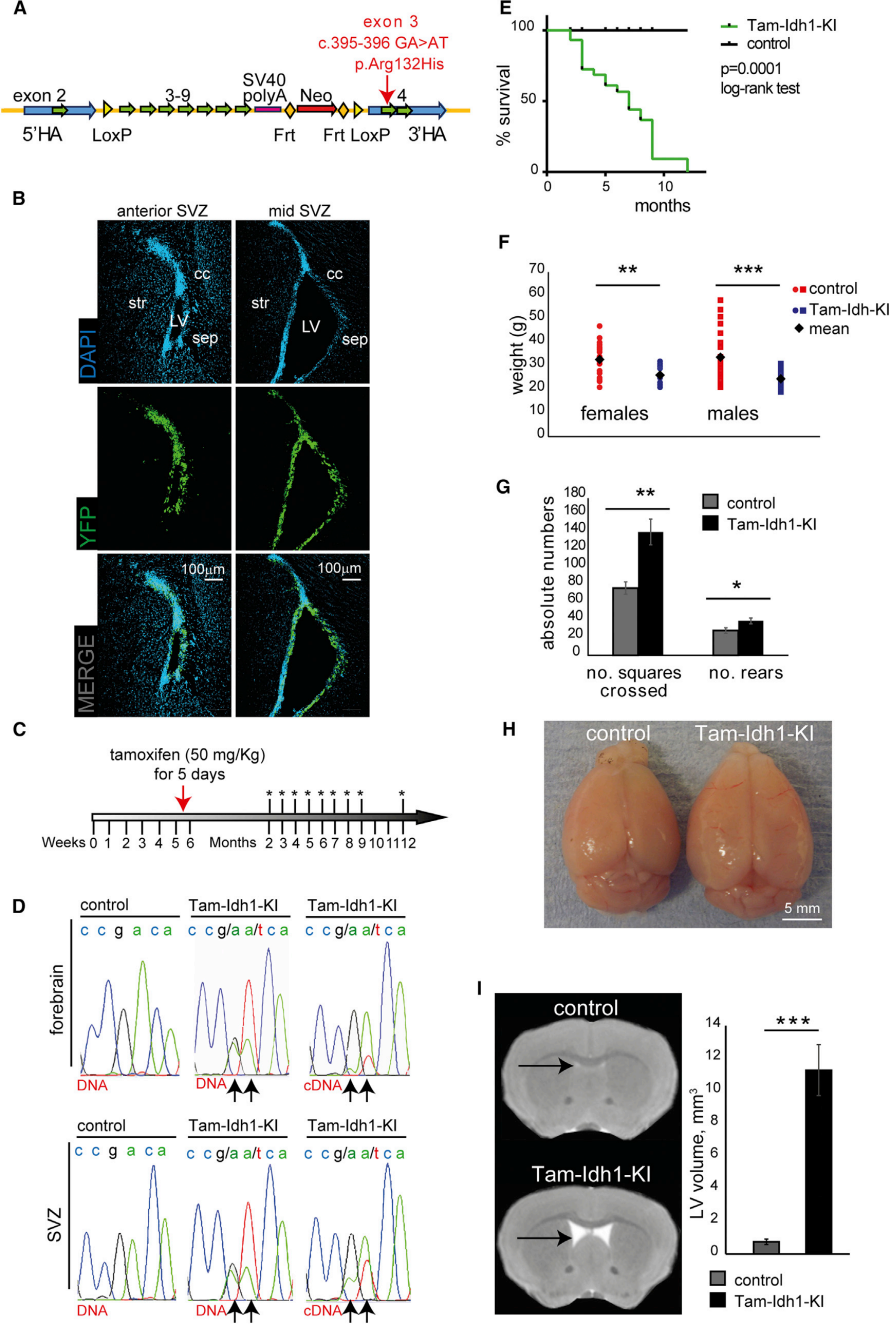
*Idh1*^R132H^ Expression in the SVZ and Its Effects on Weight, Behavior, and Lateral Ventricle Size in Adult Mice (A) The construct used to generate Tam-Idh1-KI mice is shown. After homologous recombination into the endogenous *Idh1* locus, expression of Cre causes deletion of (1) a mini-gene containing *Idh1* wild-type exons 3–9; (2) termination codon and SV40 polyA signal; and (3) Neo^R^ cassette. *Idh1*^R132H^ is then expressed from the native promoter. The following features are shown: *loxP* and Frt sites; 5′ and 3′ homology arms (HAs); wild-type mini-gene (exons 3–9) and SV40 polyA signal; neomycin resistance cassette (Neo^R^); location of the R132H mutation. (B) The panels show expression of the YFP reporter in the anterior SVZ (left) and mid-SVZ (right) of *Nes-Cre*^*ER(T2)*^*;R26R-EYFP* mice 29 weeks after tamoxifen induction. cc, corpus callosum; str, striatum; sep, septum; LV, lateral ventricle. (C) Tamoxifen dosage schedule is shown. Asterisks indicate time points of brain collections. (D) Sequencing chromatogram shows genomic DNA or cDNA of a region around *Idh1* codon 132 from forebrain or SVZ of control or Tam-Idh1-KI mice as indicated. Arrows indicate nucleotides altered in *Idh1*^R132H^ (c. 395–396 CGA > CAT). (E) Kaplan-Meier plots show the survival of Tam-Idh1-KI (n = 28) and control (n = 32) mice from 18 litters. (F) Body weights of paired Tam-Idh1-KI and control mice are shown. (G) Open field test measures of locomotion (no. of squares crossed) and activity (no. of rears) are shown. (H) Whole-brain dissections show frontal and parietal lobe morphology in Tam-Idh1-KI and control animals. (I) MRI illustrates LV volumes (arrows) in Tam-Idh1-KI mice and controls. Sections were taken at comparable locations. Estimated LV volumes of three Tam-Idh1-KI mice and three controls are shown in the chart. All data are presented as mean ± SD (^∗^p < 0.05, ^∗∗^p < 0.01, ^∗∗∗^p < 0.005). See also Figure S1.

To generate *Idh1*^*fl(*R132H*)/+*^ knockin mice, we designed a replacement targeting construct to conditionally express the *Idh1*^R132H^ mutation under the control of the endogenous promoter (Figure 1A) following recombination at *loxP* sites. Initially we targeted the mutation specifically to brain stem/progenitor cells by crossing *Idh1*^*fl(*R132H*)/+*^ animals with nestin-Cre mice, thus inducing efficient recombination throughout the CNS from E10.5 (Tronche et al., 1999). As expected, these Idh1-KI mice died perinatally and exhibited brain hemorrhages (Sasaki et al., 2012a) (Figure S1A). We then crossed *Idh1*^*fl(*R132H*)/+*^ animals with mice carrying a tamoxifen-inducible nestin-Cre^ER(T2)^, which in adult mice targets Cre to the SVZ and the other major neurogenic niche, the subgranular zone (SGZ) of the hippocampal dentate gyrus (Lagace et al., 2007). We confirmed this using Rosa26-YFP reporter mice (Figure 1B). Tamoxifen was given to the *Nes-Cre*^*ER(T2)*^*; Idh1*^*fl(*R132H*)/+*^ mice at 5–6 weeks of age for 5 consecutive days (Tam-Idh1-KI mice) (Figure 1C). We showed that R132H knockin had occurred by sequencing DNA and mRNA from forebrain and microdissected SVZ (Figure 1D).

Throughout the studies described below, we compared Tam-Idh1-KI mice with *Idh1*-wild-type controls (see Experimental Procedures), all of which were given the same regimen of tamoxifen. The Tam-Idh1-KI animals began to suffer morbidity and mortality 4–6 weeks after tamoxifen administration, necessitating culling of about one-third of them at this stage and all by 12 months of age (Figure 1E). Weight loss was usually the first apparent abnormality (Figure 1F), often accompanied by hunched posture and hyperactivity (Figure 1G). Control mice showed none of these problems. Postmortem analysis was performed on 45 Tam-Idh1-KI mice aged 2–12 months. The brains of 19 (42%) animals were grossly uniformly enlarged and less firm (Figure 1H); controls did not show these changes. Craniofacial morphology and skull size appeared normal in Tam-Idh1-KIs, and no brain hemorrhages or gross abnormalities outside the brain were found. Closer visual examination, confirmed by magnetic resonance images, showed enlarged lateral ventricles (LVs), and possibly enlarged third ventricles, in all Tam-Idh1-KI animals (Figures 1I, S1B, and S1C). These appearances were suggestive of obstructive (non-communicating) hydrocephalus.

### *Idh1*^R132H^ Expands the SVZ

**Figure 2:**
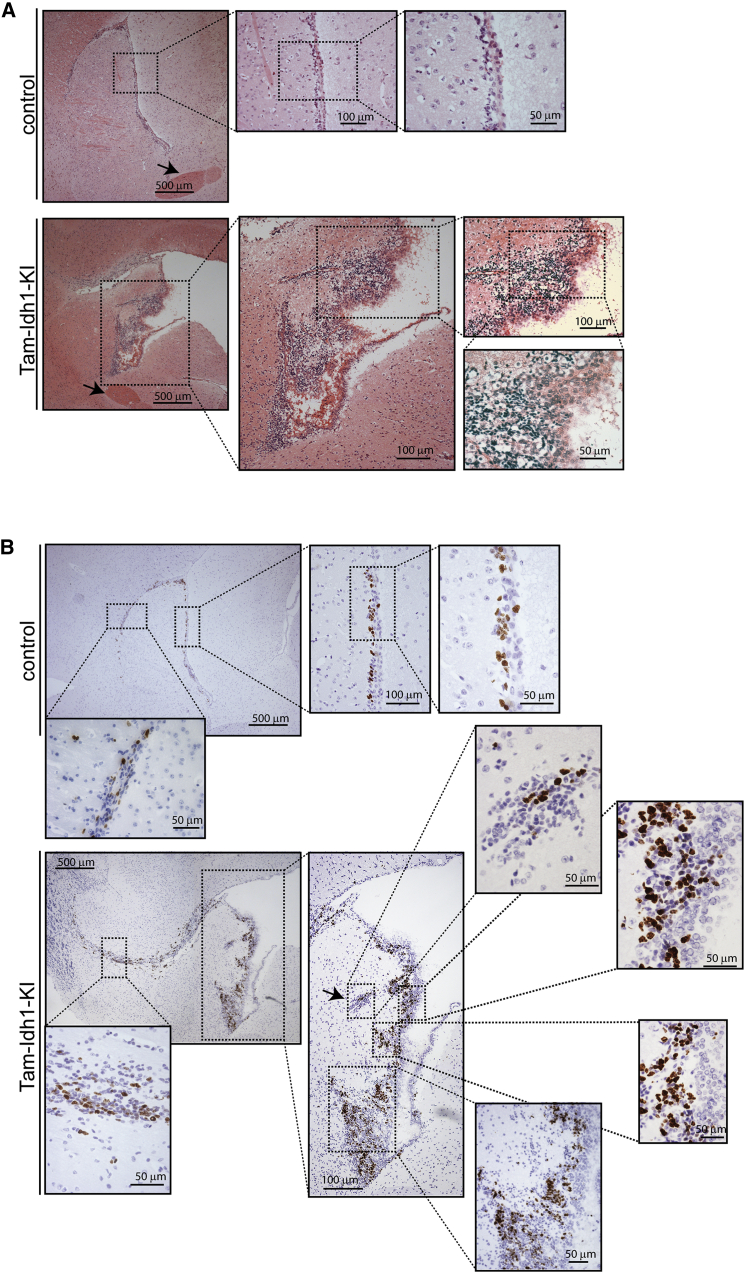
Histology and Proliferation of SVZ in Tam-Idh1-KI and Control Mice (A) H&E staining of sagittal sections from control and Tam-Idh1-KI mice is shown. The anterior commissure is indicated by arrows. (B) Ki67 immunohistochemistry is shown in sagittal sections at comparable medio-lateral positions from Tam-Idh1-KI and control mice. Ki67^+^ cell clusters ectopic to the SVZ are arrowed. Areas in the insets are magnified.

Histological analysis of H&E-stained sections of brain in the sagittal and coronal planes demonstrated lateral ventricular dilation in all Tam-Idh1-KI animals, including both the youngest mice examined (2 months old) and mice with no abnormal physical signs or gross brain enlargement (details not shown). There was also enlargement of the SVZ, with irregular lateral borders, and an expansion of the rostral migratory stream (RMS) (Figure 2A). Furthermore, Ki67 immunohistochemistry (IHC) showed increased numbers of proliferative cells in the SVZ of Tam-Idh1-KI mice (Figure 2B), and these cells spread into the adjacent striatum. The frequency of apoptotic cells was very low in all mice, with no significant difference observed between the two groups (data not shown). There was no morphological evidence of other brain damage or degeneration in Tam-Idh1-KI mice (Figures S1D and S1E).

### *Idh1*^R132H^ Increases the Numbers of Stem and Progenitor Cells in the SVZ

We next examined Tam-Idh1-KI mice using immunofluorescence (IF) for cellular phenotypes related to tumorigenesis, including proliferation, differentiation, and migration. Owing to the abnormalities found in H&E sections, we focused on the SVZ. IF-based quantitation confirmed that, compared with controls, Tam-Idh1-KI mice showed a larger SVZ volume (1.15-fold, p = 0.041), similar cell density (p = 0.20), and a larger number of cells (p = 0.047).

**Figure 3:**
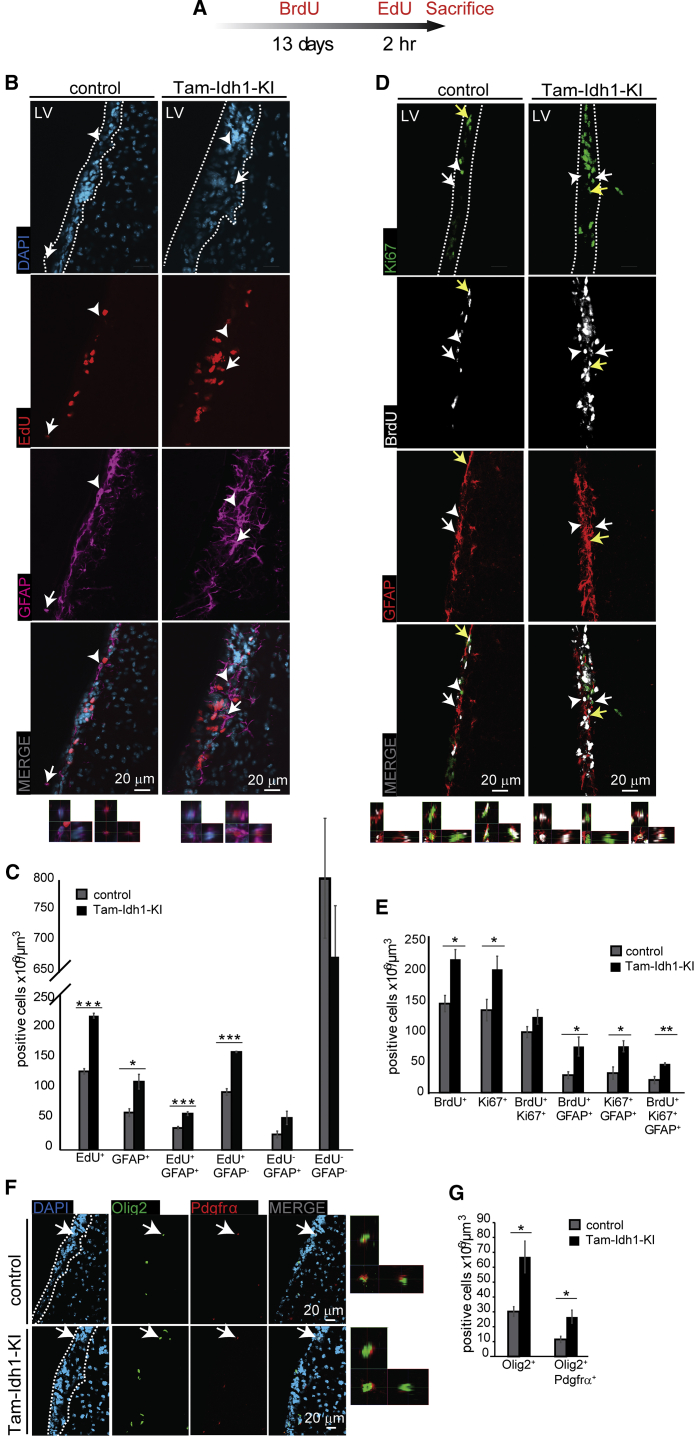
The Effect of *Idh1*^R132H^ on Label-Retaining Cells, Proliferating Cells, and Oligodendrocytes in the SVZ (A) The time course of BrdU and EdU injections is shown. (B) For the analysis of rapidly cycling cells, EdU and GFAP expression in the SVZ was determined. Representative images showing EdU and GFAP expression were derived from coronal sections from four Tam-Idh1-KI and four control mice. The left-hand image at the bottom of each panel is a magnified orthogonal 3D view of the GFAP^+^EdU^−^ cell marked by an arrowhead; and the adjacent right-hand image is the equivalent view of the GFAP^+^EdU^+^ cell marked by an arrow. Dashed lines outline the SVZ. (C) Z stack quantifications of each cellular population from (B) are shown in the chart as “density”, which is a measure of the proportion of each cell type. Total EdU^+^ and GFAP^+^ cells are shown, followed by a breakdown of these into the component categories and an EdU^−^GFAP^-^ group. (D) For analysis of label-retaining cells, BrdU, Ki67, and GFAP expression was assessed. The left-hand image at the bottom of each panel is a magnified orthogonal 3D view of the GFAP^+^BrdU^+^ cell marked by a white arrow; the adjacent middle image is the equivalent view of the GFAP^+^Ki67^+^ cell marked by a white arrowhead; and the adjacent right-hand image is the equivalent view of the GFAP^+^BrdU^+^Ki67^+^ cell marked by a yellow arrow. Other annotation is as per (B). (E) The chart shows quantification of each directly counted, marker-positive cell population from (D). Note that BrdU^+^ cells may have any Ki67 and GFAP status and BrdU^+^Ki67^+^ cells include both GFAP^+^ and GFAP^−^ cells. (F) Olig2^+^ (pan-oligodendrocyte) and Pdgfrα^+^ (progenitors) expression are shown. The image at the extreme right of each panel is a magnified orthogonal 3D view of the Olig2^+^Pdgfrα^+^ cell indicated by a white arrow. Other annotation is as per (B). (G) The chart shows quantification of cell populations from (F). Note that Olig2^+^ includes cells with any Pdgfrα status. All data are presented as mean ± SD (^∗^p < 0.05, ^∗∗^p < 0.01, ^∗∗∗^p < 0.005). See also Figure S2.

To better evaluate the proliferative dynamics of the SVZ and to obtain information regarding which cell populations (Figure S2A) were expanded in Tam-Idh1-KI animals, mutant and control mice were injected with BrdU in three consecutive daily doses, followed 13 days later by a pulse of EdU 2 hr before sacrifice (Figure 3A). The BrdU label-retaining cells in the SVZ correspond to relatively quiescent NSCs, but not their progeny, TAPs, which divide frequently. The EdU label is found in cells cycling at the time of injection. We carried out double IF of EdU and GFAP (Figures 3B and 3C) and triple IF of Ki67, BrdU, and GFAP (Figures 3D and 3E), and quantified SVZ cells with confocal microscopy.

We analyzed the number of cells per unit volume to control for the increased size of the mutant SVZ. The proportions, and hence total numbers, of EdU^+^ (rapidly proliferating or re-entering cell cycle), GFAP^+^ (mainly NSCs and niche astrocytes), and EdU^+^GFAP^+^ cells (proliferating NSC or niche astrocytes) were increased in Tam-Idh1-KIs (Figure 3C). The proportions and numbers of Ki67^+^ (cycling) and BrdU^+^ (quiescent or slowly cycling) cells also increased in the mutant SVZ (Figure 3E). Both Ki67^+^GFAP^+^ and BrdU^+^GFAP^+^ proportions were higher in the mutant SVZ, suggesting that the number of label-retaining, as well as actively dividing, astrocytic stem cells was increased. This was confirmed by the significantly higher proportion and number of BrdU^+^Ki67^+^GFAP^+^ cells in the mutants, suggesting that more NSCs became activated and re-entered the cell cycle. A more detailed analysis of the Ki67, BrdU, and GFAP triple IF found an overall highly significant difference in the proportions of cell types between Tam-Idh1-KIs and controls (χ^2^_5_ = 43, p < 0.001). The increased cell populations included quiescent and activated NSCs and, to a smaller degree, TAPs (Figure S2B).

We also assessed the oligodendrocyte progenitor marker Pdgfrα and Olig2, a marker of the entire oligodendroglial lineage that is universally expressed in diffuse gliomas (Ligon et al., 2004, Suzuki et al., 2014) (Figures 3F and 3G). The proportions and numbers of Pdgfrα^+^Olig2^+^ and total Olig2^+^ cells were both increased in Tam-Idh1-KI mice compared with controls. A similar increase was found for the neuroblast marker Dcx (details not shown).

We assessed Ki67, GFAP and Olig2 expression in younger mice that had received tamoxifen at 5–6 weeks of age and were analyzed 3–8 weeks later. Compared with controls, the younger Tam-Idh1-KI mice showed similar GFAP and Olig2 changes to those observed previously in older animals, although the Ki67 increase was smaller (details not shown). The younger mice also had an expanded SVZ, a diffuse RMS and ectopic cells. These data suggest that the Tam-Idh1-KI phenotype can develop quite rapidly but may also progress over time.

In summary, *Idh1*^R132H^ increased the proportions and numbers of quiescent NSCs and their proliferative progeny (activated stem cells, progenitor cells, and partly differentiated cells) in both young and older mice, thereby enlarging the SVZ (Figure S2C). A parsimonious explanation for these findings is a primary effect of the mutation on quiescent NSCs, although additional effects in their progeny cannot be excluded.

### *Idh1*^R132H^ Induces Infiltration into the Parenchyma

**Figure 4:**
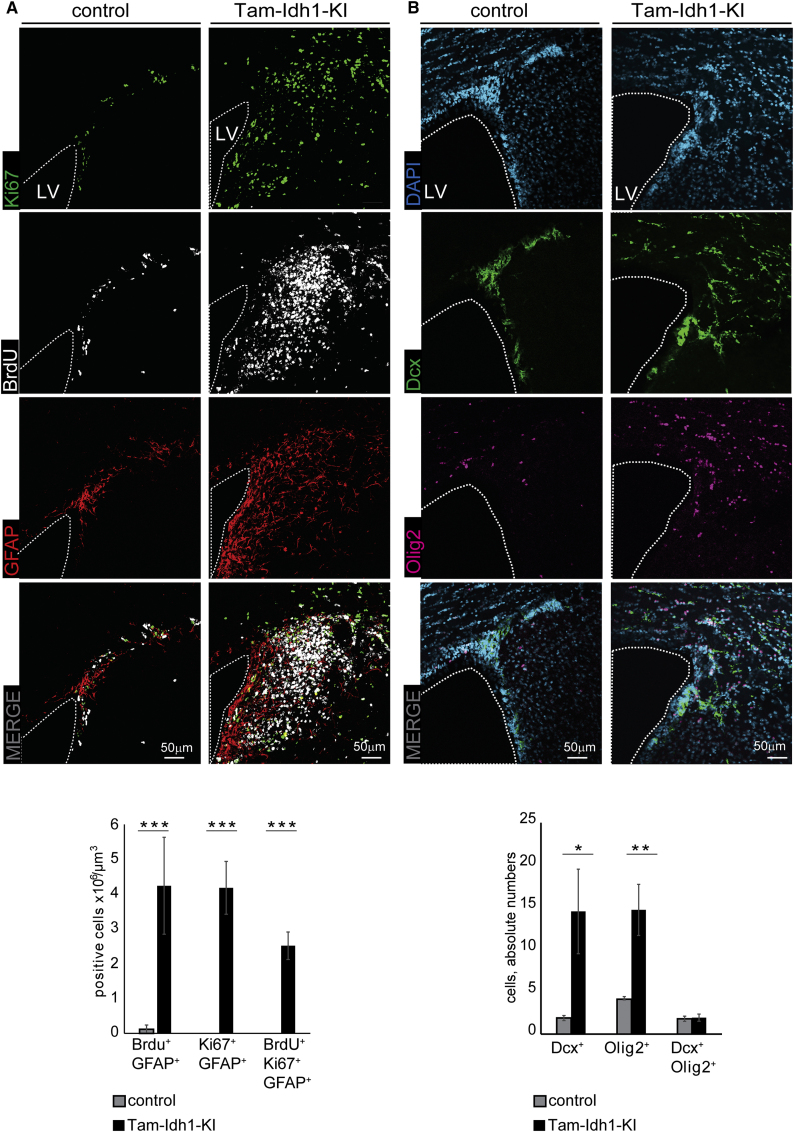
The Effect of *Idh1*^R132H^ on Tissues Surrounding the Dorsolateral Corner of the SVZ (A) Ki67, BrdU, and GFAP expression are shown in coronal sections of Tam-Idh1-KI and control brains. Dashed lines outline the LV, around which the SVZ usually forms a ribbon of cells. The data are quantitated in the bar chart below. (B) Cells expressing oligodendocyte (Olig2^+^) and neuroblast (Dcx^+^) markers are shown. The data are quantitated in the bar chart below. All data are presented as mean ± SD (^∗^p < 0.05, ^∗∗^p < 0.01, ^∗∗∗^p < 0.005). See also Figure S3.

The confines of the SVZ and neuroblast migration in the healthy brain are tightly controlled, with the high density of cells making it easy to distinguish these regions from adjacent tissue. Migratory SVZ cells mostly move to the olfactory bulb (OB), with few emigrating from the SVZ or RMS to the adjacent parenchyma (Lois and Alvarez-Buylla, 1994). The Tam-Idh1-KI SVZ was enlarged, and its border was not clearly delineated (Figures 2 and 3). As expected, in controls, the great majority of BdU^+^, Ki67^+^, or EdU^+^ cells were restricted to the SVZ (Figures 4A, S3A, and S3B). In contrast, Tam-Idh1-KI mice contained large numbers of actively mitotic Ki67^+^ or EdU^+^ cells, as well as label-retaining BrdU^+^ cells, adjacent to and contiguous with the SVZ, in locations equivalent to those of the striatum and corpus callosum in wild-type mice (Figures 4A and S3). Similarly, Tam-Idh1-KI brains displayed increased numbers of GFAP^+^ cells in both the SVZ and surrounding parenchyma, whereas GFAP expression was almost exclusive to the SVZ and corpus callosum in controls (Figures 4A, S3A, and S3B). BrdU^+^ label-retaining GFAP^+^ astrocytes that were actively proliferating (Ki67^+^) were present in the striatum of Tam-Idh1-KI brains but absent in control mice (Figures 4A, S3A, and S3B). These data were consistent with Tam-Idh1-KI causing the SVZ to expand and/or SVZ cells to migrate out of their local boundaries and proliferate in ectopic locations adjacent to the neurogenic niche.

To further understand these regions of hyperproliferation, we assessed expression of Dcx, a marker of immature migratory SVZ neuroblasts, and Olig2. Compared with controls, Tam-Idh1-KI mice contained significantly more Dcx^+^ cells and Olig2^+^ cells in the striatum and corpus callosum (Figures 4B and S3C–S3E). Higher magnification showed that many of the ectopic Dcx^+^ cells had bipolar morphology with leading processes, suggestive of migration. Transformed cells and SVZ cells in pathological conditions can express multiple lineage markers (Jablonska et al., 2010), but the proportion of cells that were Dcx^+^/Olig2^+^ was small and did not change in the Tam-Idh1-KI mice (Figure 4B).

**Figure 5:**
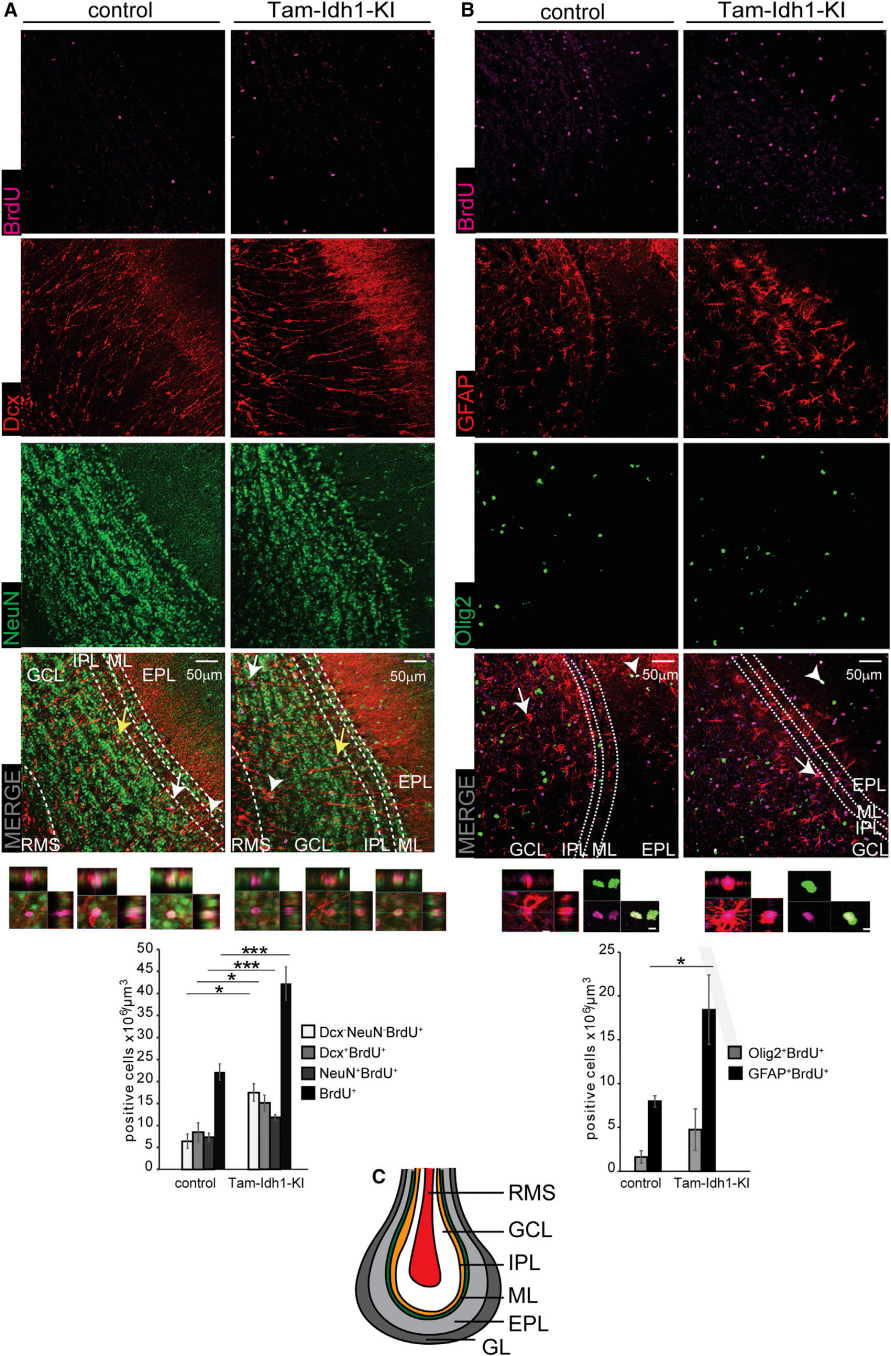
The Effect of *Idh1*^R132H^ on Neurogenesis and Astrocyte Genesis in the Olfactory Bulb (A) BrdU, Dcx, and NeuN expressing cells (neurogenesis) are shown in representative images from Tam-Idh1-KI (n = 4) and control (n = 4) OBs. The left-hand image at the bottom of each panel is a magnified orthogonal 3D view of the BrdU^+^Dcx^−^NeuN^-^ cell marked by a white arrow; the adjacent middle image is the equivalent view of the BrdU^+^Dcx^+^NeuN^−^ cell marked by a white arrowhead; and the adjacent right-hand image is the equivalent view of the BrdU^+^Dcx^−^NeuN^+^ cell marked by a yellow arrow. RMS, rostral migratory stream; GCL, granule cell layer; IPL, internal plexiform layer; ML, mitral layer; EPL, external plexiform layer. The cell counts are quantitated in the bar chart. (B) BrdU, GFAP, and Olig2 (astrocyte genesis) data are shown from the same mice as in (A). The left-hand image at the bottom of each panel is a magnified orthogonal 3D view of the BrdU^+^GFAP^+^ cell marked by a white arrow; and the adjacent right-hand image is the equivalent view of the BrdU^+^Dcx^−^NeuN^+^ cell marked by a white arrowhead. Other annotations are as per (A). (C) A schematic of the multi-layered cellular architecture of a mouse OB is shown. GL, glomerular layer. Other annotations are as per (A). All data are presented as mean ± SD (^∗^p < 0.05, ^∗∗∗^p < 0.005). See also Figure S4.

Migration in the RMS is highly regulated, similar to the SVZ. All seven Tam-Idh1-KI mice analyzed (five old and two young) had a more diffuse RMS (Figure 5A), with dispersed Dcx^+^ cells migrating in various orientations. In addition, in contrast to controls, all Tam-Idh1-KI mice examined exhibited Dcx^+^ cells, sometimes in massive numbers, in the ventral cerebral cortex, dorsal to the RMS, suggesting emigration from it (Figure 5A). This was consistent with their ectopic location near the dorsolateral SVZ (Figure 4). Additional abnormalities consistent with increased stem-like cells and aberrant migration were also found in the OBs of Tam-Idh1-KI animals (Figures 5, S4A, and S4B). Compared with controls, Tam-Idh1-KI animals contained significantly more BrdU^+^, Dcx^+^BrdU^+^, and NeuN^+^BrdU^+^ cells in the granule layer (data not shown) and the OB as a whole (Figure 5A), indicating that neurogenesis was increased in the Tam-Idh1-KI mice, probably due to the increased numbers of stem and/or proliferating SVZ cells. The proportion of BrdU^+^ cells that expressed differentiation/lineage markers (NeuN, Dcx, GFAP, and Olig2) was similar in controls and mutants, suggesting that the cells retained much of their normal differentiation capacity (data not shown).

Interestingly we also found that the OBs of Tam-Idh1-KI mice contained a larger number of BrdU^+^ cells that were negative for neuronal markers (Figure 5A), suggesting that glial cells may have been generated in the OB. To study this possibility, we quantified GFAP^+^ astrocytes and Olig2^+^ oligodendrocytes that retained BrdU. GFAP^+^BrdU^+^ cell numbers were significantly increased in the OBs of Tam-Idh1-KI mice, and the number of Olig2^+^ cells was also increased, albeit not significantly (Figure 5B).

To confirm that the SVZ was the source of the mislocalized cells, we traced the *Nes-Cre*-expressing cell lineage using control and Tam-Idh1-KI mice that also carried the Rosa26-YFP reporter allele (Srinivas et al., 2001). As expected in the control crosses, YFP^+^ cells were restricted to regions of endogenous nestin expression: the SVZ and RMS (Figures S4C–S4E). In the crossed Tam-Idh1-KI mice, many YFP^+^ cells were found in an expanded SVZ and RMS, and also in ectopic locations continuous with the neurogenic niche (Figures S4C–S4E). These included the corpus callosum, striatum next to the SVZ as well as regions adjacent to the RMS. This lineage experiment supported the model that the SVZ and RMS expansion and infiltration into adjacent brain regions was initiated from nestin-positive cells, which were originally most likely present in the SVZ.

Disruption of the glial tubes composed of B1 astrocytes and B2 niche astrocytes is often characteristically associated with abnormal migration of neuroblasts (Comte et al., 2011). To address this, we examined the RMS with GFAP IF and compared it with the distribution of YFP^+^ cells. Although the YFP^+^ cells were expanded in the RMS of mutant animals, we did not find that the glial tubes were altered in morphology in mutant mice compared with controls (data not shown). This suggests that the emigration/infiltration of the neuroblast progenitors, as well as the glial progenitors, into the regions adjacent to RMS and SVZ is probably cell autonomous.

These results suggest that expression of *Idh1*^R132H^ in adult mouse stem/progenitor cells robustly induced expansion of the SVZ and RMS and infiltration into adjacent regions, similar to observations of glioma cell invasion. The SGZ showed similar features (Figure S4F). While cell numbers were increased in the *Idh1*-mutant animals, the cells appeared to retain their capacity for differentiation.

### Tam-Idh1-KI Mice Develop Proliferative Subventricular Nodules

**Figure 6:**
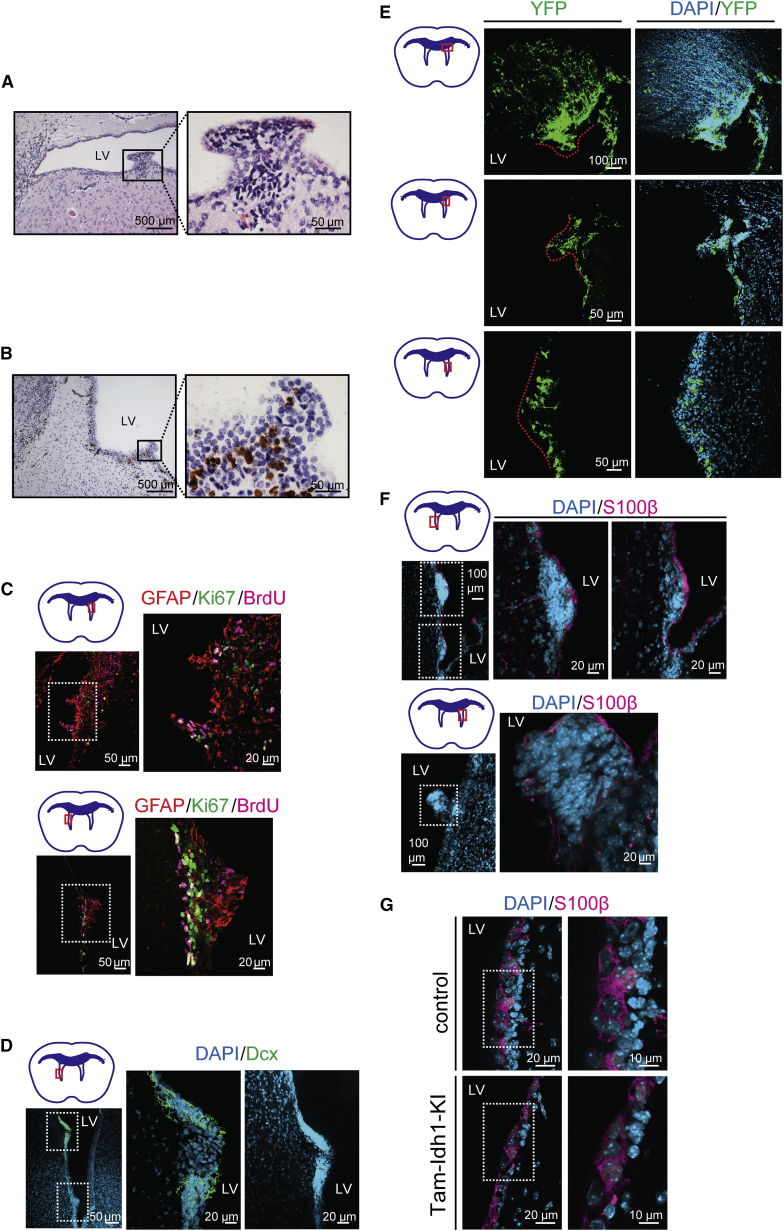
Nodule Formation in the SVZ (A) Typical morphology of a nodule protruding into the lateral ventricle (LV) is shown in an H&E-stained section from a Tam-Idh1-KI animal. (B) Ki67 expression was assessed in a nodule and at other sites along the LV wall. (C) Nodule proliferation was assessed by immunofluorescence for Ki67 and BrdU. Two nodules are shown (left), magnified in the inset (right). The astrocyte lineage marker GFAP is also shown. (D) Immature neuroblast differentiation in nodules was assessed using Dcx. (E) The origin of nodules from nestin-expressing cells was assessed using the YFP reporter. The dashed lines outline the nodules. YFP^+^ cells infiltrating the corpus callosum are also marked. (F) Ependymal cell-specific S100β expression indicates the extent of the continuity of the ependymal cell layer overlying the nodules and potential origins of the nodules from ependymal cells. Three nodules are shown (left), magnified in the insets (right). (G) A high-power view compares the ependymal cell layer in Tam-Idh1-KI and control mice.

In all Tam-Idh1-KI mice studied, apart from three killed only 2, 3, and 6 weeks after tamoxifen administration, we found small (up to 1 mm diameter) nodules originating from the SVZ and protruding into the LVs (Figure 6A). The lesions, which were absent from control animals, could be found at any location in the walls of the LVs. The nodules expressed proliferation markers, such as Ki67 (Figures 6B and 6C), and retained BrdU label (Figure 6C), suggesting that they exhibited a variety of proliferative behaviors. As shown in Figure 6C, several cells present in the nodules were Ki67^+^BrdU^+^, indicative of label-retaining cells that had re-entered the cell cycle. Many nodule cells also expressed the astrocytic and NSC marker GFAP and in some lesions, a few cells expressed the neuroblast marker Dcx (Figures 6C and 6D). Using a reporter mouse cross, we found that the nodules expressed YFP, consistent with origins from nestin-expressing stem or TAP SVZ cells (Figure 6E). Microscopic inspection of the nodules revealed clustered cells, with hyperchromatic nuclei and scant cytoplasm that were clearly distinct from the overlying ependymal lining (Figure 6A). Some of the nodules were not continuously covered by S100β^+^ ependymal cells, suggesting that subventricular lesions had broken through the ventricular lining (Figure 6F). The ependymal layer, as assessed by S100β expression, otherwise appeared normal (Figure 6G).

### The *Idh1*^*fl(*R132H*)/+*^ Allele Is Leaky and Causes a Brain Phenotype in a Minority of Juvenile or Aged Mice

**Figure 7:**
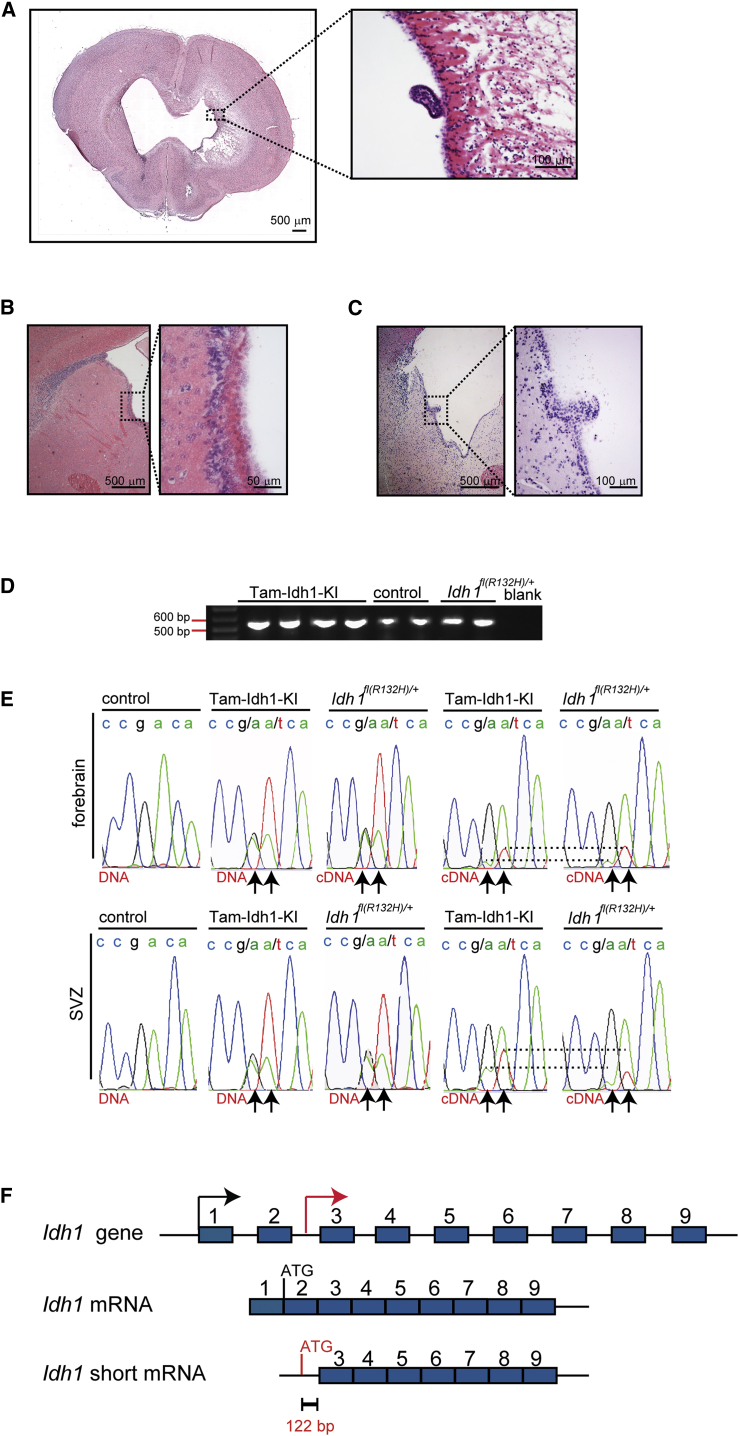
Leaky Phenotype in a Minority of *Idh1*^*fl(*R132H*)/+*^ Mice without Nes-Cre (A) A coronal full-brain section from a symptomatic *Idh1*^*fl(*R132H*)/+*^ mouse 6 weeks old is shown. A nodule is outlined and magnified. (B) Detailed SVZ morphology of an asymptomatic 4 month old *Idh1*^*fl(*R132H*)/+*^mouse is shown (sagittal section). (C) A nodule is present in an asymptomatic 13 month old mouse (sagittal section). (D) The *Idh1* transcript lacking exons 1 and 2 was amplified from cDNA by endpoint RT-PCR (amplicon size 562 bp). (E) DNA sequencing chromatogram shows genomic DNA or cDNA specifically derived from the short transcript in the *Idh1* region around codon 132 (samples from mouse forebrain). Arrows indicate nucleotides altered in Tam-Idh1-KI and *Idh1*^*fl(*R132H*)/+*^; dashed lines provide an indication of the relative dosages of wild-type and mutant short transcript in each case. (F) Schematic of short in-frame *Idh1* transcript lacking exons 1 and 2 shows the putative translational origin in intron 2.

We had noted that 10% (9/94) of *Idh1*^*fl(*R132H*)/+*^ mice without the Nes-Cre transgene and 8% (5/62) of non-induced *Nes-Cre*^*ER(T2)*^*;Idh1*^*fl(*R132H*)/+*^ animals developed rounded and enlarged skulls at 3–6 weeks of age. This phenotype, reminiscent of human hydrocephalus prior to fusion of the skull sutures, necessitated immediate culling. Ventricular nodules, similar to those in Tam-Idh1-KI mice, were found in these animals (Figure 7A.) We aged some surviving *Idh1*^*fl(*R132H*)/+*^ animals (after tamoxifen injections) for 1–2 years. Although none showed symptoms or signs of disease while alive, upon postmortem investigation, 8/34 animals (24%) had ventricular enlargement. Of those eight mice, histological examination showed one to have a clearly enlarged, diffuse SVZ (Figure 7B) and another to have a single subventricular nodule (Figure 7C). The brains of these mice accumulated 2HG, but there was no evidence of other brain damage (data not shown). We also aged three *Nes-Cre*^*ER(T2)*^*;Idh1*^*fl(*R132H*)/+*^ animals that had not received tamoxifen, and all showed a similar phenotype to the eight *Idh1*^*fl(*R132H*)/+*^ mice without Nes-Cre. Further investigation strongly suggested that the phenotype resulted from expression of an *Idh1* mRNA that lacked exons 1 and 2 and was derived from the mouse construct (Figures 1A, 7D, and 7E). We found that the short RNA was a physiological isoform, as it was also produced by the wild-type *Idh1* allele. *Idh1* exons 1 and 2 have no homology to any other protein, are not conserved, and contain no functional domains of predicted importance. A search of the genomic DNA sequence revealed a potential translation initiation site in intron 2 that would leave the enzyme active site intact (Figure 7F). We conclude that in a minority of mice, a leaky *Idh1*^R132H^ allele can produce a forme fruste of the full Tam-Idh1-KI phenotype.

### Identifying the Molecular Mechanism Underlying the Tam-Idh1-KI Phenotype

**Figure 8:**
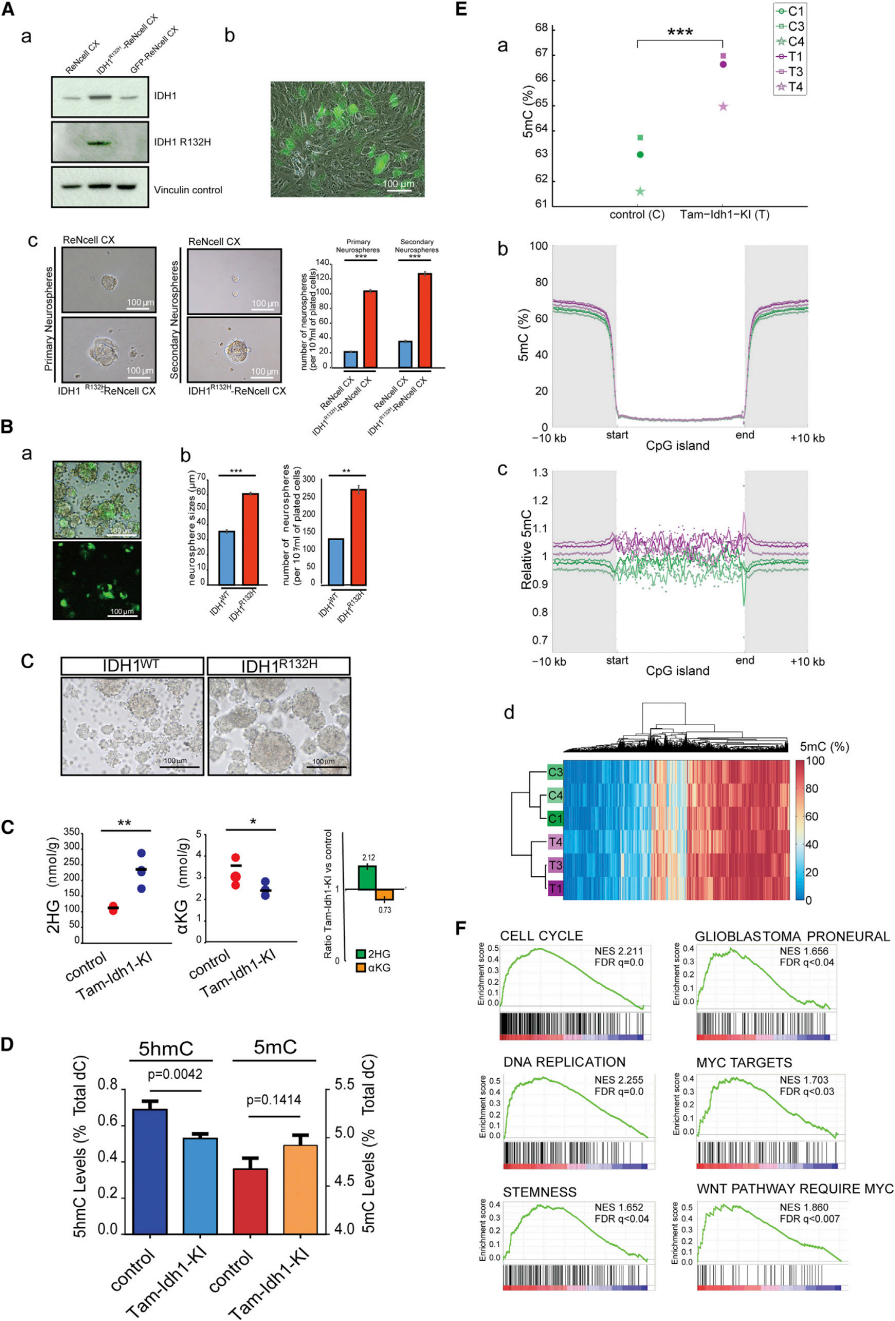
Assessment of Functional Mechanisms Underlying the Tam-Idh1-KI Phenotype (A) The experiments determined the effects of IDH1^R132H^ expression in ReNcell CX neuronal progenitor cells. (a) western blot shows expression of total and mutant IDH1 in cells transduced by IDH1^R132H^ or GFP lentiviral vectors compared with untransduced cells. (b) GFP expression indicates transduction efficiency. (c) Representative bright-field images are shown for ReNcell CX cells (IDH1^R132H^-transduced or untransduced controls) grown as primary or secondary neurospheres at low density. The chart shows quantification of neurospheres grown at a low density. Data are representative of three independent experiments, one using high and two using low density cultures, giving consistent results. Results are presented as means ± SEM (^∗∗∗^p < 0.005). (B) Tertiary neurospheres were derived from primary SVZ cells transduced with lentiviruses expressing IDH1 wild-type (WT) or R132H. (a) GFP expression shows representative transduction efficiency. (b) After 4 days in culture, the size and number of WT and IDH1^R132H^ neurospheres were measured to assess proliferation and self-renewal capacity. The chart shows a representative experiment of three independent experiments, two using high and one using low density cultures. Results are presented as means ± SEM (^∗∗^p < 0.01, ^∗∗∗^p < 0.005). (c) Representative images of IDH1-wild-type and IDH1^R132H^ neurosphere cultures grown at high density are shown. (C) Mass spectrometric assessment of the effect of *Idh1*^R132H^ on 2HG and αKG levels was performed. Brain tissue extracts from Tam-Idh1-KI mice (n = 4) and controls (n = 3) aged 4–9 months were assayed for total 2HG (D and L isomers). Data are shown as dot plots and summarized as relative expression in the bar chart, presented as means ± SD. ^∗^p < 0.05, ^∗∗^p < 0.01, from ANOVA. (D) Total 5hmC and 5mC levels of forebrain were assessed using denaturing HPLC. Data are presented as means ± SD. (E) 5mC genome sequencing was performed on SVZ DNAs. (a) Levels of cytosine methylation at CpG dinucleotides in Tam-Idh1-KI (T) and controls (C) paired by age and sex are shown, ^∗∗∗^p = 0.0163, from Kolmogorov-Smirnov test. (b, c) Absolute (b) and relative (c) differences in CpG methylation within CpG islands and flanking regions. (d) Hierarchical cluster analysis of top 2,000 differentially methylated CpGs to assess whether mutants and controls clustered separately. (F) mRNA expression GSEA analysis is shown for selected gene sets enriched in Tam-Idh1-KI brains. See also Figures S5 and S6, Table S1.

Attempts to culture primary *Idh1-*mutant neurospheres from the SVZ of Tam-Idh1-KI mice were unsuccessful. We therefore stably expressed IDH1^R132H^ both in human neuronal stem/progenitor cells (ReNcell CX) and in primary SVZ cells dissected from wild-type BL6 mouse pups. In each case, IDH1^R132H^ increased the size and number of neurospheres cultured from human NSC/NPCs (Figures 8A and 8B). These data were consistent with increased proliferation and self-renewal capacity of the mutant NSC/NPCs in vivo.

We also measured the capability of ReNcell CX expressing IDH1^R132H^ to migrate in vitro, in unstimulated or chemoattractant (20 μg/mL FGF or 5% serum) conditions. *IDH1*-mutant cells were significantly more motile than control cells (∼1.8-fold in all conditions; ANOVA, p < 0.0001; details not shown). These data were in accordance with the infiltrative behavior of *Idh1*-mutant NSC/NPCs in vivo.

Using capillary electrophoresis time-of-flight mass spectrometry (CE-TOFMS), we performed a comprehensive analysis of metabolites in Tam-Idh1-KI and control mice. In Tam-Idh1-KI samples, we detected ∼2-fold increase in total 2HG and ∼30% decreased αKG compared with controls (Figure 8C). The leaky mice showed similar changes but at lower levels (details not shown). The changes in adults appeared less substantial than those found in the brains of *Nes-Cre; Idh1*^*fl(*R132H*)/*+^ embryos using IC-MS (Figure S5A). No significant differences were detected in any other metabolite, including glycolytic, TCA cycle, and pentose phosphate pathway intermediates.

We found no clear evidence that collagen-PHDs, ROS, HIF-PHDs, or JHDMs were altered in the Tam-Idh1-KI mice (Figures S5B–S5F, Table S1). In contrast, in the forebrains of Tam-Idh1-KIs and controls, global abundance analysis by high-performance liquid chromatography (HPLC) showed significantly decreased 5hmC and raised 5mC (Figure 8D). Although the latter was formally non-significant, low-coverage single-base resolution analysis using methyl-seq (WGoxBS) showed significantly increased 5mC in the SVZ of Tam-Idh1-KI animals (Figures 8E and S6A–S6D). The greatest absolute increase in 5mC was outside CpG islands, although the relative increase was similar across all genomic regions (Figure 8E). In summary, *Idh1*^R132H^ mutation led to a global decrease of 5hmC and increase of 5mC, consistent with tumor-promoting effects.

### mRNA Expression Profiling in Tam-Idh1-KI Mice Identifies Pathways of Tumorigenesis

To uncover molecular pathways that might underlie the phenotype of the Tam-Idh1-KI mice, we performed transcriptional profiling of SVZ cells from mutant and control animals. Gene set enrichment analysis (GSEA) revealed several pathways and processes of note that were more active in Tam-Idh1-KIs than controls at a false discovery rate <0.05 (Figures 8F and S6E, Table S1). Notably, these included brain-tumor-associated processes matching the mouse phenotype such as stemness, cell cycle, maintenance of telomeres, and DNA replication. This analysis also showed significant increases in gene sets specific to human glioblastomas, including the proneural sub-type, which is characterized by mutation of *IDH1* (Verhaak et al., 2010) (Figures 8F and S6E). Interestingly, both Wnt and c-Myc target genes were also overexpressed in Tam-Idh1-KIs, suggesting potential molecular mechanisms for the gliomagenesis phenotype.

## Discussion

We have shown that conditional, inducible expression of the *Idh1*^R132H^ mutation in the adult mouse SVZ stem cell niche causes cellular and molecular features associated with brain tumorigenesis. We found three critical phenotypes in the SVZ niche: (1) increased numbers of label-retaining stem cells as well as acutely proliferating cells in and around the SVZ and RMS; (2) significant infiltration of neuronal and glial progenitors from the SVZ and RMS into surrounding regions; and (3) subventricular nodules containing proliferating stem/progenitor cells that protruded into the LV from the SVZ. The mutant animals developed dilation of the LVs, with accompanying distortion of the cerebrum and clinically apparent hydrocephalus. However, the brains showed little, if any, evidence of neural damage or degeneration and appeared largely normal histologically in regions away from the niche.

The phenotypic dissimilarities between our Tam-Idh1-KI and other *Idh1/2* models probably resulted from our targeting of *Idh1*^R132H^ to the neurogenic niches of the adult brain. Nestin-Cre causes recombination early in the SVZ lineage and can affect large numbers of NSCs (Lagace et al., 2007). However, nestin is also expressed in other SVZ cells, including TAPs and niche astrocytes (Doetsch et al., 1997), and all of these may have been influenced by the *Idh1* mutation. Given the relatively large expansion of quiescent NSCs and the overexpression of stem cell markers in Tam-Idh1-KI mice, our data suggest that an early stem cell was targeted by Nes-Cre. The leakiness of the *Idh1*^*fl(*R132H*)/+*^ allele may also have contributed to the Tam-Idh1-KI phenotype. Evidence strongly suggests that the phenotype caused by the leaky allele also originates in the SVZ. First, although the leaky allele causes 2HG accumulation in all tissues examined, no phenotype outside the brain was detected. Second, the phenotypes of Tam-Idh1-KI and leaky mice are very similar, notwithstanding much reduced penetrance and severity in the latter. Third, nestin expression is not induced by 2HG outside the SVZ and SGZ in the leaky mice; thus, while Tam-Idh1-KI mice are produced from an allele that is already leaky, when tamoxifen is given, full R132H knockin occurs in the SVZ and SGZ. Although leaky R132H expression in cells outside the SVZ could in theory explain the more numerous and misplaced cells in the striatum, corpus callosum, RMS, and OB in *Idh1* mutants, lineage tracing in Tam-Idh1-KIs showed that the cells undergoing recombination in the SVZ after tamoxifen administration could account for the great majority of these cells.

Tsc1-cKO (*Nes-Cre*^*ER(T2)*^; *Tsc1*^*loxP/loxP*^) mice also develop hydrocephalus, periventricular nodules, and tumor-like structures near the inter-ventricular foramen (Feliciano et al., 2012, Zhou et al., 2011). The Tsc1-KO lesions are Dcx^+^ and were mostly non-proliferative, in contrast to our mice. The causes of ventricular dilation and hydrocephalus were not identified in the Tsc1-KOs, although cryptic nodules obstructing CSF flow or disrupting the ependymal lining are possible causes. Mice overexpressing *Tlx*, a key regulator of NSC expansion, also develop periventricular nodules, accompanied by SVZ and RMS changes similar to the Tam-Idh1-KIs but without the gross phenotype of our animals (Liu et al., 2010). We found increased expression of Tlx targets in the Tam-Idh1-KI mice (Table S1).

Several theories as to how the *IDH1*^R132H^ mutation drives cancer were not supported by our work, including hypoxia pathway activation, increased angiogenesis, altered collagen, histone methylation, and ROS. We caution that the SVZ tissue analyzed may have contained some non-induced cells, diluting out effects. We did find that *Idh1*^R132H^ animals had raised 2HG and decreased αKG, accompanied by reduced 5hmC and increased global 5mC in DNA. *IDH2* and *TET2* mutations are mutually exclusive in AML (Figueroa et al., 2010, Gaidzik et al., 2012), and it has been reported that expression of mutant IDH or administration of 2HG can inhibit TET2 in vitro and in vivo, resulting in decreased production of 5hmC (Figueroa et al., 2010, Sasaki et al., 2012a, Xu et al., 2011) and reduced DNA demethylation (Pastor et al., 2013). TET activity might also be limited by reduced αKG availability. The increased DNA methylation in Tam-Idh1-KI brains is correspondingly present in human *IDH1-*mutant tumors (Guilhamon et al., 2013, Lu et al., 2012, TCGA, 2015, Sturm et al., 2012), but the pattern of changes appears to differ, the former being global and relatively uniform and the latter, targeting CpG islands (CIMP). It is of note that CIMP has often been measured using microarrays that focus on CpG islands, with much less coverage of other CpGs. Therefore, some cases scored as CIMP^+^ may actually possess a tendency to genome-wide methylation. Furthermore, a global methylator phenotype could cause CIMP by the selection of epimutations at CpG islands during tumorigenesis.

mRNA expression profiling showed Tam-Idh1-KI mice to have increased c-Myc and Wnt pathway activity, higher numbers of cycling cells, upregulated telomere maintenance, and more cells with a stem-like phenotype. Culture of human neuronal stem/progenitor cells supported the mouse data by showing increased self-renewal and proliferation in cells expressing *IDH1*^R132H^. All of these functional processes are strongly mechanistically associated with tumorigenesis. Tam-Idh1-KI mice also expressed high levels of genes associated with *IDH1*-mutant human brain tumors.

Together, our findings show that expression of mutant *Idh1* recapitulates cellular and molecular features of gliomagenesis, including stem cell features, increased proliferation, infiltration into adjacent structures, and tumor-like nodules originating from nestin-expressing cells. Our data suggest that the underlying cellular defect in the Tam-Idh1-KI mice is an increased and poorly controlled number of dividing stem/progenitor cells that retain much of their differentiation capacity, thus potentially representing the earliest phase of IDH-driven gliomagenesis. Our mice provide insights into human *IDH1-*driven brain tumors and a model system for assessing therapies.

## Experimental Procedures

Procedures are detailed in the Supplemental Experimental Procedures and summarized here. All mouse experiments were performed in accordance with institutional and national guidelines and regulations under UK Home Office Project Licence PPL 3003311. For the Tam-Idh1-KI model, mice received intraperitoneal tamoxifen injections to induce Nes-Cre-mediated recombination, causing the R132H protein to be expressed. For analysis of mRNA and proteins, snap-frozen tissue was obtained for analysis after sectioning brains and carefully removing the SVZ or other region under a dissecting microscope. For fixed tissue analysis, mice were transcardially perfused with normal saline and 4% paraformaldehyde (PFA). Brains were removed, post-fixed in 4% PFA, cryoprotected in 30% sucrose, and frozen. Sections were cut on a sliding microtome, and kept at −20°C. Sections were stained using standard free-floating IHC. Numbers of immune-positive cells were counted in confocal microscopy z stacks. All quantifications were done by an observer blinded to mouse genotypes.

Molecular assays were performed using standard protocols for (1) 5mC and 5hmC (HPLC and next-generation sequencing), (2) TCA cycle and other metabolites (mass spectrometry); and (3) mRNA expression profiling (Illumina Mouse WG6-v2 microarrays). Western blotting was performed to assess the expression of selected proteins (hypoxia, ROS, histone modification, collagen), supplemented by qRT-PCR for mRA where appropriate.

For all phenotypic assessments, including molecular studies, tamoxifen-treated, Tam-Idh1-KI animals were compared with paired, tamoxifen-treated, littermate controls (*Idh1*^*+/+*^, with or without *Nes-Cre* transgene). Cell lineage and proliferation analysis was performed using immunofluorescence for DNA labeling agents (BrdU, EdU), proliferation marker (Ki67), and markers of differentiation (GFAP, Olig2, NeuN, Dcx, S100β, Pdgfrα). Assessment of tissue morphology was performed using H&E-stained sections, supplemented by IHC for selected markers.

Assays to assess the effect of *IDH1*^R132H^ were performed by expressing the mutant protein in ReNcell CX from human fetal cerebral cortex or neural stem/progenitor cells from P4 wild-type mice. Assays performed on these cells included primary and second neurosphere formation and transwell motility.

All data are presented as means ± SDs, with nominal p values from unpaired t tests, unless specified otherwise in the figure legend.

## Author Contributions

Planned experiments: C.B., O.A.D., J.A., P.J.P., J.S.M., C.J.S., S.K., P.J.R., F.G.S., I.T. Performed experiments and analyses: C.B., O.A.D., O.A., D.K., J.A., P.J.P., K.A., I.P., L.F.M., J.S.M., H.L., S.S., N.S., R.G., T.S., O.A., P.B., M.T., F.G.S., and I.T. Wrote the manuscript: C.B., O.A.D., P.J.R., F.G.S., and I.T. Oversaw the study: C.J.S., P.M., O.A., B.S.-B., S.K., P.J.R., F.G.S., and I.T.

## Accession Numbers

Gene expression data are available at GEO: GSE85080.

## Supplemental Information

Document S1. Supplemental Experimental Procedures and Figures S1–S6

Table S1. Gene Set Enrichment Analysis of mRNA Expression Profiling in Tam-Idh1-KI and Control SVZs, Related to Figure 8

Document S2. Article plus Supplemental Information
