# Supplementary material for: Expression of Idh1R132H in the Murine Subventricular Zone Stem Cell Niche Recapitulates Features of Early Gliomagenesis
Source: Cancer Cell. 2016 Oct 10;30(4):578–94. doi: 10.1016/j.ccell.2016.08.017 (PMC5064912; doi:10.1016/j.ccell.2016.08.017)
Supplement: Document S1. Supplemental Experimental Procedures and Figures S1–S6 [file mmc1.pdf]

Cancer Cell, Volume *30*

Supplemental Information

Expression of *Idh1*^{R132H} in the Murine Subventricular Zone Stem Cell Niche Recapitulates Features of Early Gliomagenesis

Chiara Bardella, Osama Al-Dalahmah, Daniel Krell, Pijus Brazauskas, Khalid Al-Qahtani, Marketa Tomkova, Julie Adam, Sébastien Serres, Helen Lockstone, Luke Freeman-Mills, Inga Pfeffer, Nicola Sibson, Robert Goldin, Benjamin Schuster-Böeckler, Patrick J. Pollard, Tomoyoshi Soga, James S. McCullagh, Christopher J. Schofield, Paul Mulholland, Olaf Ansorge, Skirmantas Kriaucionis, Peter J. Ratcliffe, Francis G. Szele, and Ian Tomlinson

SUPPLEMENTAL DATA

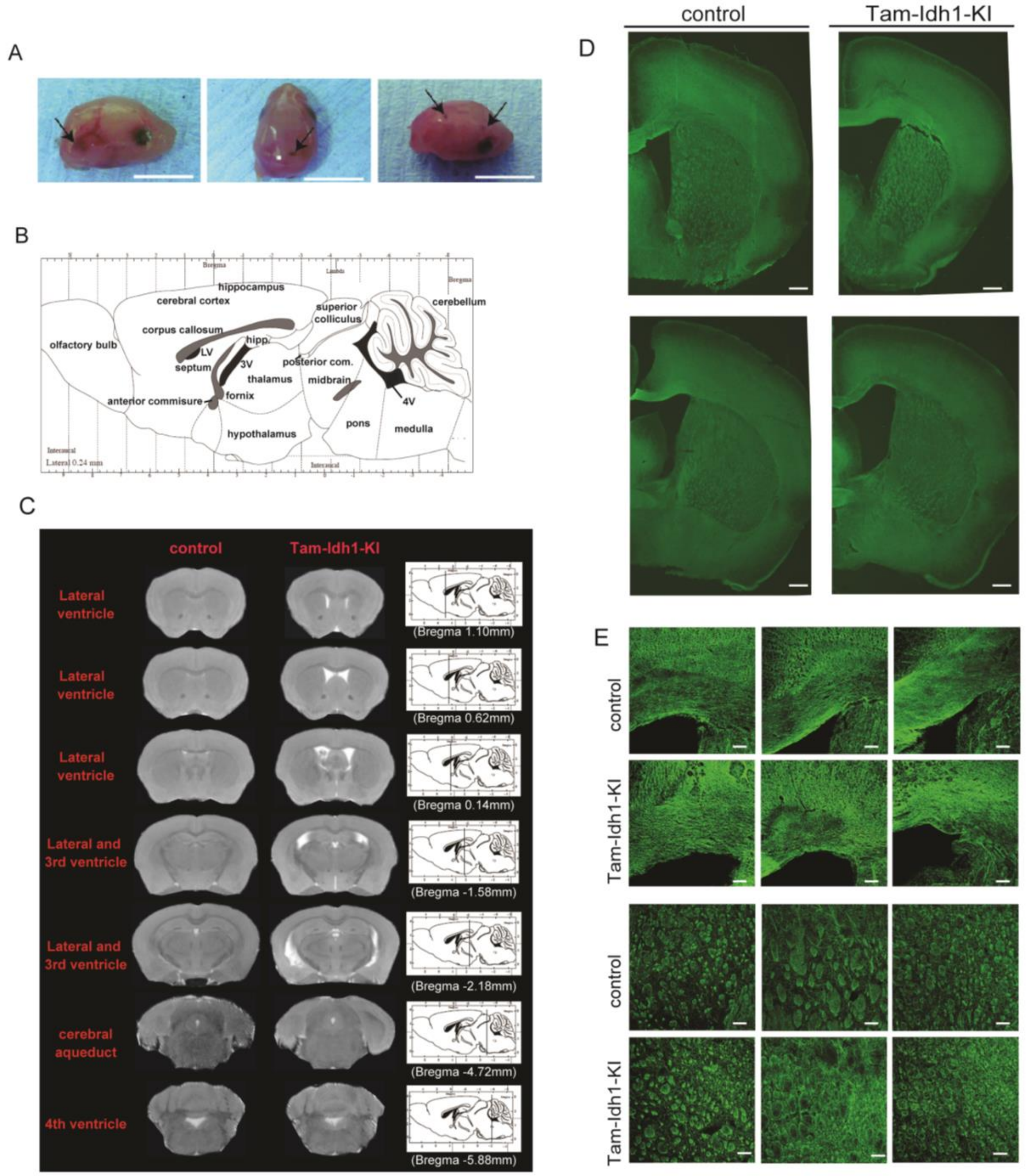

Figure S1 related to Figure 1: Phenotypes caused by expression of *Idh1*^{R132H} in the embryonic and adult brain.

A. Dorsal and lateral views of embryonic mouse heads dissected from Idh1-KI animals.
Areas of hemorrhage in these animals, derived from an *Idh1*$^{fl(R132H)/+}$ *x* Nes-Cre cross, are shown (black arrows). Scale bars 5 mm.

B. Schematic of sagittal section of mouse brain.
A section of the mouse brain is taken in sagittal section 0.24 mm lateral to the midline. Major structures are indicated. Ventricles are shown in black. Modified from "The Mouse Brain" by G. Paxinos and K.J.B Franklin (Elsevier, 2015, ISBN: 978-0-12-391057-8).

C. Sequential coronal images of MRI scans of Tam-Idh1-KI (adult) and control brains.
The images shown are at comparable positions from Tam-Idh1-KI and control mice. Bregma coordinates for each MRI image and the corresponding sagittal position are shown with vertical lines.

D. Myelin basic protein (MBP) in coronal Tam-Idh1-KI brain sections compared with controls using immunofluorescence. MBP is a marker of mature oligodendrocytes and brain damage. Scale bars are 500 µm.

E. MBP expression in the corpus callosum (middle panels) and striatum (bottom panels) of Tam-Idh1-KI mice and controls. Scale bars 100 µm.

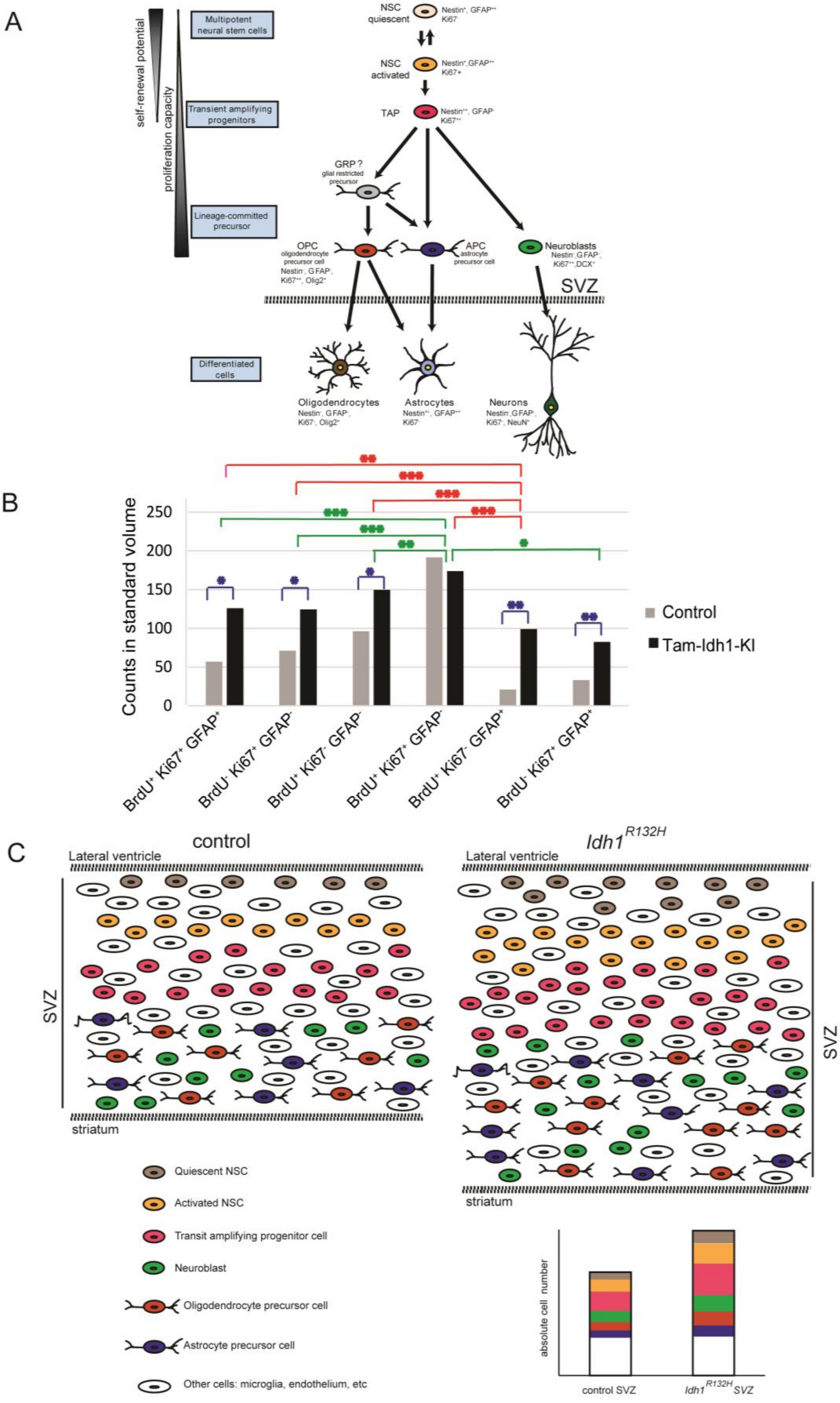

A
self-renewal potential
proliferation capacity
Multipotent neural stem cells
Transient amplifying progenitors
Lineage-committed precursor
Differentiated cells
NSC quiescent
NSC activated
TAP
GRP ?
glial restricted precursor
OPC
oligodendrocyte precursor cell
APC
astrocyte precursor cell
Neuroblasts
SVZ
Oligodendrocytes
Astrocytes
Neurons
B
Counts in standard volume
Control
Tam-Idh1-KI
BrdU+ Ki67+ GFAP+
BrdU- Ki67+ GFAP-
BrdU+ Ki67- GFAP-
BrdU+ Ki67+ GFAP-
BrdU+ Ki67- GFAP+
BrdU- Ki67+ GFAP+
C
control
Idh1 R132H
Lateral ventricle
SVZ
striatum
Quiescent NSC
Activated NSC
Transit amplifying progenitor cell
Neuroblast
Oligodendrocyte precursor cell
Astrocyte precursor cell
Other cells: microglia, endothelium, etc
absolute cell number
control SVZ
Idh1 R132H SVZ

Figure S2 related to Figure 3. Canonical model of the adult mouse SVZ cell lineages and detailed analysis of lineage changes in the SVZ of Tam-Idh1-KI mice.

A. The SVZ is well characterized and has become a major system for studying mechanisms of neural development and tumorigenesis. Slowly dividing NSCs give rise to frequently dividing transit amplifying progenitor cells (TAPs), which in turn generate lineage-committed immature neuroblasts or glioblasts (OPCs and APCs). SVZ "niche astrocytes" (not shown) do not exhibit stem cell features and physically separate neuroblasts from the surrounding parenchyma. Ependymal cells (not shown) line the lateral ventricles, forming the cerebrospinal fluid–brain barrier, and surround apical primary cilia of NSCs (Doetsch et al., 1999; Mamber et al., 2013; Mirzadeh et al., 2008; Sajad et al., 2011; Silva-Vargas et al., 2013). The neuroblasts move in the rostral migratory stream (RMS) from the SVZ to the olfactory bulbs (OBs), where they differentiate into functional interneurons (Lois and Alvarez-Buylla, 1994). In addition to neurons, the adult SVZ also produces a small number of astrocytes and oligodendrocytes, the latter via resident oligodendrocyte progenitor cells. Neurogenesis in the adult human SVZ has been controversial (Curtis et al., 2007; Sanai et al., 2011), but was recently confirmed by a series of retrospective analyses of nuclear bomb test [^{14}C]-labelling of adult-born human neurons (Ernst et al., 2014). Based on these data, it is likely the human SVZ generates cells that constitutively migrate to the adjacent striatum rather than to the OB as in rodents (Ernst et al., 2014). This makes it more likely that tumors derived from SVZ cells also infiltrate structures adjacent to the lateral ventricle, such as the striatum.

B. We postulated that the *Idh1*^{R132H} mutation could act in two ways: (i) changing total numbers of cells; and/or (ii) changing the proportions of each cell type. Importantly, SVZ volume was larger in mutants, whereas overall cellular density was not significantly changed. Total SVZ cell numbers were therefore increased in the mutants. It also follows that the density of any cell type can be used as a measure of its proportion in the cell population, but *not* as a measure of total numbers of those cells. More specifically, cell types with increased density in mutants must also have increased total numbers, but cells with decreased density may have lower, unchanged or somewhat increased numbers (because the size of the expanded SVZ must be taken into account). Although we could not simultaneously assess large numbers of cell lineage markers using IF, we were able to count cells for 6 of the possible 8 combinations of BrdU, Ki67 and GFAP:

BrdU$^+$/Ki67$^+$/GFAP$^+$ - label-retaining GFAP$^+$ cells that had re-entered the cycle, most likely quiescent NSCs becoming activated NSCs;

BrdU$^-$/Ki67$^+$/GFAP$^-$ - one or more of TAPs, oligodendrocyte precursors and neuroblasts;

BrdU$^+$/Ki67$^-$/GFAP$^-$ - "other" stem-like or slowly dividing cells of uncertain type;

BrdU$^+$/Ki67$^+$/GFAP$^-$ - other label-retaining cells of uncertain type, such as endothelial cells or microglia, which had re-entered the cell cycle;

BrdU$^+$/Ki67$^-$/GFAP$^+$ - probably quiescent NSCs;

BrdU$^-$/Ki67$^+$/GFAP$^+$ - activated NSCs, or possibly cycling niche astrocytes (but not ependymal cells as these were Ki67$^-$); and

BrdU$^-$/Ki67$^-$/GFAP$^-$ and BrdU$^-$/Ki67$^-$/GFAP$^+$ - could not be assessed for technical reasons.

Some of the above counts were obtained indirectly (e.g. BrdU$^+$/Ki67$^-$/GFAP$^+$ was calculated by subtracting BrdU$^+$/Ki67$^+$/GFAP$^+$ counts from BrdU$^+$/GFAP$^+$ counts). Significant differences in the proportions of cells in Tam-Idh1-KIs and controls are shown in blue (*$p<0.05$). We then assessed whether each cell type was affected to a similar extent by the *Idh1*^{R132H} mutation. For this, we used a contingency table approach, in which columns contained each of the 6 cell types, and rows comprised mutant or wildtype mice. To obtain standardized data, we rebased cell type counts for each mouse to an SVZ volume of 750,000 μm^3 (as this was the smallest SVZ volume assessed in any animal). With the caveat that BrdU$^-$/Ki67$^-$/GFAP$^+$ and BrdU$^-$/Ki67$^-$/GFAP$^-$ populations could not be assessed in this experiment, pairwise assessments of each of the 6 cell types were performed to determine which populations were differentially affected by the *Idh1* mutation (significant results shown in red and green; *$p<0.05$, **$p<0.01$, ***$p<0.005$).

C. A schematic summary of the SVZ cell proliferation and lineage analysis is shown. Numbers and proportions of quiescent NSCs, activated NSCs, TAPs, oligodendrocyte precursors, neuroblasts and putative astrocyte precursors (shown in colors) were directly assessed. Changes in the density/proportion and numbers of other cell types (shown in white) were deduced from measurements of total cell numbers, although the identities of these cells are not well defined. For simplicity, we have not reproduced the complex cytoarchitectural arrangement of SVZ cell types. The bar chart denotes the absolute numbers of each cell type in the SVZs of control and Tam-Idh1-KI mice.

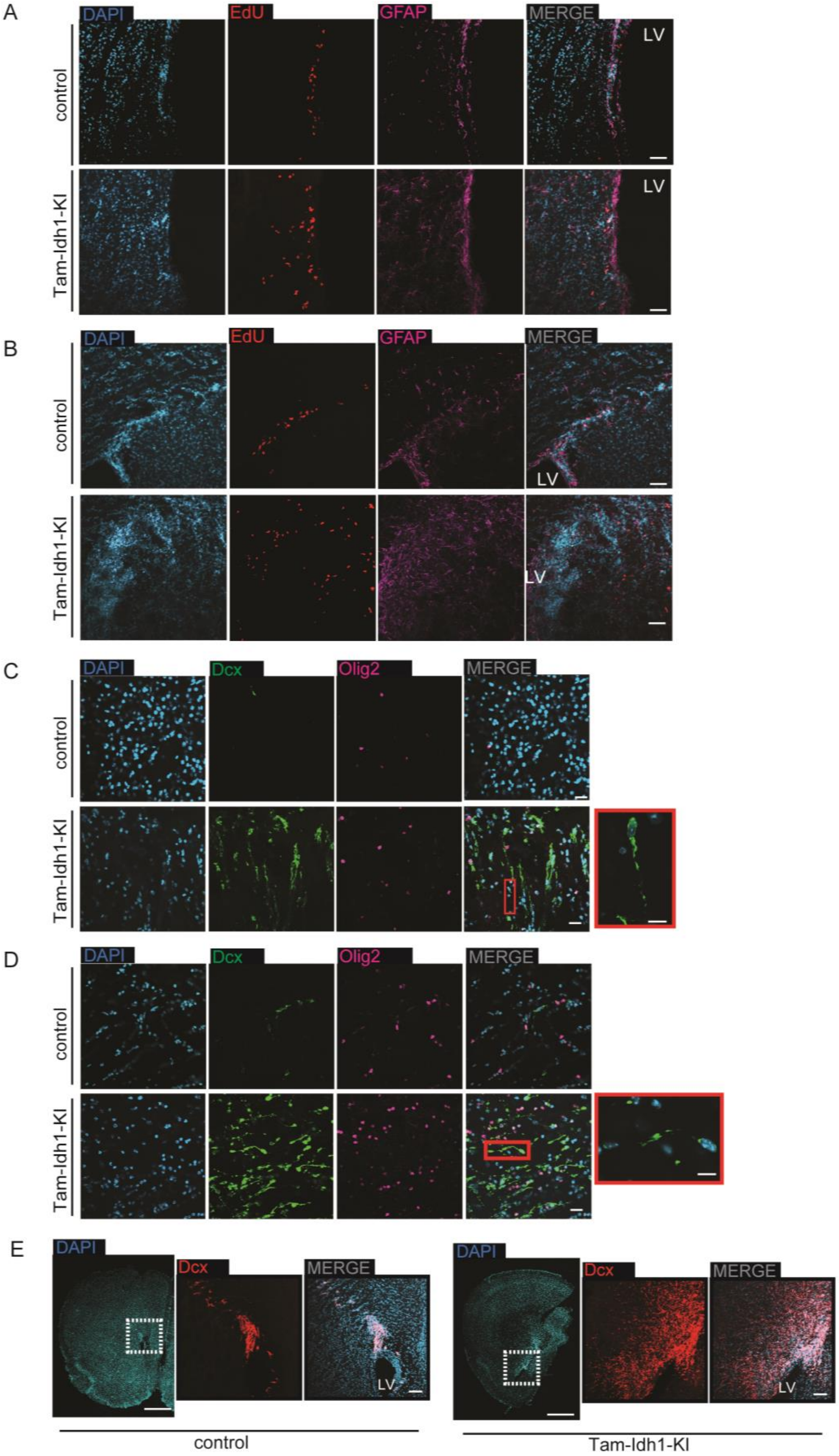
A
DAPI
EdU
GFAP
MERGE
LV
control
Tam-Idh1-KI
B
DAPI
EdU
GFAP
MERGE
LV
control
Tam-Idh1-KI
C
DAPI
Dcx
Olig2
MERGE
control
Tam-Idh1-KI
D
DAPI
Dcx
Olig2
MERGE
control
Tam-Idh1-KI
E
DAPI
Dcx
MERGE
LV
control
Tam-Idh1-KI

Figure S3 related to Figure 4. Further cell lineage analysis in tissues surrounding the SVZ.

A. Ectopic proliferating (EdU^+) cells of the astrocyte lineage ($GFAP^+$) in the striatum of Tam-Idh1-KI mice compared with controls.
LV: lateral ventricles. The area within the red box is also shown magnified. Scale bars 50 µm. Sagittal sections.

B. Ectopic proliferating (EdU^+) cells of the astrocyte lineage ($GFAP^+$) in the corpus callosum of Tam-Idh1-KI mice compared with controls.
Annotation is as per (A).

C. Immature neuroblasts (Dcx^+) and oligodendrocytes ($Olig2^+$) in the striatum of Tam-Idh1-KI mice compared with controls.
Scale bars 20 µm. In the red squares, Dcx^+ cells, marking migratory neuroblasts with extended leading processes are shown in normal and magnified views. Scale bars 10 µm. Sagittal sections.

D. Immature neuroblasts (Dcx^+) and oligodendrocytes ($Olig2^+$) in the corpus callosum of Tam-Idh1-KI mice compared with controls.
Annotation is as per (C).

E. Neuroblasts emigrating from the dorsolateral corner of the SVZ in Tam-Idh1-KI brains compared with controls.
Areas in the inset are magnified. LV: lateral ventricle. Scale bars 1.5 mm and 100 µm. Coronal sections.

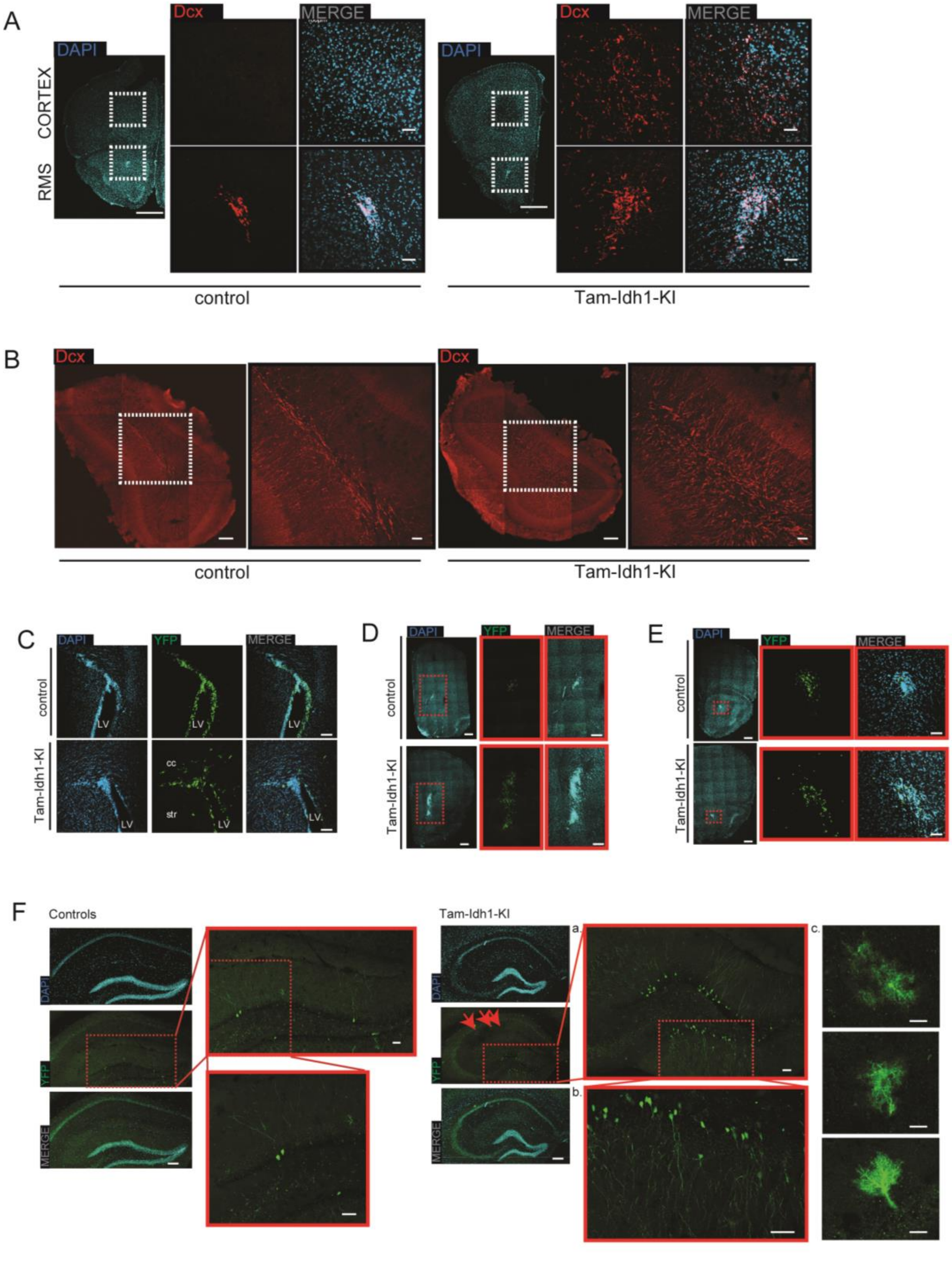

A
DAPI
Dcx
MERGE
CORTEX
RMS
control
Tam-Idh1-KI
B
Dcx
control
Tam-Idh1-KI
C
DAPI
YFP
MERGE
control
Tam-Idh1-KI
LV
cc
str
D
DAPI
YFP
MERGE
control
Tam-Idh1-KI
E
DAPI
YFP
MERGE
control
Tam-Idh1-KI
F
Controls
Tam-Idh1-KI
DAPI
YFP
MERGE
a.
b.
c.

Figure S4 related to Figure 5: Ectopic cell migration.

A. Neuroblasts migrating outside the RMS in Tam-Idh1-KI mice compared with controls.
Areas in the inset are magnified. Scale bars 1.5 mm (DAPI) and 100 µm (Dcx and MERGE).

B. Diffuse migration of neuroblasts outside the RMS in the OB of Tam-Idh1-KI mice compared with controls.
Once migrated into the core of the OB, Dcx^+ neuroblasts normally detach from the RMS, and move radially into the granular and glomerular OB layers, where they differentiate into subtypes of $NeuN^+$ interneurons (Ihrie and Alvarez-Buylla, 2011). Here we assess whether *Idh1*^{R132H} may have caused a redirection of SVZ progenitors from the OB to ectopic periventricular regions or may have inhibited normal SVZ neurogenic differentiation. Areas in the inset are magnified. Scale bars 200 µm and 50 µm (magnified).

C. YFP^+ cells emigrating outside the dorsolateral corner of the SVZ in Tam-Idh1-KI reporter (tamoxifen-treated *Nes-Cre*$^{ER(T2)}$*;Idh1*$^{R132H/+}$*;R26R-EYFP*) mice compared with control reporter mice *(Nes-CreER*$^{(T2)}$*;Idh1*$^{+/+}$*;R26R-EYFP*).
LV: lateral ventricle; str: striatum; cc: corpus callosum. Scale bars 100 µm,

D. Migratory stream in the proximal RMS in Tam-Idh1-KI and control YFP reporter mice.
Areas in the inset are magnified. Scale bars 100 µm in A, 400 µm and 200 µm in B, and 400 µm and 50 µm in C.

E. Diffuse migratory stream in the main RMS in Tam-Idh1-KI reporter mice.
Areas in the inset are magnified. Scale bars 100 µm in A., 400 µm and 200 µm in B., and 400 µm and 50 µm in C..

F. YFP^+ cells in the hippocampal dentate gyrus (HDG) and cornu ammonis of Tam-Idh1-KI and control reporter mice.
In addition to the SVZ, the other major adult neurogenic niche is the SGZ of the HDG. Areas in the insets are magnified in (a) and (b). Scale bars 50 µm. The cornu ammonis region of the hippocampus, which is normally non-neurogenic, is indicated by red arrows and shown magnified in (c), scale bars 20 µm.

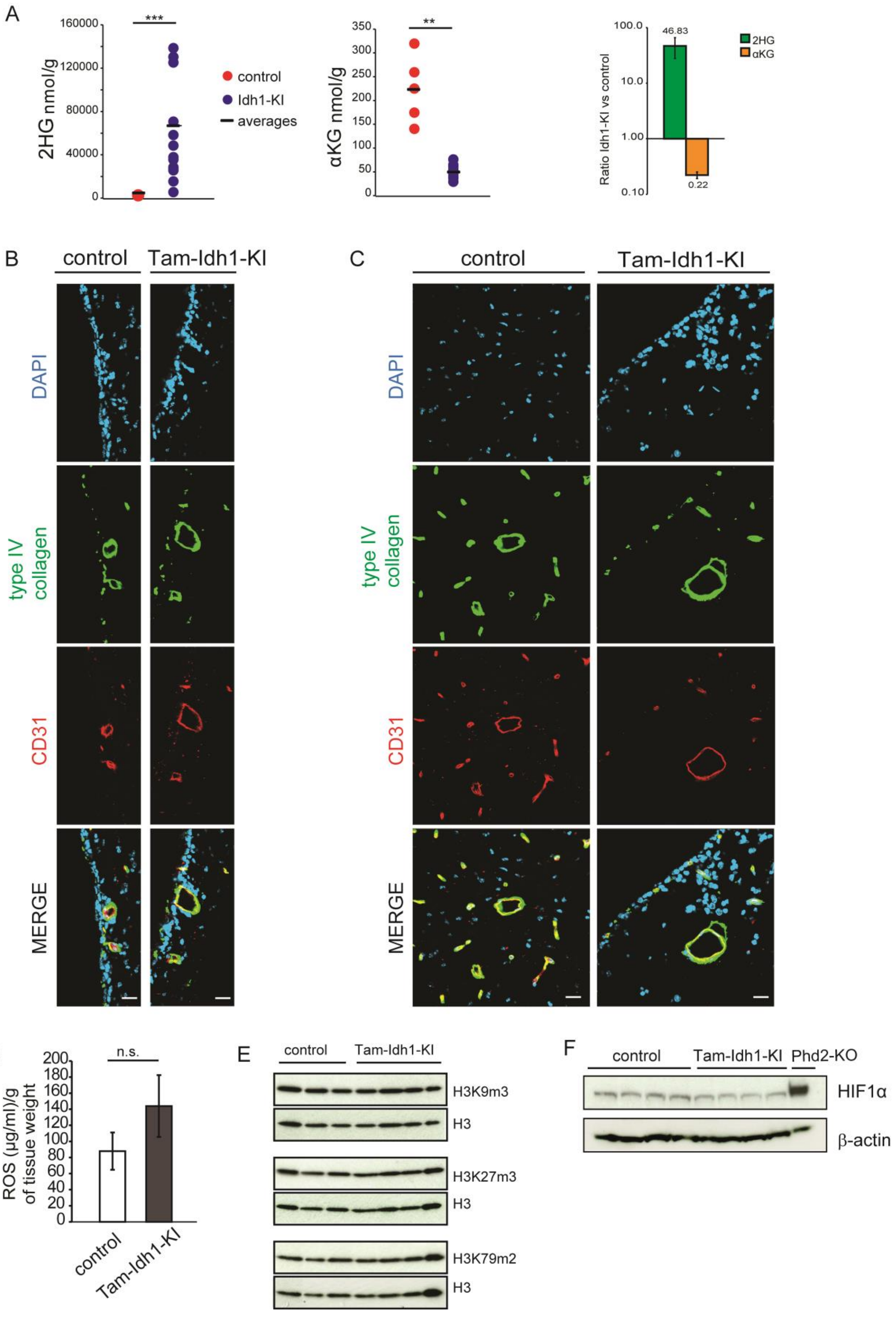

A
2HG nmol/g
160000
120000
80000
40000
0
***
control
Idh1-KI
averages
αKG nmol/g
350
300
250
200
150
100
50
0
**
100.0
10.0
1.00
0.10
Ratio Idh1-KI vs control
46.83
0.22
2HG
αKG
B
control
Tam-Idh1-KI
DAPI
type IV collagen
CD31
MERGE
C
control
Tam-Idh1-KI
DAPI
type IV collagen
CD31
MERGE
D
ROS (μg/ml)/g of tissue weight
200
180
160
140
120
100
80
60
40
20
0
n.s.
control
Tam-Idh1-KI
E
control
Tam-Idh1-KI
H3K9m3
H3
H3K27m3
H3
H3K79m2
H3
F
control
Tam-Idh1-KI
Phd2-KO
HIF1α
β-actin

Figure S5 related to Figure 8: Collagen expression, blood vessel morphology, ROS levels, histone methylation and hypoxia pathway in Tam-Idh1-KIs and controls.

A. Metabolic analysis of brain tissue from Idh1-KI and control embryos.

Whole brains from Idh1-KI (n=16) and control embryos (n=6) at E16-17 were dissociated to a single cell suspension and we performed metabolic profiling using ion chromatography-mass spectrometry (IC-MS). The bar chart shows the data normalized to the average values measured in control brains. Data are presented as mean ± SD. **$p<0.01$, ***$p<0.005$.

B. Type IV collagen expression and blood vessel morphology in the SVZ of adult Tam-Idh1-KI brains compared with controls.

Sections from our mice were immunolabeled with type IV collagen and CD31 antibodies (scale bars 20 µm) to identify blood vessels.

C. Analysis of the adult brain parenchyma for collagen and blood vessel abnormalities in adult Tam-Idh1-KI brains compared with controls.

Details are as for B.

D. ROS levels in the adult brain of adult Tam-Idh1-KI brains and to controls.

Brains from Tam-Idh1-KI (n=3) and control (n=3) mice were analyzed. The difference in levels was not significant ($p>0.05$)

E. Histone methylation in the adult brain.

H3K9m3, H3K27m3 and H3K79m2 were assessed separately using Western blotting. Each blot was probed separately for total H3 as a comparison.

F. Hypoxia pathway in the adult brain.

Western blot for HIF1α was performed on forebrains of Tam-Idh1-KI and control mice. The same blot was probed separately for β-actin as a loading control. Phd2-null fibroblasts were a positive control.

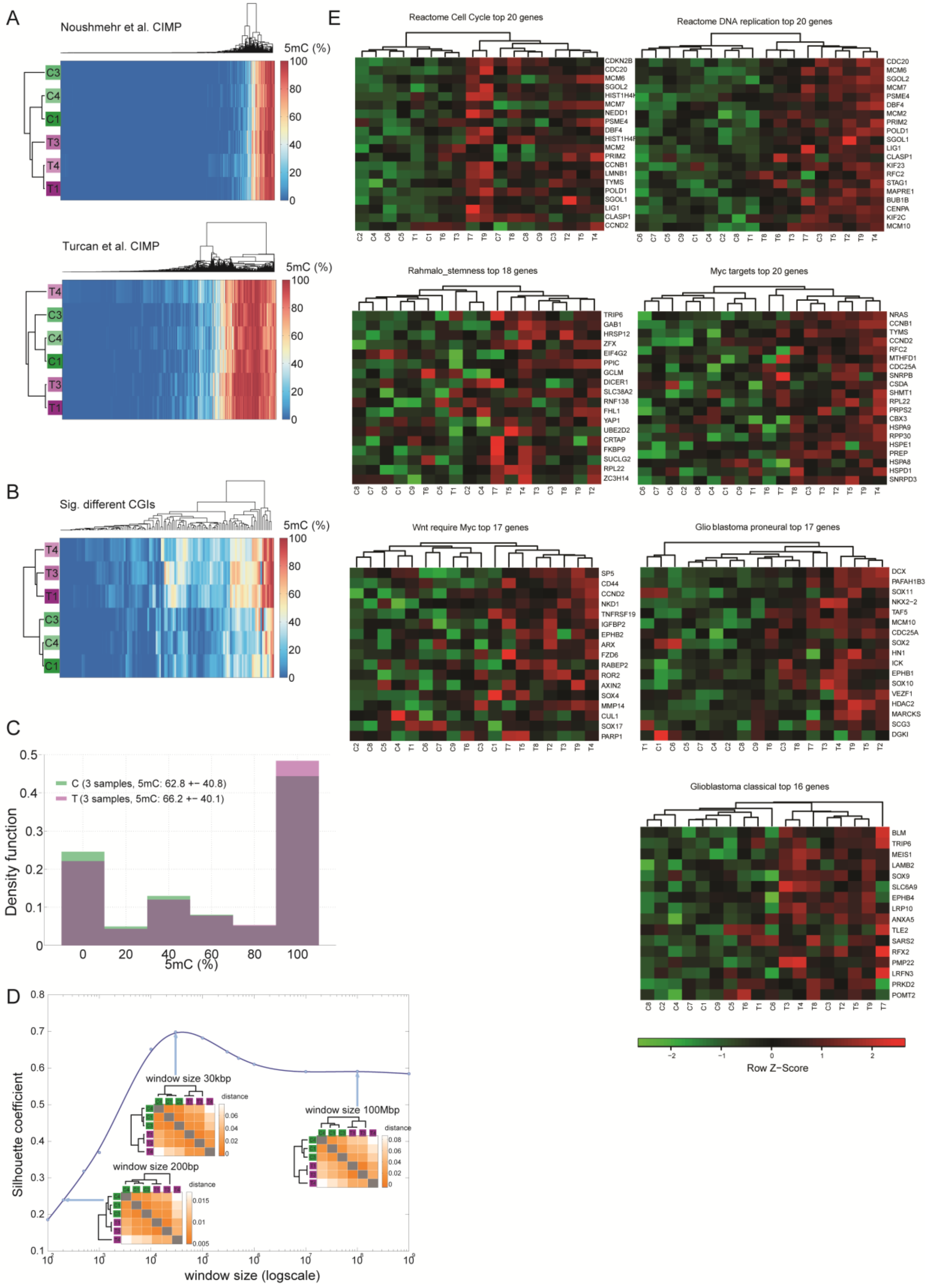

A
Noushmehr et al. CIMP
5mC (%)
Turcan et al. CIMP
B
Sig. different CGIs
C
C (3 samples, 5mC: 62.8 +- 40.8)
T (3 samples, 5mC: 66.2 +- 40.1)
Density function
5mC (%)
D
Silhouette coefficient
window size 30kbp
window size 100Mbp
window size 200bp
distance
window size (logscale)
E
Reactome Cell Cycle top 20 genes
Reactome DNA replication top 20 genes
Rahmalo_stemness top 18 genes
Myc targets top 20 genes
Wnt require Myc top 17 genes
Glio blastoma proneural top 17 genes
Glioblastoma classical top 16 genes
Row Z-Score

Figure S6 related to Figure 8: Further details of the OxBSseq for 5mC and of gene expression analysis.

A. Hierarchical clustering of methylated CpGs for genes postulated as targeted by the CpG island methylator phenotype in human glioma by other groups (Noushmehr et al., 2010; Turcan et al., 2012).

B. Hierarchical clustering of significantly methylated CpGs in our data.
Significance was assessed using Fisher's exact test (after Bonferroni correction of p values).

C. Density plot of methylation level at CpGs genome-wide in our data (proportion of Cs methylated) in Tam-Idh1-KI (T) animals and control (C) animals. The large proportion of completely methylated and completely unmethylated sites is likely to represent both a true biological phenomenon and an effect of some single or low coverage sites.

D. Silhouette analysis to determine the methylation window size that discriminates most between Tam-Idh1-KI and control mice.

E. Heat maps of the genes showing largest differences in mRNA expression for selected pathways identified using GSEA analysis.
The pathways represented were chosen as strong candidates for involvement in gliomagenesis that were significantly different, after correction for multiple testing, between Tam-Idh1-KI (T1-9) and wildtype mice (C1-9). Unsupervised hierarchical clustering was used to group mice, with the purpose of assessing a tendency for Tam-Idh1-KI and wildtype mice to cluster separately.

Table S1 related to Figure 8. Gene set enrichment analysis of mRNA expression profiling in Tam-Idh1-KI and control SVZs. Provided as an Excel file.

SIZE: no. of genes in set; ES: enrichment score; NES: normalized enrichment score; NOM p val: nominal p value; FDR q val: Benjamini-Hochberg false discovery rate; FWER p val: family-wise error rate p value; RANK AT MAX: position in the ranked list at which the maximum enrichment score occurred; LEADING EDGE: results are explained in detail at http://software.broadinstitute.org/gsea/doc/GSEAUserGuideFrame.html/.

Gene sets concordant with Tam-Idh1-KI at FDR<0.05 or discordant at FDR<0.01 are shown. Sets with putative importance for gliomagenesis are in bold. These include cell cycle components, DNA replication, telomere maintenance, Myc targets, Wnt targets, Tlx targets, stem cell behavior and human glioblastomas.

SUPPLEMENTAL EXPERIMENTAL PROCEDURES

Generation of mice

We designed a targeting construct for replacement of the endogenous *Idh1* gene in ES cells, comprising a 5' homology arm (~4.0 kb), a conditional knock-in region (~2.5 kb), a 3' homology arm (~3.9 kb) that included the R132H mutation, and a DTA expression cassette. The knock-in region comprised a 5' loxP site, a wildtype mini-gene (exons 3-9 and 3'UTR), an SV40 polyA signal, a Neo^R cassette flanked by Frt sites, and a 3' LoxP site (Figure 1). These fragments were sequentially cloned into the LoxFtNwCD vector and were confirmed by restriction digestion and end-sequencing. NotI was used to linearize the final vector prior to electroporation into C57BL/6 ES cells, which were selected with 200 µg/ml G418. The construct was incorporated by homologous recombination into C57BL/6N ES cells. After PCR-based screening and R132H mutation sequencing, 3 clones carrying the R132H mutation were expanded for further analysis. After additional Southern and PCR/sequencing confirmation analysis two clones were confirmed to be correctly targeted. These cells were injected into a C57BL/6J-tyr albino blastocyst donor strain, and, after confirming germline transmission of the mutant allele, we maintained the resulting *Idh1*$^{fl(R132H)/+}$ animals on the C57BL/6 background. We subsequently used Cre-mediated recombination to remove the Neo^R cassette and knock-in the R132H allele *in vivo*. Other mice were provided by collaborators or from public sources. All mouse procedures were carried out in accordance to Home Office UK regulations and the Animals (Scientific Procedures) Act 1986. All mice were housed at the animal unit at Functional Genomics Facility, Wellcome Trust Centre for Human Genetics, Oxford University.

Excision of the Neo^R cassette

In order to remove the Frt-flanked Neo^R cassette, we crossed *Idh1*$^{fl(R132H)/+}$ animals with homozygous FLPe mice. These crosses, however, repeatedly failed to produce *Idh1*-mutant offspring, and FLPe x *Idh1*$^{fl(R132H)/fl(R132H)}$ crosses produced no offspring at all. Since construct design meant that Cre-mediated recombination would both remove the Neo^R cassette and lead to knock-in of the R132H allele, we decided to retain the Neo^R until the time of mutation knock-in using constitutive or tamoxifen-inducible Nestin-Cre.

Tamoxifen treatment and DNA labelling

To create the Tam-Idh1-KO model, *Nes-Cre*$^{ER(T2)}$;*Idh1*$^{fl(R132H)/+}$ animals received intraperitoneal (i.p.) tamoxifen injections at 5 or 6 weeks of age. For labelling studies, 5-Bromo-2′-deoxyuridine (BrdU) (Sigma Aldrich) and 5-ethynyl-2′-deoxyuridine (EdU) (Life Technologies) were dissolved in sterile normal saline (10 mg/ml) and injected i.p. 18-28 weeks after tamoxifen (3 daily single injections of BrdU at 50 mg/kg and 13 days later, 2 hours before sacrifice, one dose of EdU at 50 mg/kg).

Crosses with reporter R26R-EYFP mice

To verify that recombination of the *Idh1* conditional allele had been targeted in cells that specifically express *Nestin*, we crossed *Nes-Cre*$^{ER(T2)}$;*Idh1*$^{fl(R132H)/+}$ and *Nes-Cre*$^{ER(T2)}$;*Idh1*$^{+/+}$ animals with a R26-lox-STOP-lox-EYFP (R26R-EYFP) reporter mouse line. These transgenic mice have a *loxP*-flanked STOP sequence followed by the Enhanced Yellow Fluorescent Protein gene (EYFP) inserted into the *Gt(ROSA)26Sor* locus (Srinivas et al., 2001). When bred to mice expressing Cre recombinase, the STOP sequence is deleted and EYFP expression is observed in the *Cre*-expressing tissue(s) of the mutant offspring.

Mouse genotyping

The mouse lines used in the study were genotyped from ear clipping or brain DNA using the PCR primers shown.

Purpose	Primer ID	Forward	Reverse	Size (bp)
Presence of sequence between Frt sites	Flpe	ACGGAACAGCAATCAAGAGAGCCA	TCGATCCTACCCCTTGCGCTAAA	450
Presence of NeoR cassette	Neo	AGGATCTCCTGTCATCTCACC	AAGAACTCGTCAAGAAGGCGA	493
Idh1 wildtype allele	Idh1-KI	GGTTGGACTTGGCTTTAATTTG	ATTGGTGGCATCACGATTCT	393
Idh1 "floxed" allele	Idh1-KI	GGTTGGACTTGGCTTTAATTTG	ATTGGTGGCATCACGATTCT	529
Idh1 Cre-recombined allele	Idh1-KI	GGTTGGACTTGGCTTTAATTTG	ATTGGTGGCATCACGATTCT	551
Cre recombinase	Cre1	TTACCGGTCGATGCAACGAG	CCACCGTCAGTACGTGAGAT	500
Cre recombinase	Cre2	GCGGTCTGGCAGTAAAAACTATC	GTGAAACAGCATTGCTGTCACTT	100
Internal positive control	IC	CTAGGCCACAGAATTGAAAGATCT	GTAGGTGGAAATTCTAGCATCATCC	324
Presence of YFP transgene at R26 locus	YFP	AAAGTCGCTCTGAGTTGTTAT	AAGACCGCGAAGAGTTTGTC	320
Presence of wild type allele at R26 locus	YFP	AAAGTCGCTCTGAGTTGTTAT	GGAGCGGGAGAAATGGATATG	600

Mouse behavior

The open field test (OFT) was used to investigate mutant mouse behavior. A new environment causes mice to move and explore, but anxiety and fear cause mice to crouch and freeze. In general, mice that are inactive in an OFT are assumed to have anxiety and fear, and mice that are active are assumed to have less apprehension and fear. The OF used in this study was a rectangular enclosure, marked inside with 16 square areas, and with surrounding walls to prevent escape. Tam-Idh1-KI and control mice of same age were placed individually into the OF. Numbers of squares crossed and rears made were measured during a 5 minute time period. For the test we used 4 Tam-Idh1-KI mice and 4 controls mice aged between 3 to 5 months.

Post mortem examination

The following organs were analyzed in Tam-Idh1-KI and aged-matched control mice: brain, kidneys, liver, heart, lung, spleen, esophagus, lymph nodes, gall bladder, pancreas, small intestine and large intestine and blood.

Ex vivo magnetic resonance imaging

5 and 12 month-old Tam-Idh1-KI and control mice were sacrificed under terminal anesthesia with an i.p. overdose of pentobarbitone, followed by exsanguination using intracardial perfusion with a solution 0.9% of NaCl, and then a fixative solution of 4% paraformaldehyde (PFA). Heparin at a concentration of 10 IU/ml was added to the saline solution (hepsal). To allow imaging of the brains, gadolinium-DTPA (Gd-DTPA) contrast at a concentration of 27.93 mg/ml (Bracco Diagnostics) was added to both saline and PFA solutions. Briefly, after injection with 100 µL of 200 mg/ml of pentobarbitone (Pentoject, Animalcare Limited). The thorax was then opened and the heart located. The right atrium was cut from the vena cava. A 21 gauge butterfly needle was inserted into the left ventricle and 20 ml of hepsal was injected through the butterfly to wash the blood out of the mouse. Subsequently 20 ml of the 4% PFA/contrast mix was injected to fix the mouse tissues. The mouse's head was then dissected from the body, the fur and skin were removed, and the jaw dissected. The sample, comprising the skull and the brain, was then incubated in 4% PFA solution at 4C° overnight. The following day, one sample dissected from Tam-Idh1-KI and one sample dissected from control mice were placed into 20 ml tubes (in opposite orientation one to each other), embedded entirely in 1% agarose, and left to set. MRI was performed on a horizontal bore 9.4T magnet with a Varian DirectDrive™ (Agilent Technologies, Santa Clara, CA, USA). 20 ml tubes containing one Tam-Idh1-KI sample and one control sample were positioned in a quadrature birdcage coil (2.0 cm internal diameter; RAPID MR International). In brief, T_1-weighted 3D multi-echo multi slices (MEMS) dataset was acquired with the following parameters: TR=500ms TE=21ms, matrix size of 256 x 256 x 256 and field of view of 2.25cm x 2.25cm x 2.25 cm. Only one 3D echo dataset was shown. To quantify ventricle volumes, 3D dataset were converted into .nifti files and segmented using itk-SNAP version 3.0 (http://www.itksnap.org/) (Yushkevich et al., 2006).

SVZ microdissection and cultures

After having killed the mice using terminal anesthesia (with an i.p. overdose of pentobarbitone when working with adult mice, or with hypothermia when working with pups) followed by decapitation, brains were extracted, and sectioned in a Zivic mouse brain slicer. 500 µm brain sections were placed in Hanks balanced salt solution and the SVZ carefully microdissected under a Leica MZ12 dissecting microscope. Adult SVZ tissues were snap-frozen in liquid nitrogen and kept at -80°C for DNA, RNA or protein extractions. Primary cultures of SVZ cells were performed from brains of C57BL6 pups at P4. SVZ tissues were dissected and dissociated with accutase (Sigma) at 37ºC. SVZ cells were then cultured in Neurobasal medium (Gibco, cat#10888-022) containing B27 (Gibco, cat#17504) glutamax (Gibco 35050-038), Pen Strep (Gibco 15070-063), EGF (20 ng/mL, Sigma) and FGF-2 (20 ng/mL, R&D).

Fluorescent immunohistochemistry, confocal microscopy and image quantification

Mice were killed as for SVZ microdissection, transcardially perfused with normal saline and then 4% paraformaldehyde (PFA). Brains were extracted, post-fixed in 4% PFA, cryoprotected in 30% sucrose and frozen. 30 µm coronal brain sections were cut on a sliding microtome (Leica), and kept in cryoprotectant at -20°C. Sections were stained using standard free-floating immunohistochemistry. Images were acquired on Zeiss 510 Metahead and Leica SP8 SMD X confocal microscopes. Around 6 brain sections per mouse were used in each staining for three regions (RMS: Bregma 2.34mm to 1.78mm; SVZ: Bregma 1.70mm to -0.82mm, Hippocampus: Bregma -0.94 to -2.30mm). At least 3 z-stacks of 15-20 optical slices (1-2 µm intervals) or 8 different single optical planes per section were acquired. For each immunofluorescence, numbers of immune-positive cells were counted in z-stacks. Quantification of immune-positive cells in single optical planes were represented as specified in the figure legends. All quantifications were done by an observer blinded to experimental conditions. All the images were quantified on Volocity 6.3 (Improvision).

Antibodies

For immunofluorescence, the following primary antibodies were used: rabbit α-type IV collagen (Rockland, cat#600-401-106-0.1), rabbit α-GFP (Abcam, cat#6556), rat α-BrdU (Novus Biologicals, cat#nb500-169), rat α-CD31 (BD Pharmingen, cat#557355), goat α-Dcx (Santa Cruz Biotechnology, cat#sc8066), rabbit α-Dcx (Abcam, cat#ab18723), chicken α-GFAP (Abcam, cat#ab4674), rabbit α-Ki67 (Abcam, cat#ab16667), mouse α-NeuN (Milliphore, cat#MAB377), rabbit α-Caspase 3 (Cell Signaling, cat#9664), rabbit α-Olig2 (Millipore, cat#ab9610), mouse α-S100β (Sigma, cat#s2657), goat α-MBP (Santa Cruz Biotechnology, cat#sc13914), mouse α-nestin (Millipore, cat#mab353), rat α-PDGFRα (CD140a) (BD Biosciences, cat# 558774). Secondary antibodies conjugated to Alexa Fluor 488, 568, 594, or 647 (Invitrogen, Sigma and Jackson Laboratory) were used as appropriate. For EdU detection, the Click-iT® EdU detection kit (Invitrogen) was used and the manufacturer's protocol was adapted to Free-Floating IHC.

Non-fluorescent immunohistochemistry

Tissue samples were fixed in 10% buffered formalin or 4% paraformaldehyde (PFA) at 4°C and embedded in paraffin. Sections (5 µm) were stained with Harris hematoxylin and eosin according to standard protocols. EnVision™+ Kits (Dako) was used for immunostaining, accordingly to manufacturer's instructions. In brief, sections (5 µm) were de-waxed in xylene and rehydrated through graded alcohols to water. For antigen retrieval, sections were pressure cooked in 1x antigen retrieval solution for 10 min and allowed to cool down. Endogenous peroxidase was blocked for 5 min. Slides were then incubated with primary antibody for 1 h at room temperature. HRP labeled polymer, conjugated with the appropriate secondary antibodies, was then applied for 30 min at room temperature. Staining was completed by a 2–10 minute incubation with 3-amino-9-ethylcarbazole (AEC)+ substrate chromogen and the development of the color reaction at the antigen site was monitored microscopically. Slides were counterstained with hematoxylin, dehydrated, cleared and then mounted. Microscopic observations and images were acquired using a Nikon wide-field TE2000U Microscope. The following primary antibodies were used: rabbit α-Ki-67 (Cell Signaling, cat#12202).

Western blotting

Total protein extraction from cells was performed according to standard methods. Tissue culture medium was removed, and cells washed twice with PBS. SDS-containing tissue lysis buffer was then added, and cells were detached using a Corning cell scraper (Sigma-Aldrich), transferred to Eppendorf tubes and incubated in SDS at 95°C for 5 min. Extracts were then homogenized by ultrasound using an acoustic transducer, and centrifuged at 14,000 rpm for 10 min. The protein-containing supernatant was removed and protein concentration was established. Total protein extraction from mouse tissue was performed using urea tissue lysis buffer, made up of 7 M urea, 10% glycerol, 10 mM Tris-HCl (pH 6.8), 1% SDS, 5mM DTT, and a cOmplete Mini Protease Inhibitor Tablet (Roche). 200 µL of buffer was added to tissue which was then homogenized through a 21 gauge needle. This homogenate was then sonicated for 10 sec x3, and then mixed by rotation for 30 min at 4°C, before being clarified by centrifugation at 13000 rpm for 15 minutes at 4°C. Protein concentrations were determined using the Pierce BCA Protein Assay Kit (Thermo Scientific). Western blots were performed with the NuPAGE Gel system (Invitrogen) according to the manufacturer's protocol. In brief, denatured lysates were loaded onto a gel to run at 150V for 2 h. The gels were then transferred onto an Immobilon-P PVDF transfer membrane (Millipore) in a semi-dry tank, and blocked by incubating for 1 h at room temperature in 10% bovine serum albumin (BSA) or 10% milk. The membranes were then incubated overnight in the appropriate primary antibody in 5% BSA, or 5% milk. After washing, the membranes were incubated in the appropriate peroxidase-conjugated secondary antibody for 1 h at room temperature. After further washes, the blots were incubated in ECL reagents (GE healthcare) and bound antibodies were detected using enhanced chemiluminescence with developing chemiluminescence film (GE Healthcare) with a developer. The primary antibodies utilized were the following: rabbit α-HIF1 (Cayman Chemical Company, cat#10006421), mouse β-Actin (Santa Cruz Biotechnology, cat#sc-47778), rabbit α-IDH1 (Cell signaling technology, cat#3997), mouse α-IDH1-R132H (Millipore, cat#MABC171), rabbit α-H3K9me3 (Abcam, cat#ab8898), rabbit α-H3K79me2 (Abcam, cat#ab3594), rabbit α-H3 (Abcam, cat#ab131711), and rabbit α-H3K27me3 (Millipore, cat#07-449).

RNA extraction and RT-PCR

RNA was purified using the RNeasy microkit (Qiagen) according to the manufacturer's instructions. When required, complementary DNA was reverse transcribed *in vitro* using the High Capacity cDNA Reverse Transcription Kit (Applied Biosystems). cDNA generated from SVZ dissected cells, was pre-amplified before qRT-PCR, using The TaqMan PreAmp (Applied Biosystems) kit and following manufacturer's instructions. Absolute quantification qRT-PCR was performed on the ABI 7900HT cycler (Applied Biosystems) with β-actin serving as an endogenous control.

A list of TaqMan Gene Expression assays (Applied Biosystems) used is available on request. Each target gene's expression was evaluated using a relative quantification approach ($2^{-\Delta\Delta CT}$ method).

DNA extraction
DNA was extracted using the QiaAmp Micro kit (Qiagen) following the manufacturer's instructions.

Nucleic acid sequencing
Sequencing of gDNA and cDNA was carried out using the 2× Big Dye Terminator v3.1 reagent (Applied Biosystems). Unincorporated dye terminators were removed with the DyeEx 2.0 Spin kit (Qiagen) and the purified products were run on the ABI 3730 DNA analyzer (Applied Biosystems). To sequence gDNA, the following primers were have been used: Idh1_ex3_F TCAAGTTGAAACAAATGTGGAAA and Idh1_IN3_R GGGTGTAGATGCCCAAAGAA. To sequence cDNA, the following primers were used: Idh1_ex3_F TCAAGTTGAAACAAATGTGGAAA and Idh1_ex4_ex5_R GCAACACCACCACCTTCTTC. The short isoform of *Idh1*, missing exons 1 and 2, was identified by sequencing of cDNAs amplified using the following primers: Idh1_fw_geno_IN2_a CAGGCTAAGCACCCATGTTT and Idh1_ex4_ex5_R GCAACACCACCACCTTCTTC. To avoid DNA contamination, RNA samples were DNAse treated and the resulting cDNA amplified with PCR primers spanning exons. All PCR conditions are available upon request.

PCR amplification of alternative “leaky” *Idh1* transcript
The *Idh1* transcript lacking exons 1 and 2, was amplified from cDNA by end-point RT-PCR (amplicon size 562bp) and sequenced using the following primers: Idh1_fw_geno_IN2_a CAGGCTAAGCACCCATGTTT and Idh1_ex4_ex5_R GCAACACCACCACCTTCTTC, the former located in the canonical intron 2, and the latter spanning canonical exons 4 and 5. Further details are available on request.

ReNcell CX immortalized cell line
The ReNcell CX immortalized cell line (Millipore Cat. No. SCC007) was originally derived from 14-week human cortical brain tissue and were cultured in ReNcell NSC Maintenance Medium (Millipore Cat. No. SCM005) according to the supplier’s instructions.

Neurosphere assays
For unknown reasons, possibly relating to the central roles of the IDHs in metabolism, culture of *IDH1*-mutant human tumors has proved difficult in general and our attempts to culture primary *Idh1*-mutant neurospheres from the SVZ of Tam-Idh1-KI mice were similarly unsuccessful. We therefore adopted an alternative strategy by using lentiviral vectors to express the R132H mutation in neuronal stem/progenitor cells (NPCs) from the cerebral cortex of the human fetal brain (ReNcell CX immortalized cells) or dissected from the SVZ of a wildtype BL6 mouse of P4 age. For human neurosphere assays, ReNcell CXs were transduced with $IDH1^{R132H}$, with untransduced cells as controls; GFP-expressing constructs were used to ensure transduction efficiency (estimated 60%). For mouse neurosphere assays, NSC/NPC cells were transduced with $IDH1^{R132H}$, with cells transduced with wildtype IDH1 as controls; GFP-expressing constructs were again used to ensure transduction efficiency (estimated 60%). For assessment of neurosphere formation, cells were plated at low density (10 cells/µL, 2000 cells/cm^2) or at high density (100 cells/µL, 20000 cells/ cm^2) in non-tissue culture treated 96 or 24 well plates. The number of neurospheres was counted 7-10 days after plating for cells plated at low density and 4 days for cells plated at high density. Microscopic images were acquired using a Nikon wide-field TE2000U Microscope and analyzed using ImageJ 1.47v.

Transwell cell motility assay
The motility assay was performed in polycarbonate Transwell filter chambers of 8.0-µm pore size and 6.5-mm diameter (Costar, Cambridge, MA). The upper and bottom side of each porous polycarbonate membrane were coated with laminin at 20 mg/mL overnight. Filters were then washed with PBS and plated with cells ($2.5x10^4$/well; 2 wells for each experimental condition) on the upper side of the filter. The lower chamber was filled with ReNcell NSC Maintenance Medium containing 20 ng/mL FGF-2 and 20 ng/mL EGF (basal condition) or medium containing twice as much FGF (40 ng/mL) or 5% FCS. After 24h, cells on the upper side of the filters were mechanically removed. The cells that had migrated to the lower side were fixed with 11% glutaraldehyde in PBS and stained with 0.1% crystal violet in 20% methanol. In the assay, cells expressing $IDH1^{R132H}$ were compared to untransduced controls. Microscopic images of the cells that had passed through the filters were acquired using a Nikon wide-field TE2000U microscope and counted using NIS-Element microscope imaging software 4.0.

Molecular cloning and cell transduction with lentiviral vectors

Human *IDH1* cDNA was amplified by PCR using primers which added 1X-FLAG tag to the 3' end of the sequence. This was cloned as ClaI-NheI fragment into the pCC.sin.36.MCS.PPTWpre.CMV.tTA-S2tet lentiviral transfer vector. Subsequently a recombinant PCR-based approach, using *IDH1* cDNA as a template was applied to construct the *IDH1* R132H mutant, which was cloned into the same lentiviral transfer vector as a ClaI-BmgBI fragment, by substituting the *IDH1* cassette with the *IDH1*-R132H mutant cassette. As a control, cells were transduced with a pCC.sin.36.eGFP.PPT.Wpre.CMV.tTA-s2tet, encoding eGFP and pCC.sin.36.IDH1WT.PPTWpre.CMV.tTA-S2tet, encoding the wild type sequence of human *IDH1*. PCR primers for the above are available upon request. Vector stocks were produced by transient transfection of 293T cells. Serial dilutions of freshly harvested conditioned medium were used to infect 10^5 cells in a six-well plate, in the presence of Polybrene (8 µg/ml). The viral p24 antigen concentration was measured by an HIV-1 p24 core profile enzyme-linked immunosorbent assay ELISA assay (Lenti-X p24 rapid titer kit, Clontech) to determine the number of infective particles before transduction and to demonstrate that transduced cells did not produce viral particles after transduction.

ROS measurements

Reactive oxygen species in brain samples (Tam-Idh1-KI n=3 controls n=3, aged between 4 and 6 months) were quantified using the Mouse Reactive Oxygen Species ELISA kit (BG Bluegene), following the manufacturer's instructions.

Quantitation of genomic 5hmC and 5mC by HPLC

Purified genomic DNA (1-10 µg) was extracted from a forebrains dissected from Tam-Idh1-KI n=13 and controls n=8 (aged between 4 to 8 months) and then incubated with 200 U of RNase A/T1 Mix (#EN0551, Thermo Scientific) in 1x NEB Buffer 2 for 2 hours at 37°C. The DNA was extracted using Phenol:Chloroform:Isoamyl Alcohol 25:24:1 (#P3803, Sigma) followed by ethanol precipitation. The recovered DNA was hydrolyzed as described (Kriaucionis and Heintz, 2009). Proteins were removed by 20 min centrifugation at 14000 g using Amicon Ultra-0.5 (3 kDa) column (#UFC5003BK, EMD Millipore). The flow-through was lyophilized and re-suspended in 50 µl Buffer A (100 mM Ammonium acetate pH 6). 40 µl of the solution was injected into the Agilent 1290 Infinity Series HPLC system. The flow rate was set at 0.400 mL/min. Nucleosides were separated using Eclipse Plus C18, 2.1x150 mm, 1.8 µm column (Agilent) at 40 °C. The run was performed using 1.8% to 30% linear gradient of 40% Acetonitrile. Acquired data were analyzed using Agilent 1290 Infinity Series software. Statistical analysis was performed using ANOVA with age and sex as an additional explanatory variables.

Quantification of genomic 5mC by sequencing

Genomic DNA (at least 200 ng) was extracted from SVZ cells dissected from Tam-Idh1-KI n=3 and controls n=3 (aged between 9 to 11 months). DNA was then sonicated to an average size of 500 bp using the Covaris S220 instrument (Covaris). Oxidative bisulfite treatment of DNA was performed using the TrueMethyl24 Kit (Cambridge Epigenetix) according to the manufacturer's instructions. 10 µL of converted material were used for sequencing library preparation using the Accel-NGS Methyl-Seq DNA Library Kit (cat#DL-ILMMS-12, Swift Bioscience) according to manufacturer's instructions with some minor adjustments. Briefly, the libraries were amplified using 10 PCR cycles with barcoded primers from the Methyl-Seq Dual Indexing Kit (cat#DI-ILMMS-12, Swift Bioscience). Amplicons were selected for an average library size of 350 bp using SPRI Select Beads (cat#B23317, Beckman Coulter). Sequencing libraries were sequenced using three lanes of the HiSeq2500 Rapid Mode. After removal of low quality bases, overlapping read pairs and duplicate reads, genome sequencing coverage of CpG cytosines was a median of 2.4x. This was sufficient for global measures of methylation changes (across CpG islands, genes or other features), but did not permit analysis at the level of specific genes or regions. To determine whether observed differences were focal or evenly dispersed, we calculated the density of 5mC in genomic bins of increasing size (ranging from 200 bp to 100 Mbp) and performed unsupervised clustering.

Metabolic analysis of embryonic brains

Ion chromatography-mass spectrometry (IC-MS) was used for analysis. Metabolites were extracted using ice cold 80% aqueous methanol. Briefly, frozen mouse tissues were weighed, added to a 6-fold volume of ice-cold 80% methanol and disrupted to obtain a single cell suspension, with a tissue homogenizer (Polytron PT1200E Homogenizer, Kinematica). The extracts were then clarified by centrifugation at 14,800 rpm for 10 min at 4 °C (this step was repeated twice), and then dried in a SpeedVac and subsequently stored at -80°C. On the day of analysis the dried extracts were re-constituted in 60 µL of Milli-Q water. Organic acids and sugar phosphates where analyzed using a Thermo Scientific ICS-5000+ ion chromatography system coupled directly to a Q Exactive HF Hybrid

Quadrupole-Orbitrap mass spectrometer using a HESI II Electrospray ionization source (Thermo Scientific, San Jose, CA). The ICS-5000+ HPLC system incorporated an electrolytic anion generator (KOH) which was programmed to produce the desired hydroxide ion gradient. An inline electrolytic suppressor removed the OH- ions and cations from the post-column eluent prior to MS analysis (Thermo Scientific Dionex AERS 500). A 10 µL partial loop injection was used for all analyses and the separation was performed using a Thermo Scientific Dionex IonPac AS11-HC 2 × 250 mm, 4 µm particle size column with a Dionex Ionpac AG11-HC 4 µm 2x50 guard column inline. The IC flow rate was 0.250 mL/min. The total run time was 37 minutes and the hydroxide ion gradient comprised the following: 0 min, 0 mM; 1min, 0 mM; 15 min, 60 mM; 25 min, 100 mM; 30 min, 100 mM; 30.1min, 0 mM; 37 min, 0 mM. Analysis was performed in negative ion mode using a scan range from 80-900 and resolution set to 70,000. The tune file source parameters were set as follows: Sheath gas flow 60; Aux gas flow 20; Spray voltage 3.6; Capillary temperature 320; S-lens RF value 70; Heater temperature 450. AGC target was set to 1e6 and the Max IT value was 250 ms. The column temperature was kept at 30°C throughout the experiment. Data were acquired in the full scan, continuum mode. Peak retention times were identified from the injection of authentic standards and peaks from unknown samples were identified using a combination of accurate mass analysis (<2 ppm) and retention time using Thermo Scientific Quanbrowser software (Thermo Fisher Scientific, Hemel Hempstead, UK).

Metabolic analysis of adult brains

All adult brain samples were analyzed with IC-MS as above, and additionally using capillary electrophoresis time-of-flight mass spectrometry (CE-TOFMS) or (for α-ketoglutarate) the higher sensitivity method of liquid chromatography time-of-flight mass spectrometry (LC-TOF-MS) method. Forebrain extractions were used, because the quantity of material obtained from the SVZ was too small for optimal analysis. To prepare samples, frozen tissues were completely homogenized by a cell disrupter (Shake Master NEO; Bio Medical Science) at 4°C, after adding 500 µL of methanol containing internal standards [20 µM each of methionine sulfone and 2-(N-morpholino)ethanesulfonic acid (MES)]. The homogenate was then mixed with 200 µL of Milli-Q water and 500 µL of chloroform and centrifuged at 9100 *g* for 3 h at 4°C. Subsequently, the aqueous solution was centrifugally filtered through a 5-kDa cut-off filter (Millipore) to remove proteins. The filtrate was centrifugally concentrated and dissolved in 50 µL Milli-Q water containing reference compounds (200 µM each of 3-aminopyrrolidine and trimesate). Prior to CE-TOFMS analysis, the sample solution for anion was diluted 10 times and that for cation was diluted five times with Milli-Q water, respectively. For the α-ketoglutarate analysis, the sample solution was not diluted. The concentration of each metabolite was calculated as described previously (Soga et al., 2009). In all CE-TOFMS experiments, the Agilent CE capillary electrophoresis system (Agilent Technologies) was used, and in LC-TOFMS experiments, the Agilent G3250AA LC/MSD TOF system was used. For anionic metabolite profiling, the original Agilent stainless steel ESI needle was replaced with the Agilent G7100-60041 platinum needle (Soga et al., 2009). IC-MS and TOFMS data were found to be concordant in direction of effect. Statistical analysis was performed using ANOVA with source of the data, age and sex as additional explanatory variables.

Gene expression microarray analysis

RNA was extracted from SVZ cells of 9 Tam-Idh1-KI and 9 control mice (aged between 5 to 9 months old), and hybridized to Illumina Mouse WG6-v2 Expression BeadChip microarrays following the manufacturer's protocols. Raw data were imported into the R statistical software for further processing and analysis using BioConductor packages (Gentleman et al., 2004). Raw signal intensities were background corrected (using array-specific measures of background intensity based on negative control probes), prior to being transformed and normalized using the 'vsn' package (Huber et al., 2002). A range of quality control checks were made and all samples produced high quality data. The dataset was then filtered to only include probes detected above background levels (detection score >0.95) in at least 3 samples, resulting in a final dataset of 25580 probes. Statistical analysis to identify differential gene expression between the Tam-Idh1-KI and control groups was performed with the Linear Models for Microarray Analysis (limma) package (Smyth et al., 2005). Raw p values were corrected for multiple testing using the false discovery rate (FDR) controlling procedure of Benjamini and Hochberg (Benjamini and Hochberg, 1995). At 5% FDR, 33 probes showed significant changes in their expression level between experimental groups. Results were annotated with gene information using the relevant BioConductor annotation package (https://bioconductor.org/packages/release/data/annotation/html/illuminaHumanv4.db.html). Gene set enrichment analysis (GSEA) was performed using the 4725 gene set from the BROAD Institute with the molecular signature C2 (http://www.broadinstitute.org/gsea/msigdb/collections.jsp) using Kolmogorov-Smirnov statistics and gene shuffling permutations as described (Subramanian et al., 2005). Genes were ranked by the moderated t-statistics from the limma package. If multiple probes were present for a gene, the probe with the most significant p value for differential expression was selected. We used gene shuffling with 1,000 permutations to compute the p value for the enrichment

score. In Figure 8, illustrative plots are shown for the following gene sets: Cell cycle and DNA replication using Reactome gene sets; Stemness (Ramalho-Santos et al., 2002); Genes enriched in glioblastoma proneural (Verhaak et al., 2010); Myc target genes (Zeller et al., 2003); and Wnt pathway targets requiring Myc (Sansom et al., 2007)).

SUPPLEMENTAL REFERENCES

Benjamini, Y., and Hochberg, Y. (1995). Controlling the false discovery rate: a practical and powerful approach to multiple testing. J. Roy. Stats. Soc. Series B *57*: 289–300.

Curtis, M. A., Kam, M., Nannmark, U., Anderson, M. F., Axell, M. Z., Wikkelso, C., HoltÂs, S., van Roon-Mom, W. M., Bjork-Eriksson, T., Nordborg, C., et al. (2007). Human neuroblasts migrate to the olfactory bulb via a lateral ventricular extension. Science *315*, 1243-1249.

Doetsch, F., Caille, I., Lim, D. A., Garcia-Verdugo, J. M., and Alvarez-Buylla, A. (1999). Subventricular zone astrocytes are neural stem cells in the adult mammalian brain. Cell *97*, 703-716.

Ernst, A., Alkass, K., Bernard, S., Salehpour, M., Perl, S., Tisdale, J., Possnert, G., Druid, H., and Frisen, J. (2014). Neurogenesis in the striatum of the adult human brain. Cell *156*, 1072-1083.

Gentleman, R. C., Carey, V. J., Bates, D. M., Bolstad, B., Dettling, M., Dudoit, S., Ellis, B., Gautier, L., Ge, Y., Gentry, J., et al. (2004). Bioconductor: open software development for computational biology and bioinformatics. Genome Biol. *5*, R80.

Huber, W., von Heydebreck, A., Sultmann, H., Poustka, A., and Vingron, M. (2002). Variance stabilization applied to microarray data calibration and to the quantification of differential expression. Bioinformatics *18 Suppl. 1*, S96-104.

Kriaucionis, S., and Heintz, N. (2009). The nuclear DNA base 5-hydroxymethylcytosine is present in Purkinje neurons and the brain. Science *324*, 929-930.

Mamber, C., Kozareva, D. A., Kamphuis, W., and Hol, E. M. (2013). Shades of gray: The delineation of marker expression within the adult rodent subventricular zone. Progress Neurobiol. *111*, 1-16.

Mirzadeh, Z., Merkle, F. T., Soriano-Navarro, M., Garcia-Verdugo, J. M., and Alvarez-Buylla, A. (2008). Neural stem cells confer unique pinwheel architecture to the ventricular surface in neurogenic regions of the adult brain. Cell Stem Cell *3*, 265-278.

Noushmehr, H., Weisenberger, D. J., Diefes, K., Phillips, H. S., Pujara, K., Berman, B. P., Pan, F., Pelloski, C. E., Sulman, E. P., Bhat, K. P., et al. (2010). Identification of a CpG island methylator phenotype that defines a distinct subgroup of glioma. Cancer Cell *17*, 510-522.

Ramalho-Santos, M., Yoon, S., Matsuzaki, Y., Mulligan, R. C., and Melton, D. A. (2002). "Stemness": transcriptional profiling of embryonic and adult stem cells. Science *298*, 597-600.

Sajad, M., Chawla, R., Zargan, J., Umar, S., Sadaqat, M., and Khan, H. A. (2011). Cytokinetics of adult rat SVZ after EAE. Brain Res. *1371*, 140-149.

Sanai, N., Nguyen, T., Ihrie, R. A., Mirzadeh, Z., Tsai, H. H., Wong, M., Gupta, N., Berger, M. S., Huang, E., Garcia-Verdugo, J. M., et al. (2011). Corridors of migrating neurons in the human brain and their decline during infancy. Nature *478*, 382-386.

Sansom, O. J., Meniel, V. S., Muncan, V., Phesse, T. J., Wilkins, J. A., Reed, K. R., Vass, J. K., Athineos, D., Clevers, H., and Clarke, A. R. (2007). *Myc* deletion rescues Apc deficiency in the small intestine. Nature *446*, 676-679.

Silva-Vargas, V., Crouch, E. E., and Doetsch, F. (2013). Adult neural stem cells and their niche: a dynamic duo during homeostasis, regeneration, and aging. Curr. Opinion Neurobiol. *23*, 935-942.

Smyth, G. K., Michaud, J., and Scott, H. S. (2005). Use of within-array replicate spots for assessing differential expression in microarray experiments. Bioinformatics *21*, 2067-2075.

Soga, T., Igarashi, K., Ito, C., Mizobuchi, K., Zimmermann, H. P., and Tomita, M. (2009). Metabolomic profiling of anionic metabolites by capillary electrophoresis mass spectrometry. Analytical Chem. *81*, 6165-6174.

Subramanian, A., Tamayo, P., Mootha, V. K., Mukherjee, S., Ebert, B. L., Gillette, M. A., Paulovich, A., Pomeroy, S. L., Golub, T. R., Lander, E. S., and Mesirov, J. P. (2005). Gene set enrichment analysis: a knowledge-based approach for interpreting genome-wide expression profiles. Proc. Natl. Acad. Sci. USA *102*, 15545-15550.

Turcan, S., Rohle, D., Goenka, A., Walsh, L. A., Fang, F., Yilmaz, E., Campos, C., Fabius, A. W., Lu, C., Ward, P. S., et al. (2012). *IDH1* mutation is sufficient to establish the glioma hypermethylator phenotype. Nature *483*, 479-483.

Yushkevich, P. A., Piven, J., Hazlett, H. C., Smith, R. G., Ho, S., Gee, J. C., and Gerig, G. (2006). User-guided 3D active contour segmentation of anatomical structures: significantly improved efficiency and reliability. NeuroImage *31*, 1116-1128.

Zeller, K. I., Jegga, A. G., Aronow, B. J., O'Donnell, K. A., and Dang, C. V. (2003). An integrated database of genes responsive to the Myc oncogenic transcription factor: identification of direct genomic targets. Genome Biol. *4*, R69.
