# Supplementary material for: Expression of Idh1R132H in the Murine Subventricular Zone Stem Cell Niche Recapitulates Features of Early Gliomagenesis
Source: Cancer Cell. 2016 Oct 10;30(4):578–94. doi: 10.1016/j.ccell.2016.08.017 (PMC5064912; doi:10.1016/j.ccell.2016.08.017)
Supplement: Document S2. Article plus Supplemental Information [file mmc3.pdf]

Cancer Cell

Article

# Expression of *Idh1*$^{R132H}$ in the Murine Subventricular Zone Stem Cell Niche Recapitulates Features of Early Gliomagenesis

## Graphical Abstract

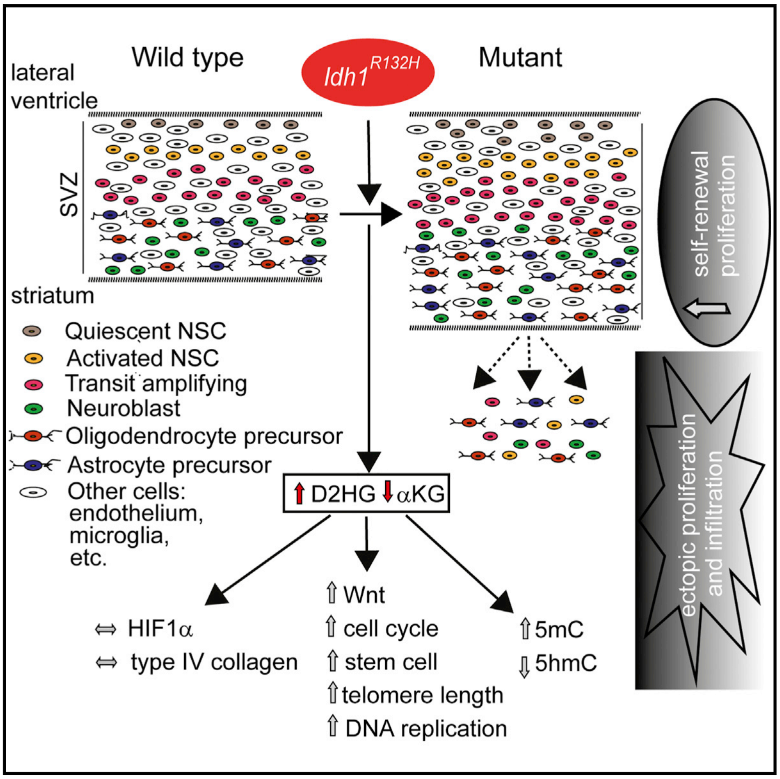



## Authors

Chiara Bardella, Osama Al-Dalahmah, Daniel Krell, ..., Peter J. Ratcliffe, Francis G. Szele, Ian Tomlinson

## Correspondence

francis.szele@dpag.ox.ac.uk (F.G.S.), iant@well.ox.ac.uk (I.T.)



## In Brief

Bardella et al. report that expression of *IDH1*$^{R132H}$ in the subventricular zone of the adult mouse brain causes features of gliomagenesis, including increased numbers of neural stem cells, cellular infiltration into surrounding brain regions, and a gene expression profile overlapping that of human gliomas.


## Highlights

- *Idh1*$^{R132H}$ knockin in the mouse brain SVZ recapitulates features of gliomagenesis
- Self-renewal and proliferation of neural stem cells and progenitors increase
- SVZ cells proliferate ectopically, infiltrate the brain parenchyma, and form nodules
- Increases occur in 2HG levels, Wnt and telomere pathway activity, and DNA methylation


## Accession Numbers

GSE85080

Bardella et al., 2016, Cancer Cell *30*, 578–594
October 10, 2016 © 2016 The Author(s). Published by Elsevier Inc.
http://dx.doi.org/10.1016/j.ccell.2016.08.017

Cancer Cell
Article

# Expression of *Idh1*$^{R132H}$ in the Murine Subventricular Zone Stem Cell Niche Recapitulates Features of Early Gliomagenesis

Chiara Bardella,[1] Osama Al-Dalahmah,[2] Daniel Krell,[1] Pijus Brazauskas,[3] Khalid Al-Qahtani,[4] Marketa Tomkova,[3] Julie Adam,[5,6] Sébastien Serres,[7,8] Helen Lockstone,[9] Luke Freeman-Mills,[1] Inga Pfeffer,[4] Nicola Sibson,[7] Robert Goldin,[10] Benjamin Schuster-Böeckler,[3] Patrick J. Pollard,[5,11] Tomoyoshi Soga,[12] James S. McCullagh,[4] Christopher J. Schofield,[4] Paul Mulholland,[13] Olaf Ansorge,[14] Skirmantas Kriaucionis,[3] Peter J. Ratcliffe,[3,5] Francis G. Szele,[2,*] and Ian Tomlinson[1,15,*]

[1]Molecular & Population Genetics Laboratory, Wellcome Trust Centre for Human Genetics, University of Oxford, Oxford OX3 7BN, UK
[2]Department of Physiology, Anatomy and Genetics, University of Oxford, Oxford OX1 3QX, UK
[3]Nuffield Department of Clinical Medicine, Ludwig Institute for Cancer Research, University of Oxford, Oxford OX3 7DQ, UK
[4]Chemistry Research Laboratory, University of Oxford, Oxford OX1 3TA, UK
[5]Hypoxia Biology Laboratory, Henry Wellcome Building for Molecular Physiology, University of Oxford, Oxford OX3 7BN, UK
[6]Radcliffe Department of Medicine, OCDEM, Churchill Hospital, Oxford OX3 7LJ, UK
[7]Department of Oncology, Cancer Research UK and MRC Oxford Institute for Radiation Oncology, University of Oxford, Oxford OX3 7LE, UK
[8]School of Life Sciences, The Medical School, University of Nottingham, Nottingham NG7 2UH, UK
[9]Bioinformatics, Wellcome Trust Centre for Human Genetics, University of Oxford, Oxford OX3 7BN, UK
[10]Centre for Pathology, St Mary's Hospital, Imperial College, London W2 1NY, UK
[11]Department of Physiology, Institute of Neuroscience and Physiology, Sahlgrenska Academy at University of Gothenburg, 405 30 Gothenburg, Sweden
[12]Institute for Advanced Biosciences, Keio University, 246-2 Mizukami, Kakuganji, Tsuruoka, Yamagata 997-0052, Japan
[13]Department of Oncology, University College London Hospital, London NW1 2BU, UK
[14]Nuffield Department of Clinical Neurosciences, Department of Neuropathology, John Radcliffe Hospital, Headley Way, Oxford OX3 9DU, UK
[15]Lead contact
*Correspondence: francis.szele@dpag.ox.ac.uk (F.G.S.), iant@well.ox.ac.uk (I.T.)
http://dx.doi.org/10.1016/j.ccell.2016.08.017

## SUMMARY

Isocitrate dehydrogenase 1 mutations drive human gliomagenesis, probably through neomorphic enzyme activity that produces D-2-hydroxyglutarate. To model this disease, we conditionally expressed *Idh1*$^{R132H}$ in the subventricular zone (SVZ) of the adult mouse brain. The mice developed hydrocephalus and grossly dilated lateral ventricles, with accumulation of 2-hydroxyglutarate and reduced α-ketoglutarate. Stem and transit amplifying/progenitor cell populations were expanded, and proliferation increased. Cells expressing SVZ markers infiltrated surrounding brain regions. SVZ cells also gave rise to proliferative subventricular nodules. DNA methylation was globally increased, while hydroxymethylation was decreased. Mutant SVZ cells overexpressed Wnt, cell-cycle and stem cell genes, and shared an expression signature with human gliomas. *Idh1*$^{R132H}$ mutation in the major adult neurogenic stem cell niche causes a phenotype resembling gliomagenesis.

## INTRODUCTION

Gliomas are the most frequent primary brain tumor. They have diverse morphology, genetic status, and response to therapy. Grade I gliomas are characterized by slow growth. Grade II/III gliomas are invasive and progress to higher-grade lesions with poor prognosis. Glioblastoma (grade IV, GBM), the most common and most aggressive glioma, may develop rapidly without

### Significance

Few curative treatments are available for human brain tumors. *IDH1*$^{R132H}$ is a driver mutation in gliomas and other malignancies, probably causing tumorigenesis through D-2-hydroxyglutarate accumulation, although the downstream mechanisms remain unclear. We found that adult mice expressing *Idh1*$^{R132H}$ in the brain subventricular zone (SVZ) develop features of gliomagenesis, including increased numbers of neural stem cells and their progeny. Other abnormalities included cellular infiltration into surrounding brain regions, reminiscent of tumor invasion. The gene expression profile of the *Idh1*$^{R132H}$ SVZ closely overlaps those of human gliomas. Likely, non-exclusive tumorigenic mechanisms included promotion of a neural stem cell phenotype, Wnt pathway activation, maintenance of telomeres, and DNA hypermethylation. Our *Idh1*$^{R132H}$ mouse provides a system for assessing brain tumor therapies in vivo.

 © 2016 The Author(s). Published by Elsevier Inc.
This is an open access article under the CC BY license (http://creativecommons.org/licenses/by/4.0/).

evidence of a less malignant precursor lesion (primary), or less often by progression of a lower grade tumor (secondary). Most grade II/III gliomas and secondary GBMs carry mutations in one of isocitrate dehydrogenase genes *IDH1* or *IDH2* (Losman and Kaelin, 2013). *IDH1* or *IDH2* mutations are early events in gliomagenesis, are negatively associated with *PTEN* mutation and *EGFR* amplification, and in the astrocytoma sub-type of glioma, are positively associated with *TP53* mutation (Gupta et al., 2011; Reitman and Yan, 2010). *IDH* driver mutations are also found in acute myeloid leukemia (AML) (Mardis et al., 2009), cholangiocarcinoma (Borger et al., 2012), enchondroma (Amary et al., 2011; Pansuriya et al., 2011), chondrosarcoma (Amary et al., 2011), and occasionally, other tumors. How *IDH* mutations contribute to tumorigenesis is largely unknown.

IDH1 and IDH2 convert isocitrate to α-ketoglutarate (αKG) by oxidative decarboxylation, also generating NADPH. These enzymes share sequence and functional homology. IDH1 is found in the cytoplasm and peroxisome where it acts in lipid and glucose metabolism and protects against reactive oxygen species (ROS). IDH2 localizes to the mitochondria, where it regulates the tricarboxylic acid (TCA) cycle (Reitman and Yan, 2010).

In gliomas, *IDH1*$^{R132H}$ is the most common mutation (Parsons et al., 2008). Initial in vitro studies found that mutant IDH1 had decreased affinity for isocitrate (Zhao et al., 2009) and led to reduced αKG and NADPH levels (Yan et al., 2009; Zhao et al., 2009). Thus, *IDH1* mutations were initially thought to cause tumors by loss of function. Subsequent studies showed mutant IDH1 gained an enzymatic function that converts αKG to D-2-hydroxyglutarate (D2HG) (Dang et al., 2009). D2HG was postulated to be an "oncometabolite" based on reports of brain tumors in patients with congenital *L2HGDH* (L-2-hydroxyglutarate dehydrogenase) deficiency, in whom L2HG accumulates because it cannot be metabolized to αKG (Aghili et al., 2009; Moroni et al., 2004; Patay et al., 2012, 2015). Further in vitro data showed that provision of D2HG in *IDH1/2*-wild-type hematopoietic cells had similar leukemogenic activity to *IDH1* mutations (Losman and Kaelin, 2013). The tumorigenic effects of D2HG may derive from modulating αKG-dependent enzymes such as JmjC domain histone demethylases (JHDMs), TET 5-methylcytosine hydroxylases that convert 5′-methylcytosine (5mC) to 5′-hydroxymethylcytosine (5hmC) (Xu et al., 2011), and prolyl hydroxylases (PHDs) that have targets such as HIF1α and collagen (Borger et al., 2012; Chowdhury et al., 2011; Duncan et al., 2012; Figueroa et al., 2010; Hirata et al., 2015; Lu et al., 2012; Sasaki et al., 2012b; Tarhonskaya et al., 2014; Xu et al., 2011). Evidence for these possibilities varies: for example, HIF pathway changes reported in *IDH1* mutants vary from activation through no change to inactivation.

Several mice carrying pathogenic *Idh1* or *Idh2* mutations have been analyzed. Sasaki et al. (2012b) conditionally expressed *Idh1*$^{R132H}$ in hematopoietic lineages, leading to raised progenitor cell numbers and extra-medullary hematopoiesis. Mutant cells showed increased histone and DNA methylation, consistent with findings in human AML (Figueroa et al., 2010). Knockin of *Idh1*$^{R132H}$ in brain progenitors from E10.5 using nestin-Cre caused neonatal death due to brain hemorrhages and high levels of apoptosis (Sasaki et al., 2012a). No stem cell abnormalities were observed in these mice, but HIF1α was stabilized and collagen maturation was aberrant. Histone lysine methylation was unchanged, but in very early embryos, 5hmC levels were greatly reduced. In a parallel experiment using GFAP-Cre, which acts from E14.5 in neural stem cells (NSCs), 60% of mice suffered brain hemorrhages, and few survived to adulthood. Akbay et al. (2014) ubiquitously expressed mutant *Idh2* (R140Q or R172K) in 5-week-old mice, resulting in cardiomyopathy and white matter abnormalities throughout the CNS. None of these *Idh*-mutant mice developed phenotypes clearly related to brain tumorigenesis.

There is evidence that many human gliomas arise from the subventricular zone (SVZ, also known as the V-SVZ), the largest NSC niche in the adult mammalian brain (Ihrie and Alvarez-Buylla, 2011). In the canonical model of normal SVZ lineage progression, quiescent NSCs become activated and generate rapidly dividing transit amplifying progenitor (TAP) cells. These, in turn give rise to lineage committed neuroblasts or glioblasts that differentiate and in mice migrate to the olfactory bulb. How the stem cell niche generates brain tumors is not well understood, but evidence supports a link between SVZ NSCs and gliomas. First, Wnt, EGF, SHH, and PDGF signaling are necessary for SVZ stem cell self-renewal and proliferation, and are also implicated in tumorigenesis (Jackson and Alvarez-Buylla, 2008). Second, nestin and other SVZ stem cell markers are highly expressed in GBMs (Haskins et al., 2013; Singh et al., 2004). Finally, mutations or aberrant expression of genes such as *Kras, Pdgfb, Trp53, Ptch1*, and *Pten* in the SVZ of mice can elicit tumors (Fomchenko and Holland, 2006; Holland, 2001; Huse and Holland, 2009). Multiple genetic perturbations are often necessary for progression from hyperproliferation to full-blown SVZ tumors.

We wondered whether the existing *Idh1*$^{R132H}$ mice had not developed features of brain tumors because the mutant protein was deleterious in embryos or young animals. Consistent with this notion, we noted that germline *IDH* mutations had not been reported in patients with gliomas or AML, and that the few patients with constitutional *IDH* mutations were mosaics (Amary et al., 2011). We therefore investigated the consequence of expressing *Idh1* R132H specifically in adult NSCs and progenitor cells in mice.

## RESULTS

### Knockin of *Idh1*$^{fl(R132H)/+}$ in the Adult Mouse SVZ Stem Cell Niche

To generate *Idh1*$^{fl(R132H)/+}$ knockin mice, we designed a replacement targeting construct to conditionally express the *Idh1*$^{R132H}$ mutation under the control of the endogenous promoter (Figure 1A) following recombination at *loxP* sites. Initially we targeted the mutation specifically to brain stem/progenitor cells by crossing *Idh1*$^{fl(R132H)/+}$ animals with nestin-Cre mice, thus inducing efficient recombination throughout the CNS from E10.5 (Tronche et al., 1999). As expected, these Idh1-KI mice died perinatally and exhibited brain hemorrhages (Sasaki et al., 2012a) (Figure S1A). We then crossed *Idh1*$^{fl(R132H)/+}$ animals with mice carrying a tamoxifen-inducible nestin-Cre$^{ER(T2)}$, which in adult mice targets Cre to the SVZ and the other major neurogenic niche, the subgranular zone (SGZ) of the hippocampal dentate gyrus (Lagace et al., 2007). We confirmed this using Rosa26-YFP reporter mice (Figure 1B). Tamoxifen was given to

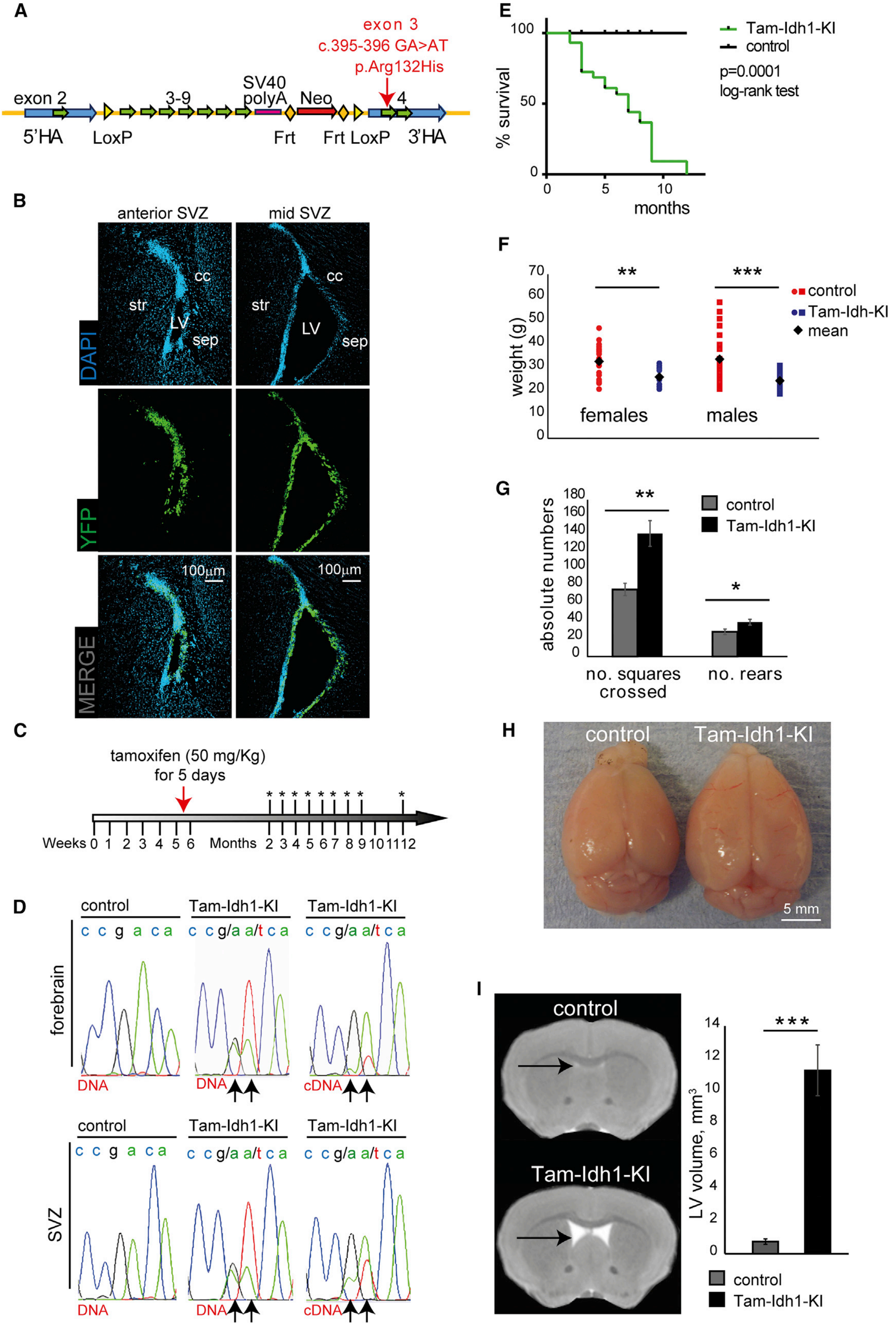


(legend on next page)

the *Nes-Cre*$^{ER(T2)}$; *Idh1*$^{fl(R132H)/+}$ mice at 5–6 weeks of age for 5 consecutive days (Tam-Idh1-KI mice) (Figure 1C). We showed that R132H knockin had occurred by sequencing DNA and mRNA from forebrain and microdissected SVZ (Figure 1D).

Throughout the studies described below, we compared Tam-Idh1-KI mice with *Idh1*-wild-type controls (see Experimental Procedures), all of which were given the same regimen of tamoxifen. The Tam-Idh1-KI animals began to suffer morbidity and mortality 4–6 weeks after tamoxifen administration, necessitating culling of about one-third of them at this stage and all by 12 months of age (Figure 1E). Weight loss was usually the first apparent abnormality (Figure 1F), often accompanied by hunched posture and hyperactivity (Figure 1G). Control mice showed none of these problems. Postmortem analysis was performed on 45 Tam-Idh1-KI mice aged 2–12 months. The brains of 19 (42%) animals were grossly uniformly enlarged and less firm (Figure 1H); controls did not show these changes. Craniofacial morphology and skull size appeared normal in Tam-Idh1-KIs, and no brain hemorrhages or gross abnormalities outside the brain were found. Closer visual examination, confirmed by magnetic resonance images, showed enlarged lateral ventricles (LVs), and possibly enlarged third ventricles, in all Tam-Idh1-KI animals (Figures 1I, S1B, and S1C). These appearances were suggestive of obstructive (non-communicating) hydrocephalus.

## *Idh1*$^{R132H}$ Expands the SVZ

Histological analysis of H&E-stained sections of brain in the sagittal and coronal planes demonstrated lateral ventricular dilation in all Tam-Idh1-KI animals, including both the youngest mice examined (2 months old) and mice with no abnormal physical signs or gross brain enlargement (details not shown). There was also enlargement of the SVZ, with irregular lateral borders, and an expansion of the rostral migratory stream (RMS) (Figure 2A). Furthermore, Ki67 immunohistochemistry (IHC) showed increased numbers of proliferative cells in the SVZ of Tam-Idh1-KI mice (Figure 2B), and these cells spread into the adjacent striatum. The frequency of apoptotic cells was very low in all mice, with no significant difference observed between the two groups (data not shown). There was no morphological evidence of other brain damage or degeneration in Tam-Idh1-KI mice (Figures S1D and S1E).

## *Idh1*$^{R132H}$ Increases the Numbers of Stem and Progenitor Cells in the SVZ

We next examined Tam-Idh1-KI mice using immunofluorescence (IF) for cellular phenotypes related to tumorigenesis, including proliferation, differentiation, and migration. Owing to the abnormalities found in H&E sections, we focused on the SVZ. IF-based quantitation confirmed that, compared with controls, Tam-Idh1-KI mice showed a larger SVZ volume (1.15-fold, $p = 0.041$), similar cell density ($p = 0.20$), and a larger number of cells ($p = 0.047$).

To better evaluate the proliferative dynamics of the SVZ and to obtain information regarding which cell populations (Figure S2A) were expanded in Tam-Idh1-KI animals, mutant and control mice were injected with BrdU in three consecutive daily doses, followed 13 days later by a pulse of EdU 2 hr before sacrifice (Figure 3A). The BrdU label-retaining cells in the SVZ correspond to relatively quiescent NSCs, but not their progeny, TAPs, which divide frequently. The EdU label is found in cells cycling at the time of injection. We carried out double IF of EdU and GFAP (Figures 3B and 3C) and triple IF of Ki67, BrdU, and GFAP (Figures 3D and 3E), and quantified SVZ cells with confocal microscopy.

We analyzed the number of cells per unit volume to control for the increased size of the mutant SVZ. The proportions, and hence total numbers, of EdU$^+$ (rapidly proliferating or re-entering cell cycle), GFAP$^+$ (mainly NSCs and niche astrocytes), and EdU$^+$GFAP$^+$ cells (proliferating NSC or niche astrocytes) were increased in Tam-Idh1-KIs (Figure 3C). The proportions and numbers of Ki67$^+$ (cycling) and BrdU$^+$ (quiescent or slowly cycling) cells also increased in the mutant SVZ (Figure 3E). Both Ki67$^+$GFAP$^+$ and BrdU$^+$GFAP$^+$ proportions were higher in the mutant SVZ, suggesting that the number of label-retaining, as well as actively dividing, astrocytic stem cells was increased. This was confirmed by the significantly higher proportion and number of BrdU$^+$Ki67$^+$GFAP$^+$ cells in the mutants, suggesting that more NSCs became activated and re-entered the cell cycle. A more detailed analysis of the Ki67, BrdU, and GFAP triple IF found an overall highly significant difference in the proportions of cell types between Tam-Idh1-KIs and controls ($\chi^2_5 = 43$, $p < 0.001$). The increased cell populations included quiescent and activated NSCs and, to a smaller degree, TAPs (Figure S2B).

We also assessed the oligodendrocyte progenitor marker Pdgfr$\alpha$ and Olig2, a marker of the entire oligodendroglial lineage that is universally expressed in diffuse gliomas (Ligon et al.,

**Figure 1. *Idh1*$^{R132H}$ Expression in the SVZ and Its Effects on Weight, Behavior, and Lateral Ventricle Size in Adult Mice**

(A) The construct used to generate Tam-Idh1-KI mice is shown. After homologous recombination into the endogenous *Idh1* locus, expression of Cre causes deletion of (1) a mini-gene containing *Idh1* wild-type exons 3–9; (2) termination codon and SV40 polyA signal; and (3) Neo$^R$ cassette. *Idh1*$^{R132H}$ is then expressed from the native promoter. The following features are shown: *loxP* and Frt sites; 5′ and 3′ homology arms (HAs); wild-type mini-gene (exons 3–9) and SV40 polyA signal; neomycin resistance cassette (Neo$^R$); location of the R132H mutation.

(B) The panels show expression of the YFP reporter in the anterior SVZ (left) and mid-SVZ (right) of *Nes-Cre*$^{ER(T2)}$*;R26R-EYFP* mice 29 weeks after tamoxifen induction. cc, corpus callosum; str, striatum; sep, septum; LV, lateral ventricle.

(C) Tamoxifen dosage schedule is shown. Asterisks indicate time points of brain collections.

(D) Sequencing chromatogram shows genomic DNA or cDNA of a region around *Idh1* codon 132 from forebrain or SVZ of control or Tam-Idh1-KI mice as indicated. Arrows indicate nucleotides altered in *Idh1*$^{R132H}$ (c. 395–396 CGA > CAT).

(E) Kaplan-Meier plots show the survival of Tam-Idh1-KI (n = 28) and control (n = 32) mice from 18 litters.

(F) Body weights of paired Tam-Idh1-KI and control mice are shown.

(G) Open field test measures of locomotion (no. of squares crossed) and activity (no. of rears) are shown.

(H) Whole-brain dissections show frontal and parietal lobe morphology in Tam-Idh1-KI and control animals.

(I) MRI illustrates LV volumes (arrows) in Tam-Idh1-KI mice and controls. Sections were taken at comparable locations. Estimated LV volumes of three Tam-Idh1-KI mice and three controls are shown in the chart.

All data are presented as mean ± SD (*p < 0.05, **p < 0.01, ***p < 0.005). See also Figure S1.

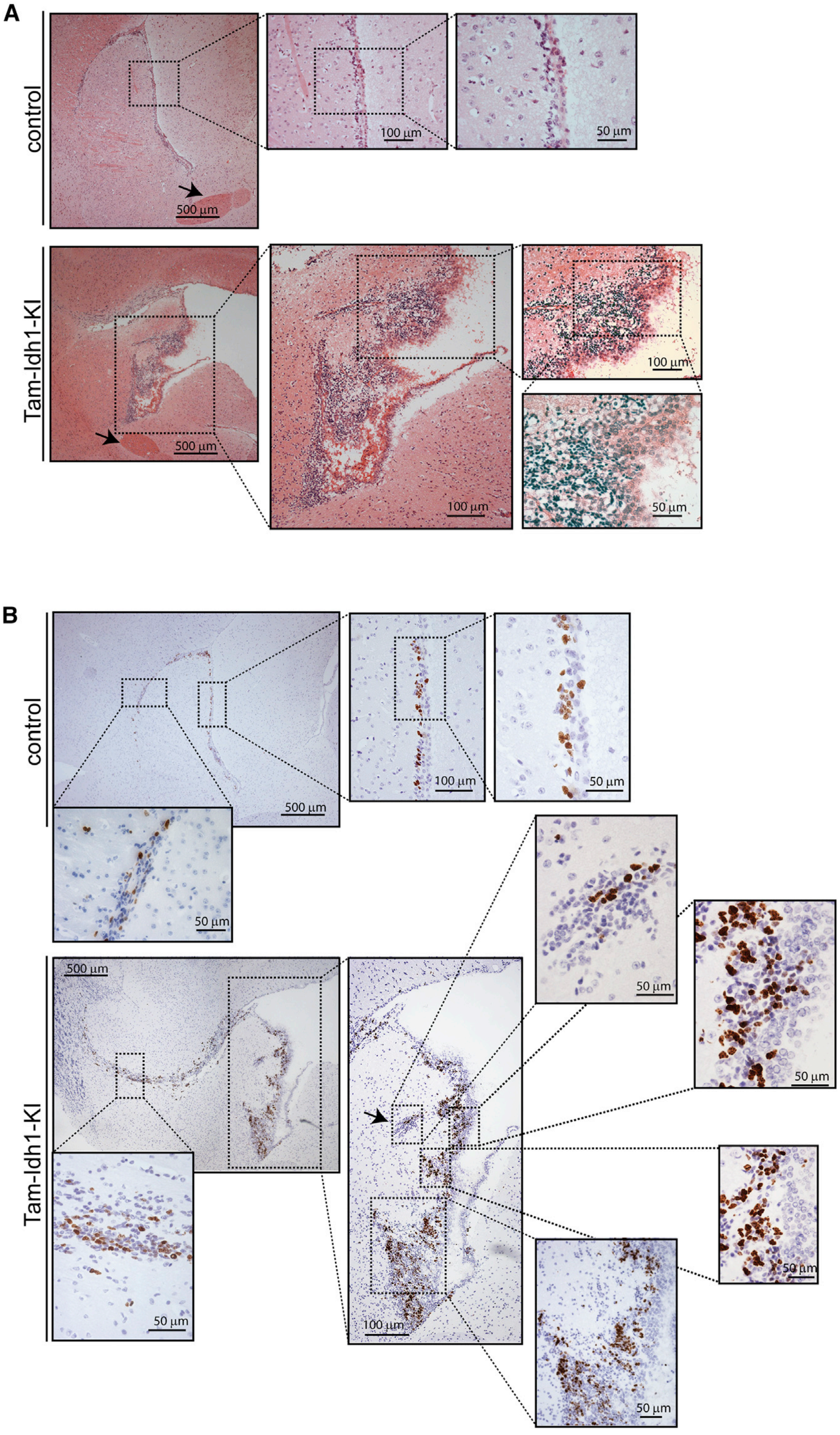


**Figure 2. Histology and Proliferation of SVZ in Tam-Idh1-KI and Control Mice**

(A) H&E staining of sagittal sections from control and Tam-Idh1-KI mice is shown. The anterior commissure is indicated by arrows.

(B) Ki67 immunohistochemistry is shown in sagittal sections at comparable medio-lateral positions from Tam-Idh1-KI and control mice. Ki67$^+$ cell clusters ectopic to the SVZ are arrowed. Areas in the insets are magnified.

2004; Suzuki et al., 2014) (Figures 3F and 3G). The proportions and numbers of Pdgfr$\alpha^+$Olig2$^+$ and total Olig2$^+$ cells were both increased in Tam-Idh1-KI mice compared with controls. A similar increase was found for the neuroblast marker Dcx (details not shown).

We assessed Ki67, GFAP and Olig2 expression in younger mice that had received tamoxifen at 5–6 weeks of age and were analyzed 3–8 weeks later. Compared with controls, the younger Tam-Idh1-KI mice showed similar GFAP and Olig2 changes to those observed previously in older animals, although the Ki67 increase was smaller (details not shown). The younger mice also had an expanded SVZ, a diffuse RMS and ectopic cells. These data suggest that the Tam-Idh1-KI phenotype can develop quite rapidly but may also progress over time.

In summary, *Idh1*$^{R132H}$ increased the proportions and numbers of quiescent NSCs and their proliferative progeny (activated stem cells, progenitor cells, and partly differentiated cells) in both young and older mice, thereby enlarging the SVZ (Figure S2C). A parsimonious explanation for these findings is a primary effect of the mutation on quiescent NSCs, although additional effects in their progeny cannot be excluded.

## *Idh1*$^{R132H}$ Induces Infiltration into the Parenchyma

The confines of the SVZ and neuroblast migration in the healthy brain are tightly controlled, with the high density of cells making it easy to distinguish these regions from adjacent tissue. Migratory SVZ cells mostly move to the olfactory bulb (OB), with few emigrating from the SVZ or RMS to the adjacent parenchyma (Lois and Alvarez-Buylla, 1994). The Tam-Idh1-KI SVZ was enlarged, and its border was not clearly delineated (Figures 2 and 3). As expected, in controls, the great majority of BdU$^+$, Ki67$^+$, or EdU$^+$ cells were restricted to the SVZ (Figures 4A, S3A, and S3B). In contrast, Tam-Idh1-KI mice contained large numbers of actively mitotic Ki67$^+$ or EdU$^+$ cells, as well as label-retaining BrdU$^+$ cells, adjacent to and contiguous with the SVZ, in locations equivalent to those of the striatum and corpus callosum in wild-type mice (Figures 4A and S3). Similarly, Tam-Idh1-KI brains displayed increased numbers of GFAP$^+$ cells in both the SVZ and surrounding parenchyma, whereas GFAP

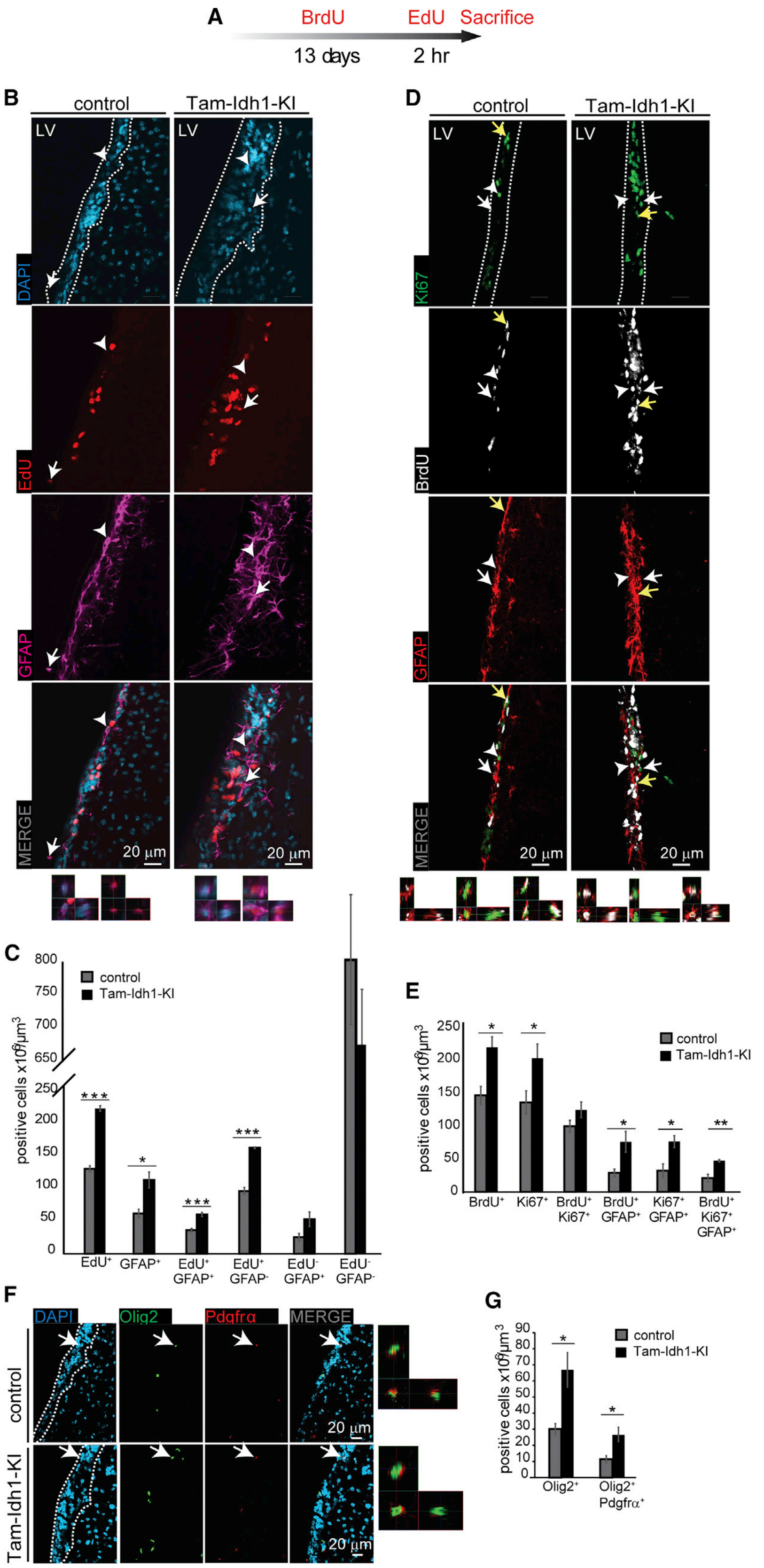


**Figure 3. The Effect of *Idh1*$^{R132H}$ on Label-Retaining Cells, Proliferating Cells, and Oligodendrocytes in the SVZ**

(A) The time course of BrdU and EdU injections is shown.

(B) For the analysis of rapidly cycling cells, EdU and GFAP expression in the SVZ was determined. Representative images showing EdU and GFAP expression were derived from coronal sections from four Tam-Idh1-KI and four control mice. The left-hand image at the bottom of each panel is a magnified orthogonal 3D view of the GFAP$^+$ EdU$^-$ cell marked by an arrowhead; and the adjacent right-hand image is the equivalent view of the GFAP$^+$EdU$^+$ cell marked by an arrow. Dashed lines outline the SVZ.

(C) Z stack quantifications of each cellular population from (B) are shown in the chart as "density", which is a measure of the proportion of each cell type. Total EdU$^+$ and GFAP$^+$ cells are shown, followed by a breakdown of these into the component categories and an EdU$^-$GFAP$^-$ group.

(D) For analysis of label-retaining cells, BrdU, Ki67, and GFAP expression was assessed. The left-hand image at the bottom of each panel is a magnified orthogonal 3D view of the GFAP$^+$BrdU$^+$ cell marked by a white arrow; the adjacent middle image is the equivalent view of the GFAP$^+$Ki67$^+$ cell marked by a white arrowhead; and the adjacent right-hand image is the equivalent view of the GFAP$^+$BrdU$^+$Ki67$^+$ cell marked by a yellow arrow. Other annotation is as per (B).

(E) The chart shows quantification of each directly counted, marker-positive cell population from (D). Note that BrdU$^+$ cells may have any Ki67 and GFAP status and BrdU$^+$Ki67$^+$ cells include both GFAP$^+$ and GFAP$^-$ cells.

(F) Olig2$^+$ (pan-oligodendrocyte) and Pdgfrα$^+$ (progenitors) expression are shown. The image at the extreme right of each panel is a magnified orthogonal 3D view of the Olig2$^+$Pdgfrα$^+$ cell indicated by a white arrow. Other annotation is as per (B).

(G) The chart shows quantification of cell populations from (F). Note that Olig2$^+$ includes cells with any Pdgfrα status.

All data are presented as mean ± SD (*$p < 0.05$, **$p < 0.01$, ***$p < 0.005$). See also Figure S2.

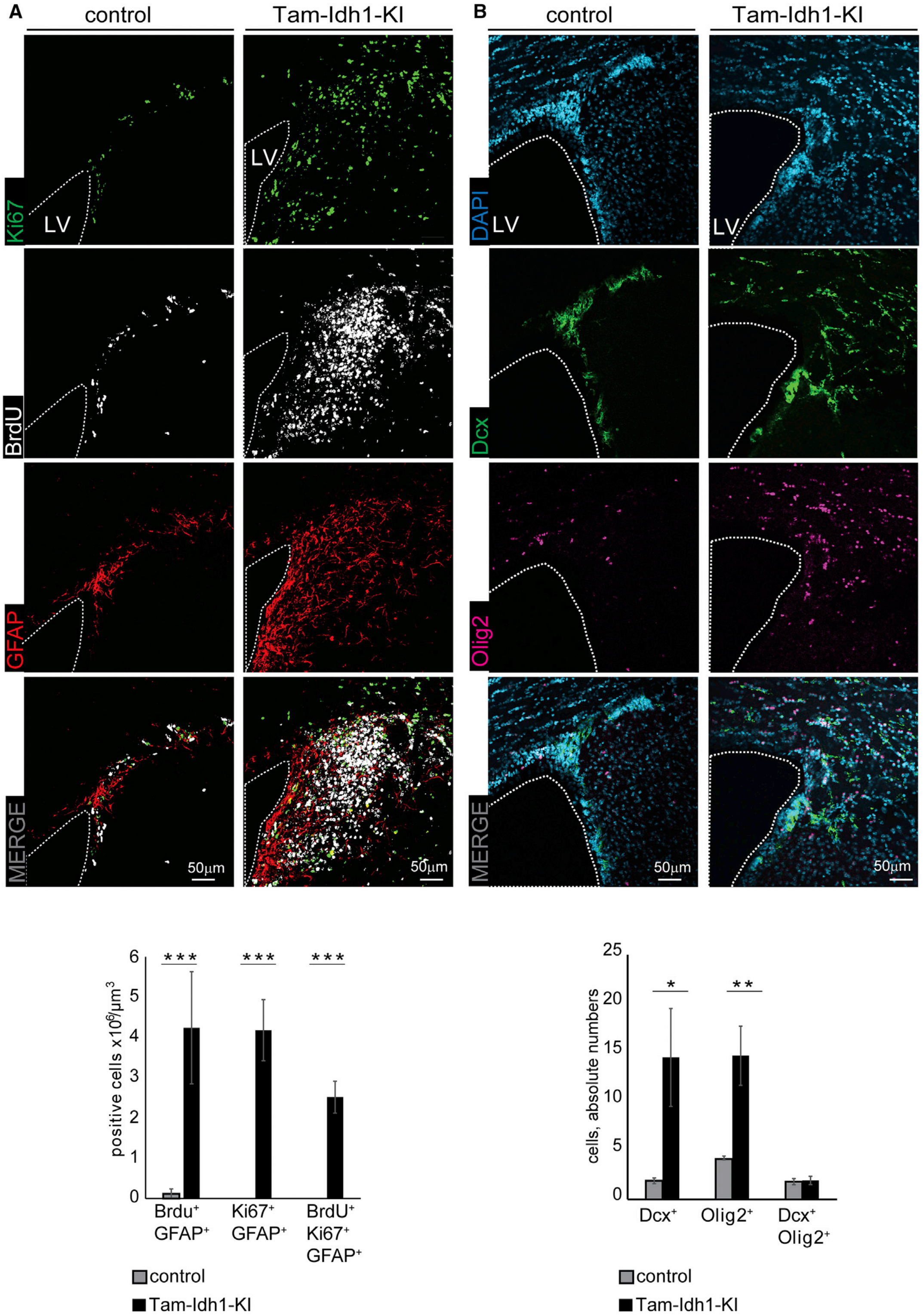


**Figure 4. The Effect of *Idh1*$^{R132H}$ on Tissues Surrounding the Dorsolateral Corner of the SVZ**

(A) Ki67, BrdU, and GFAP expression are shown in coronal sections of Tam-Idh1-KI and control brains. Dashed lines outline the LV, around which the SVZ usually forms a ribbon of cells. The data are quantitated in the bar chart below.

(B) Cells expressing oligodendocyte (Olig2$^+$) and neuroblast (Dcx$^+$) markers are shown. The data are quantitated in the bar chart below.

All data are presented as mean ± SD (*p < 0.05, **p < 0.01, ***p < 0.005). See also Figure S3.

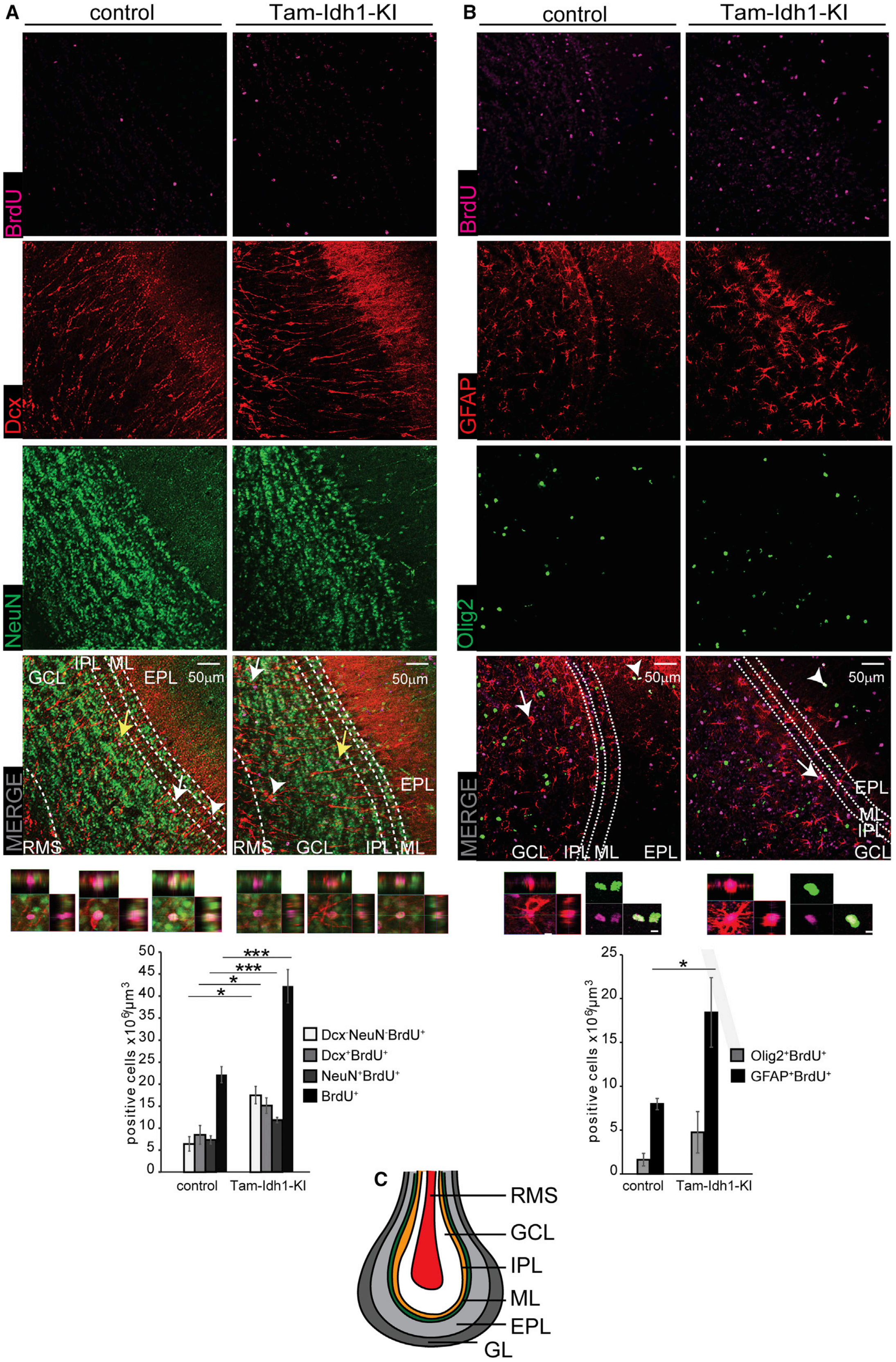


(legend on next page)

expression was almost exclusive to the SVZ and corpus callosum in controls (Figures 4A, S3A, and S3B). BrdU$^+$ label-retaining GFAP$^+$ astrocytes that were actively proliferating (Ki67$^+$) were present in the striatum of Tam-Idh1-KI brains but absent in control mice (Figures 4A, S3A, and S3B). These data were consistent with Tam-Idh1-KI causing the SVZ to expand and/or SVZ cells to migrate out of their local boundaries and proliferate in ectopic locations adjacent to the neurogenic niche.

To further understand these regions of hyperproliferation, we assessed expression of Dcx, a marker of immature migratory SVZ neuroblasts, and Olig2. Compared with controls, Tam-Idh1-KI mice contained significantly more Dcx$^+$ cells and Olig2$^+$ cells in the striatum and corpus callosum (Figures 4B and S3C–S3E). Higher magnification showed that many of the ectopic Dcx$^+$ cells had bipolar morphology with leading processes, suggestive of migration. Transformed cells and SVZ cells in pathological conditions can express multiple lineage markers (Jablonska et al., 2010), but the proportion of cells that were Dcx$^+$/Olig2$^+$ was small and did not change in the Tam-Idh1-KI mice (Figure 4B).

Migration in the RMS is highly regulated, similar to the SVZ. All seven Tam-Idh1-KI mice analyzed (five old and two young) had a more diffuse RMS (Figure 5A), with dispersed Dcx$^+$ cells migrating in various orientations. In addition, in contrast to controls, all Tam-Idh1-KI mice examined exhibited Dcx$^+$ cells, sometimes in massive numbers, in the ventral cerebral cortex, dorsal to the RMS, suggesting emigration from it (Figure 5A). This was consistent with their ectopic location near the dorsolateral SVZ (Figure 4). Additional abnormalities consistent with increased stem-like cells and aberrant migration were also found in the OBs of Tam-Idh1-KI animals (Figures 5, S4A, and S4B). Compared with controls, Tam-Idh1-KI animals contained significantly more BrdU$^+$, Dcx$^+$BrdU$^+$, and NeuN$^+$BrdU$^+$ cells in the granule layer (data not shown) and the OB as a whole (Figure 5A), indicating that neurogenesis was increased in the Tam-Idh1-KI mice, probably due to the increased numbers of stem and/or proliferating SVZ cells. The proportion of BrdU$^+$ cells that expressed differentiation/lineage markers (NeuN, Dcx, GFAP, and Olig2) was similar in controls and mutants, suggesting that the cells retained much of their normal differentiation capacity (data not shown).

Interestingly we also found that the OBs of Tam-Idh1-KI mice contained a larger number of BrdU$^+$ cells that were negative for neuronal markers (Figure 5A), suggesting that glial cells may have been generated in the OB. To study this possibility, we quantified GFAP$^+$ astrocytes and Olig2$^+$ oligodendrocytes that retained BrdU. GFAP$^+$BrdU$^+$ cell numbers were significantly increased in the OBs of Tam-Idh1-KI mice, and the number of Olig2$^+$ cells was also increased, albeit not significantly (Figure 5B).

To confirm that the SVZ was the source of the mislocalized cells, we traced the *Nes-Cre*-expressing cell lineage using control and Tam-Idh1-KI mice that also carried the Rosa26-YFP reporter allele (Srinivas et al., 2001). As expected in the control crosses, YFP$^+$ cells were restricted to regions of endogenous nestin expression: the SVZ and RMS (Figures S4C–S4E). In the crossed Tam-Idh1-KI mice, many YFP$^+$ cells were found in an expanded SVZ and RMS, and also in ectopic locations continuous with the neurogenic niche (Figures S4C–S4E). These included the corpus callosum, striatum next to the SVZ as well as regions adjacent to the RMS. This lineage experiment supported the model that the SVZ and RMS expansion and infiltration into adjacent brain regions was initiated from nestin-positive cells, which were originally most likely present in the SVZ.

Disruption of the glial tubes composed of B1 astrocytes and B2 niche astrocytes is often characteristically associated with abnormal migration of neuroblasts (Comte et al., 2011). To address this, we examined the RMS with GFAP IF and compared it with the distribution of YFP$^+$ cells. Although the YFP$^+$ cells were expanded in the RMS of mutant animals, we did not find that the glial tubes were altered in morphology in mutant mice compared with controls (data not shown). This suggests that the emigration/infiltration of the neuroblast progenitors, as well as the glial progenitors, into the regions adjacent to RMS and SVZ is probably cell autonomous.

These results suggest that expression of *Idh1*$^{R132H}$ in adult mouse stem/progenitor cells robustly induced expansion of the SVZ and RMS and infiltration into adjacent regions, similar to observations of glioma cell invasion. The SGZ showed similar features (Figure S4F). While cell numbers were increased in the *Idh1*-mutant animals, the cells appeared to retain their capacity for differentiation.

## Tam-Idh1-KI Mice Develop Proliferative Subventricular Nodules

In all Tam-Idh1-KI mice studied, apart from three killed only 2, 3, and 6 weeks after tamoxifen administration, we found small (up to 1 mm diameter) nodules originating from the SVZ and protruding into the LVs (Figure 6A). The lesions, which were absent from control animals, could be found at any location in the walls of the LVs. The nodules expressed proliferation markers, such as Ki67 (Figures 6B and 6C), and retained BrdU label (Figure 6C), suggesting that they exhibited a variety of proliferative behaviors. As shown in Figure 6C, several cells present in the nodules were Ki67$^+$BrdU$^+$, indicative of label-retaining cells

**Figure 5. The Effect of *Idh1*$^{R132H}$ on Neurogenesis and Astrocyte Genesis in the Olfactory Bulb**

(A) BrdU, Dcx, and NeuN expressing cells (neurogenesis) are shown in representative images from Tam-Idh1-KI (n = 4) and control (n = 4) OBs. The left-hand image at the bottom of each panel is a magnified orthogonal 3D view of the BrdU$^+$Dcx$^-$NeuN$^-$ cell marked by a white arrow; the adjacent middle image is the equivalent view of the BrdU$^+$Dcx$^+$NeuN$^-$ cell marked by a white arrowhead; and the adjacent right-hand image is the equivalent view of the BrdU$^+$Dcx$^-$NeuN$^+$ cell marked by a yellow arrow. RMS, rostral migratory stream; GCL, granule cell layer; IPL, internal plexiform layer; ML, mitral layer; EPL, external plexiform layer. The cell counts are quantitated in the bar chart.

(B) BrdU, GFAP, and Olig2 (astrocyte genesis) data are shown from the same mice as in (A). The left-hand image at the bottom of each panel is a magnified orthogonal 3D view of the BrdU$^+$GFAP$^+$ cell marked by a white arrow; and the adjacent right-hand image is the equivalent view of the BrdU$^+$Dcx$^-$NeuN$^+$ cell marked by a white arrowhead. Other annotations are as per (A).

(C) A schematic of the multi-layered cellular architecture of a mouse OB is shown. GL, glomerular layer. Other annotations are as per (A).

All data are presented as mean ± SD (*p < 0.05, ***p < 0.005). See also Figure S4.

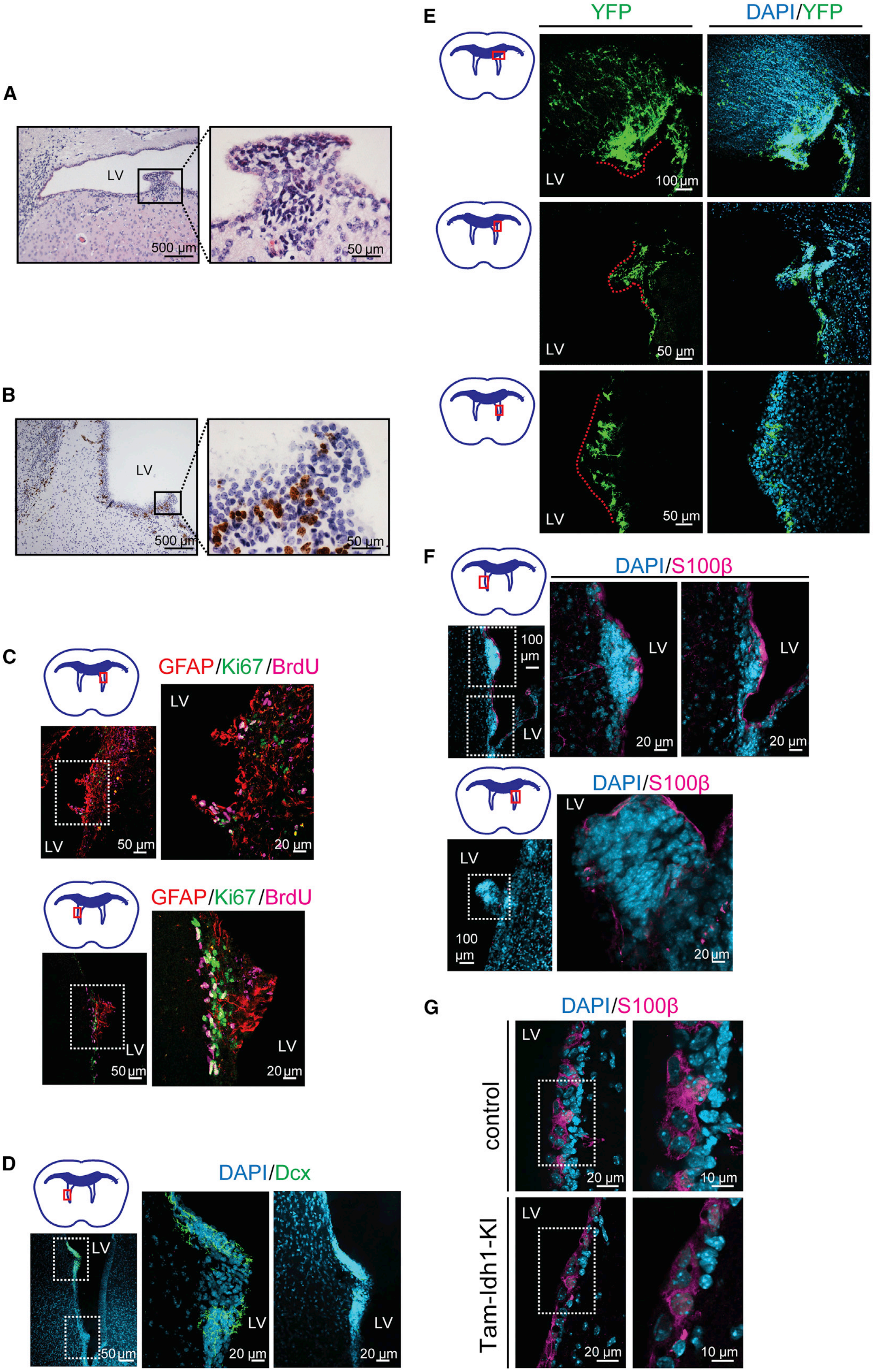


(legend on next page)

that had re-entered the cell cycle. Many nodule cells also expressed the astrocytic and NSC marker GFAP and in some lesions, a few cells expressed the neuroblast marker Dcx (Figures 6C and 6D). Using a reporter mouse cross, we found that the nodules expressed YFP, consistent with origins from nestin-expressing stem or TAP SVZ cells (Figure 6E). Microscopic inspection of the nodules revealed clustered cells, with hyperchromatic nuclei and scant cytoplasm that were clearly distinct from the overlying ependymal lining (Figure 6A). Some of the nodules were not continuously covered by S100β$^{+}$ ependymal cells, suggesting that subventricular lesions had broken through the ventricular lining (Figure 6F). The ependymal layer, as assessed by S100β expression, otherwise appeared normal (Figure 6G).

## The *Idh1*$^{fl(R132H)/+}$ Allele Is Leaky and Causes a Brain Phenotype in a Minority of Juvenile or Aged Mice

We had noted that 10% (9/94) of *Idh1*$^{fl(R132H)/+}$ mice without the Nes-Cre transgene and 8% (5/62) of non-induced *Nes-Cre*$^{ER(T2)}$*;* *Idh1*$^{fl(R132H)/+}$ animals developed rounded and enlarged skulls at 3–6 weeks of age. This phenotype, reminiscent of human hydrocephalus prior to fusion of the skull sutures, necessitated immediate culling. Ventricular nodules, similar to those in Tam-Idh1-KI mice, were found in these animals (Figure 7A.) We aged some surviving *Idh1*$^{fl(R132H)/+}$ animals (after tamoxifen injections) for 1–2 years. Although none showed symptoms or signs of disease while alive, upon postmortem investigation, 8/34 animals (24%) had ventricular enlargement. Of those eight mice, histological examination showed one to have a clearly enlarged, diffuse SVZ (Figure 7B) and another to have a single subventricular nodule (Figure 7C). The brains of these mice accumulated 2HG, but there was no evidence of other brain damage (data not shown). We also aged three *Nes-Cre*$^{ER(T2)}$*;* *Idh1*$^{fl(R132H)/+}$ animals that had not received tamoxifen, and all showed a similar phenotype to the eight *Idh1*$^{fl(R132H)/+}$ mice without Nes-Cre. Further investigation strongly suggested that the phenotype resulted from expression of an *Idh1* mRNA that lacked exons 1 and 2 and was derived from the mouse construct (Figures 1A, 7D, and 7E). We found that the short RNA was a physiological isoform, as it was also produced by the wild-type *Idh1* allele. *Idh1* exons 1 and 2 have no homology to any other protein, are not conserved, and contain no functional domains of predicted importance. A search of the genomic DNA sequence revealed a potential translation initiation site in intron 2 that would leave the enzyme active site intact (Figure 7F). We conclude that in a minority of mice, a leaky *Idh1*$^{R132H}$ allele can produce a forme fruste of the full Tam-Idh1-KI phenotype.

## Identifying the Molecular Mechanism Underlying the Tam-Idh1-KI Phenotype

Attempts to culture primary *Idh1*-mutant neurospheres from the SVZ of Tam-Idh1-KI mice were unsuccessful. We therefore stably expressed IDH1$^{R132H}$ both in human neuronal stem/progenitor cells (ReNcell CX) and in primary SVZ cells dissected from wild-type BL6 mouse pups. In each case, IDH1$^{R132H}$ increased the size and number of neurospheres cultured from human NSC/NPCs (Figures 8A and 8B). These data were consistent with increased proliferation and self-renewal capacity of the mutant NSC/NPCs in vivo.

We also measured the capability of ReNcell CX expressing IDH1$^{R132H}$ to migrate in vitro, in unstimulated or chemoattractant (20 μg/mL FGF or 5% serum) conditions. *IDH1*-mutant cells were significantly more motile than control cells (~1.8-fold in all conditions; ANOVA, $p < 0.0001$; details not shown). These data were in accordance with the infiltrative behavior of *Idh1*-mutant NSC/NPCs in vivo.

Using capillary electrophoresis time-of-flight mass spectrometry (CE-TOFMS), we performed a comprehensive analysis of metabolites in Tam-Idh1-KI and control mice. In Tam-Idh1-KI samples, we detected ~2-fold increase in total 2HG and ~30% decreased αKG compared with controls (Figure 8C). The leaky mice showed similar changes but at lower levels (details not shown). The changes in adults appeared less substantial than those found in the brains of *Nes-Cre; Idh1*$^{fl(R132H)/+}$ embryos using IC-MS (Figure S5A). No significant differences were detected in any other metabolite, including glycolytic, TCA cycle, and pentose phosphate pathway intermediates.

We found no clear evidence that collagen-PHDs, ROS, HIF-PHDs, or JHDMs were altered in the Tam-Idh1-KI mice (Figures S5B–S5F, Table S1). In contrast, in the forebrains of Tam-Idh1-KIs and controls, global abundance analysis by high-performance liquid chromatography (HPLC) showed significantly decreased 5hmC and raised 5mC (Figure 8D). Although the latter was formally non-significant, low-coverage single-base resolution analysis using methyl-seq (WGoxBS) showed significantly increased 5mC in the SVZ of Tam-Idh1-KI animals (Figures 8E and S6A–S6D). The greatest absolute increase in 5mC was outside CpG islands, although the relative increase was similar across all genomic regions (Figure 8E). In summary, *Idh1*$^{R132H}$ mutation led to a global decrease of 5hmC and increase of 5mC, consistent with tumor-promoting effects.

## mRNA Expression Profiling in Tam-Idh1-KI Mice Identifies Pathways of Tumorigenesis

To uncover molecular pathways that might underlie the phenotype of the Tam-Idh1-KI mice, we performed transcriptional

**Figure 6. Nodule Formation in the SVZ**

(A) Typical morphology of a nodule protruding into the lateral ventricle (LV) is shown in an H&E-stained section from a Tam-Idh1-KI animal.

(B) Ki67 expression was assessed in a nodule and at other sites along the LV wall.

(C) Nodule proliferation was assessed by immunofluorescence for Ki67 and BrdU. Two nodules are shown (left), magnified in the inset (right). The astrocyte lineage marker GFAP is also shown.

(D) Immature neuroblast differentiation in nodules was assessed using Dcx.

(E) The origin of nodules from nestin-expressing cells was assessed using the YFP reporter. The dashed lines outline the nodules. YFP$^{+}$ cells infiltrating the corpus callosum are also marked.

(F) Ependymal cell-specific S100β expression indicates the extent of the continuity of the ependymal cell layer overlying the nodules and potential origins of the nodules from ependymal cells. Three nodules are shown (left), magnified in the insets (right).

(G) A high-power view compares the ependymal cell layer in Tam-Idh1-KI and control mice.

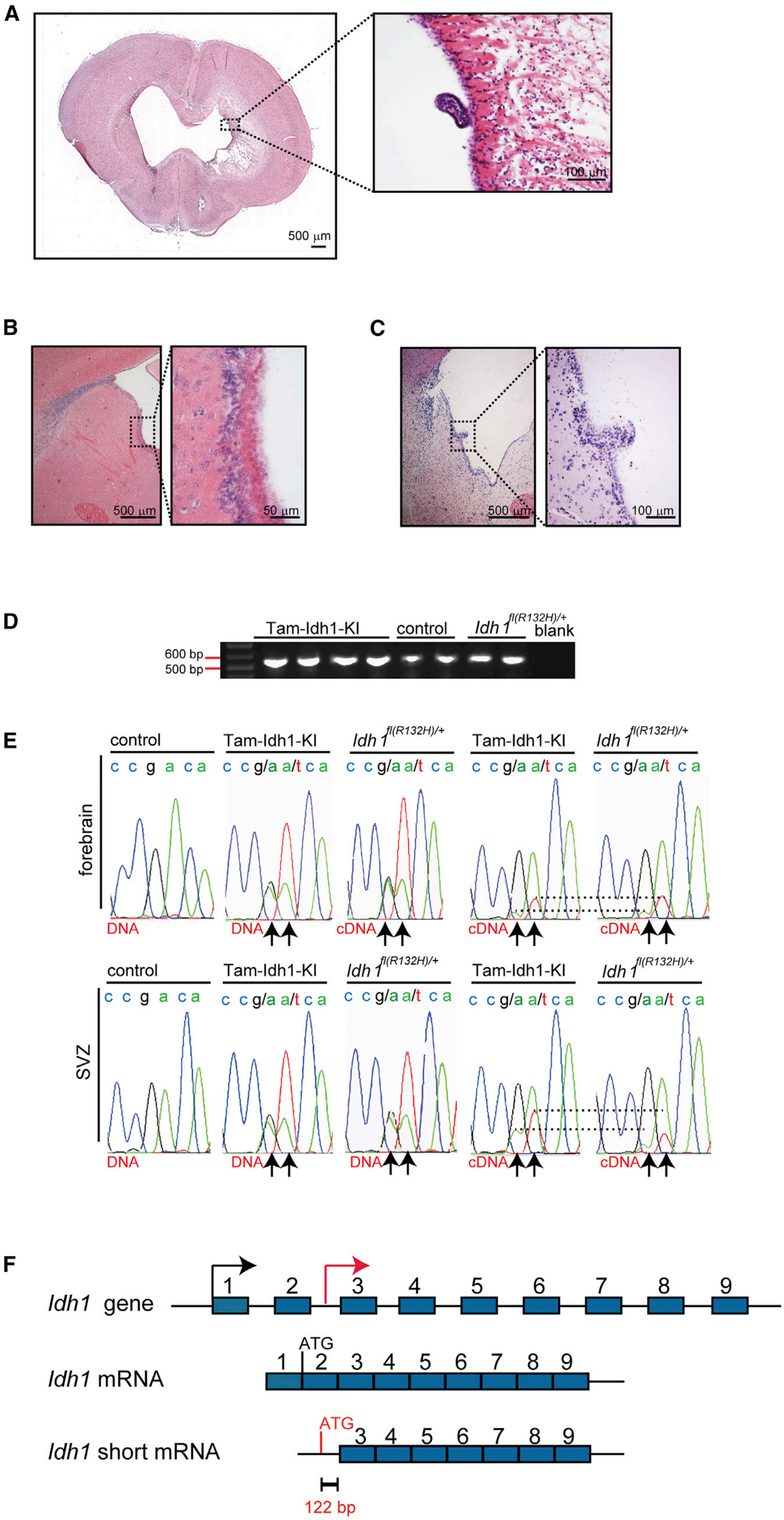


**Figure 7. Leaky Phenotype in a Minority of $Idh1^{fl(R132H)/+}$ Mice without Nes-Cre**

(A) A coronal full-brain section from a symptomatic $Idh1^{fl(R132H)/+}$ mouse 6 weeks old is shown. A nodule is outlined and magnified.

(B) Detailed SVZ morphology of an asymptomatic 4 month old $Idh1^{fl(R132H)/+}$ mouse is shown (sagittal section).

(C) A nodule is present in an asymptomatic 13 month old mouse (sagittal section).

(D) The *Idh1* transcript lacking exons 1 and 2 was amplified from cDNA by endpoint RT-PCR (amplicon size 562 bp).

(E) DNA sequencing chromatogram shows genomic DNA or cDNA specifically derived from the short transcript in the *Idh1* region around codon 132 (samples from mouse forebrain). Arrows indicate nucleotides altered in Tam-Idh1-KI and $Idh1^{fl(R132H)/+}$; dashed lines provide an indication of the relative dosages of wild-type and mutant short transcript in each case.

(F) Schematic of short in-frame *Idh1* transcript lacking exons 1 and 2 shows the putative translational origin in intron 2.

CellPress

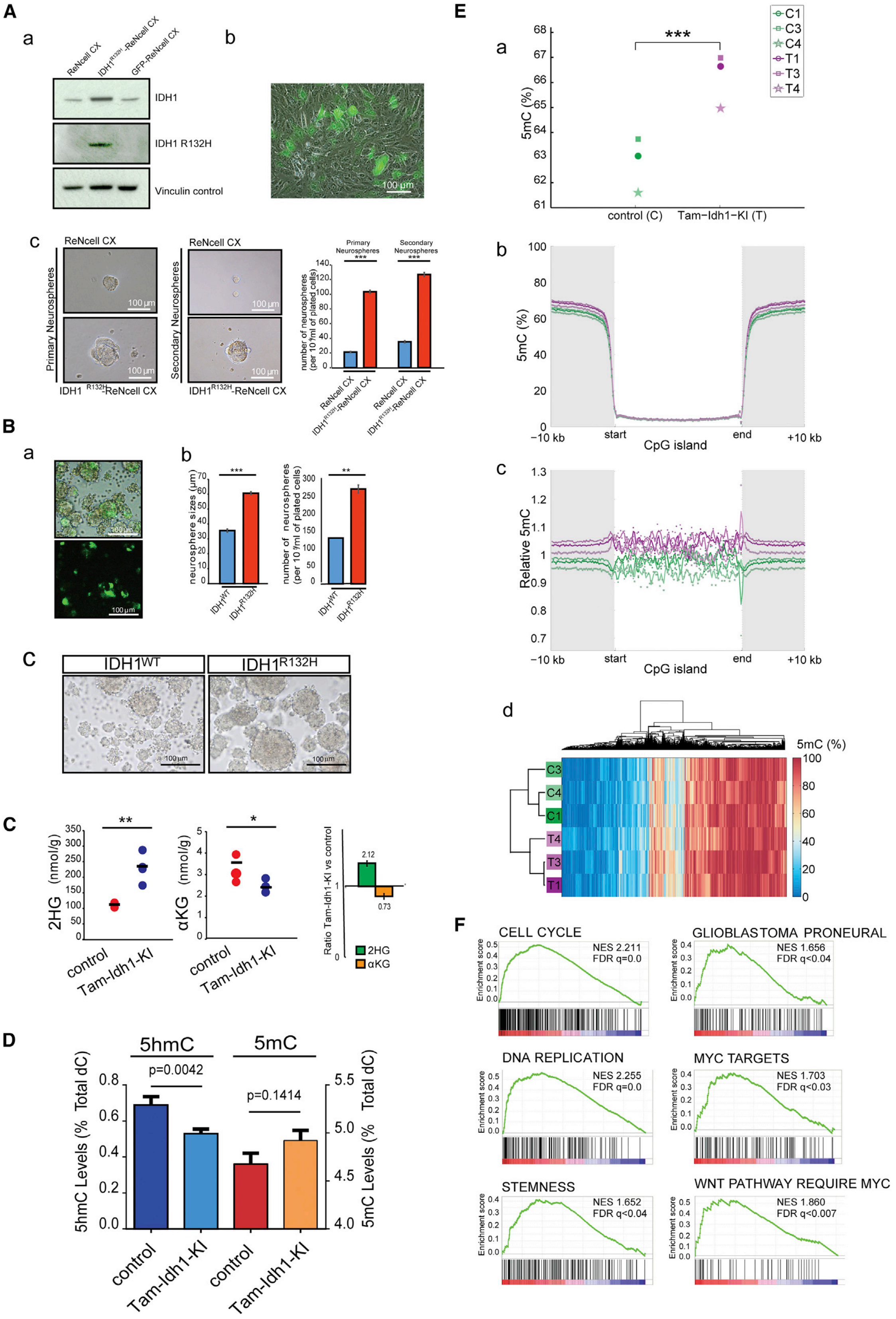


*(legend on next page)*

profiling of SVZ cells from mutant and control animals. Gene set enrichment analysis (GSEA) revealed several pathways and processes of note that were more active in Tam-Idh1-KIs than controls at a false discovery rate <0.05 (Figures 8F and S6E, Table S1). Notably, these included brain-tumor-associated processes matching the mouse phenotype such as stemness, cell cycle, maintenance of telomeres, and DNA replication. This analysis also showed significant increases in gene sets specific to human glioblastomas, including the proneural sub-type, which is characterized by mutation of *IDH1* (Verhaak et al., 2010) (Figures 8F and S6E). Interestingly, both Wnt and c-Myc target genes were also overexpressed in Tam-Idh1-KIs, suggesting potential molecular mechanisms for the gliomagenesis phenotype.

## DISCUSSION

We have shown that conditional, inducible expression of the *Idh1*$^{R132H}$ mutation in the adult mouse SVZ stem cell niche causes cellular and molecular features associated with brain tumorigenesis. We found three critical phenotypes in the SVZ niche: (1) increased numbers of label-retaining stem cells as well as acutely proliferating cells in and around the SVZ and RMS; (2) significant infiltration of neuronal and glial progenitors from the SVZ and RMS into surrounding regions; and (3) subventricular nodules containing proliferating stem/progenitor cells that protruded into the LV from the SVZ. The mutant animals developed dilation of the LVs, with accompanying distortion of the cerebrum and clinically apparent hydrocephalus. However, the brains showed little, if any, evidence of neural damage or degeneration and appeared largely normal histologically in regions away from the niche.

The phenotypic dissimilarities between our Tam-Idh1-KI and other *Idh1/2* models probably resulted from our targeting of *Idh1*$^{R132H}$ to the neurogenic niches of the adult brain. Nestin-Cre causes recombination early in the SVZ lineage and can affect large numbers of NSCs (Lagace et al., 2007). However, nestin is also expressed in other SVZ cells, including TAPs and niche astrocytes (Doetsch et al., 1997), and all of these may have been influenced by the *Idh1* mutation. Given the relatively large expansion of quiescent NSCs and the overexpression of stem cell markers in Tam-Idh1-KI mice, our data suggest that an early stem cell was targeted by Nes-Cre. The leakiness of the *Idh1*$^{fl(R132H)/+}$ allele may also have contributed to the Tam-Idh1-KI phenotype. Evidence strongly suggests that the phenotype caused by the leaky allele also originates in the SVZ. First, although the leaky allele causes 2HG accumulation in all tissues examined, no phenotype outside the brain was detected. Second, the phenotypes of Tam-Idh1-KI and leaky mice are very similar, notwithstanding much reduced penetrance and severity in the latter. Third, nestin expression is not induced by 2HG outside the SVZ and SGZ in the leaky mice; thus, while Tam-Idh1-KI mice are produced from an allele that is already leaky, when tamoxifen is given, full R132H knockin occurs in the SVZ and SGZ. Although leaky R132H expression in cells outside the SVZ could in theory explain the more numerous and misplaced cells in the striatum, corpus callosum, RMS, and OB in *Idh1* mutants, lineage tracing in Tam-Idh1-KIs showed that the cells undergoing recombination in the SVZ after tamoxifen administration could account for the great majority of these cells.

Tsc1-cKO (*Nes-Cre*$^{ER(T2)}$; *Tsc1*$^{loxP/loxP}$) mice also develop hydrocephalus, periventricular nodules, and tumor-like structures near the inter-ventricular foramen (Feliciano et al., 2012; Zhou et al., 2011). The Tsc1-KO lesions are Dcx$^{+}$ and were mostly non-proliferative, in contrast to our mice. The causes of ventricular dilation and hydrocephalus were not identified in the Tsc1-KOs, although cryptic nodules obstructing CSF flow or disrupting the ependymal lining are possible causes. Mice overexpressing *Tlx*, a key regulator of NSC expansion, also develop periventricular nodules, accompanied by SVZ and RMS changes similar to the Tam-Idh1-KIs but without the gross phenotype of our animals (Liu et al., 2010). We found increased expression of Tlx targets in the Tam-Idh1-KI mice (Table S1).

Several theories as to how the *IDH1*$^{R132H}$ mutation drives cancer were not supported by our work, including hypoxia pathway activation, increased angiogenesis, altered collagen, histone methylation, and ROS. We caution that the SVZ tissue analyzed may have contained some non-induced cells, diluting out effects. We did find that *Idh1*$^{R132H}$ animals had raised 2HG and decreased αKG, accompanied by reduced 5hmC and increased global 5mC in DNA. *IDH2* and *TET2* mutations are

**Figure 8. Assessment of Functional Mechanisms Underlying the Tam-Idh1-KI Phenotype**

(A) The experiments determined the effects of IDH1$^{R132H}$ expression in ReNcell CX neuronal progenitor cells. (a) western blot shows expression of total and mutant IDH1 in cells transduced by IDH1$^{R132H}$ or GFP lentiviral vectors compared with untransduced cells. (b) GFP expression indicates transduction efficiency. (c) Representative bright-field images are shown for ReNcell CX cells (IDH1$^{R132H}$-transduced or untransduced controls) grown as primary or secondary neurospheres at low density. The chart shows quantification of neurospheres grown at a low density. Data are representative of three independent experiments, one using high and two using low density cultures, giving consistent results. Results are presented as means ± SEM (***$p < 0.005$).

(B) Tertiary neurospheres were derived from primary SVZ cells transduced with lentiviruses expressing IDH1 wild-type (WT) or R132H. (a) GFP expression shows representative transduction efficiency. (b) After 4 days in culture, the size and number of WT and IDH1$^{R132H}$ neurospheres were measured to assess proliferation and self-renewal capacity. The chart shows a representative experiment of three independent experiments, two using high and one using low density cultures. Results are presented as means ± SEM (**$p < 0.01$, ***$p < 0.005$). (c) Representative images of IDH1-wild-type and IDH1$^{R132H}$ neurosphere cultures grown at high density are shown.

(C) Mass spectrometric assessment of the effect of *Idh1*$^{R132H}$ on 2HG and αKG levels was performed. Brain tissue extracts from Tam-Idh1-KI mice ($n = 4$) and controls ($n = 3$) aged 4–9 months were assayed for total 2HG (D and L isomers). Data are shown as dot plots and summarized as relative expression in the bar chart, presented as means ± SD. *$p < 0.05$, **$p < 0.01$, from ANOVA.

(D) Total 5hmC and 5mC levels of forebrain were assessed using denaturing HPLC. Data are presented as means ± SD.

(E) 5mC genome sequencing was performed on SVZ DNAs. (a) Levels of cytosine methylation at CpG dinucleotides in Tam-Idh1-KI (T) and controls (C) paired by age and sex are shown, ***$p = 0.0163$, from Kolmogorov-Smirnov test. (b, c) Absolute (b) and relative (c) differences in CpG methylation within CpG islands and flanking regions. (d) Hierarchical cluster analysis of top 2,000 differentially methylated CpGs to assess whether mutants and controls clustered separately.

(F) mRNA expression GSEA analysis is shown for selected gene sets enriched in Tam-Idh1-KI brains.

See also Figures S5 and S6, Table S1.

mutually exclusive in AML (Figueroa et al., 2010; Gaidzik et al., 2012), and it has been reported that expression of mutant IDH or administration of 2HG can inhibit TET2 in vitro and in vivo, resulting in decreased production of 5hmC (Figueroa et al., 2010; Sasaki et al., 2012a; Xu et al., 2011) and reduced DNA demethylation (Pastor et al., 2013). TET activity might also be limited by reduced αKG availability. The increased DNA methylation in Tam-Idh1-KI brains is correspondingly present in human *IDH1*-mutant tumors (Guilhamon et al., 2013; Lu et al., 2012; TCGA, 2015; Sturm et al., 2012), but the pattern of changes appears to differ, the former being global and relatively uniform and the latter, targeting CpG islands (CIMP). It is of note that CIMP has often been measured using microarrays that focus on CpG islands, with much less coverage of other CpGs. Therefore, some cases scored as CIMP$^+$ may actually possess a tendency to genome-wide methylation. Furthermore, a global methylator phenotype could cause CIMP by the selection of epimutations at CpG islands during tumorigenesis.

mRNA expression profiling showed Tam-Idh1-KI mice to have increased c-Myc and Wnt pathway activity, higher numbers of cycling cells, upregulated telomere maintenance, and more cells with a stem-like phenotype. Culture of human neuronal stem/progenitor cells supported the mouse data by showing increased self-renewal and proliferation in cells expressing *IDH1*$^{R132H}$. All of these functional processes are strongly mechanistically associated with tumorigenesis. Tam-Idh1-KI mice also expressed high levels of genes associated with *IDH1*-mutant human brain tumors.

Together, our findings show that expression of mutant *Idh1* recapitulates cellular and molecular features of gliomagenesis, including stem cell features, increased proliferation, infiltration into adjacent structures, and tumor-like nodules originating from nestin-expressing cells. Our data suggest that the underlying cellular defect in the Tam-Idh1-KI mice is an increased and poorly controlled number of dividing stem/progenitor cells that retain much of their differentiation capacity, thus potentially representing the earliest phase of IDH-driven gliomagenesis. Our mice provide insights into human *IDH1*-driven brain tumors and a model system for assessing therapies.

## EXPERIMENTAL PROCEDURES

Procedures are detailed in the Supplemental Experimental Procedures and summarized here. All mouse experiments were performed in accordance with institutional and national guidelines and regulations under UK Home Office Project Licence PPL 3003311. For the Tam-Idh1-KI model, mice received intraperitoneal tamoxifen injections to induce Nes-Cre-mediated recombination, causing the R132H protein to be expressed. For analysis of mRNA and proteins, snap-frozen tissue was obtained for analysis after sectioning brains and carefully removing the SVZ or other region under a dissecting microscope. For fixed tissue analysis, mice were transcardially perfused with normal saline and 4% paraformaldehyde (PFA). Brains were removed, post-fixed in 4% PFA, cryoprotected in 30% sucrose, and frozen. Sections were cut on a sliding microtome, and kept at −20°C. Sections were stained using standard free-floating IHC. Numbers of immune-positive cells were counted in confocal microscopy z stacks. All quantifications were done by an observer blinded to mouse genotypes.

Molecular assays were performed using standard protocols for (1) 5mC and 5hmC (HPLC and next-generation sequencing), (2) TCA cycle and other metabolites (mass spectrometry); and (3) mRNA expression profiling (Illumina Mouse WG6-v2 microarrays). Western blotting was performed to assess the expression of selected proteins (hypoxia, ROS, histone modification, collagen), supplemented by qRT-PCR for mRA where appropriate.

For all phenotypic assessments, including molecular studies, tamoxifen-treated, Tam-Idh1-KI animals were compared with paired, tamoxifen-treated, littermate controls (*Idh1*$^{+/+}$, with or without *Nes-Cre* transgene). Cell lineage and proliferation analysis was performed using immunofluorescence for DNA labeling agents (BrdU, EdU), proliferation marker (Ki67), and markers of differentiation (GFAP, Olig2, NeuN, Dcx, S100β, Pdgfrα). Assessment of tissue morphology was performed using H&E-stained sections, supplemented by IHC for selected markers.

Assays to assess the effect of *IDH1*$^{R132H}$ were performed by expressing the mutant protein in ReNcell CX from human fetal cerebral cortex or neural stem/progenitor cells from P4 wild-type mice. Assays performed on these cells included primary and second neurosphere formation and transwell motility.

All data are presented as means ± SDs, with nominal p values from unpaired t tests, unless specified otherwise in the figure legend.

## ACCESSION NUMBERS

Gene expression data are available at GEO: GSE85080.

## SUPPLEMENTAL INFORMATION

Supplemental Information includes Supplemental Experimental Procedures, six figures, and one table and can be found with this article online at http://dx.doi.org/10.1016/j.ccell.2016.08.017.

## AUTHOR CONTRIBUTIONS

Planned experiments: C.B., O.A.D., J.A., P.J.P., J.S.M., C.J.S., S.K., P.J.R., F.G.S., I.T. Performed experiments and analyses: C.B., O.A.D., O.A., D.K., J.A., P.J.P., K.A., I.P., L.F.M., J.S.M., H.L., S.S., N.S., R.G., T.S., O.A., P.B., M.T., F.G.S., and I.T. Wrote the manuscript: C.B., O.A.D., P.J.R., F.G.S., and I.T. Oversaw the study: C.J.S., P.M., O.A., B.S.-B., S.K., P.J.R., F.G.S., and I.T.

## ACKNOWLEDGMENTS

We acknowledge funding from: Cancer Research UK (grants A12055 and C5255/A15935) to I.T., C.J.S., P.J.R., N.S.; the Wellcome Trust (090532/Z/09/Z) to I.T.; John Fell OUP Research Fund to O.A.; and King Saud University to K.A. Nestin-Cre mice, generated by François Tronche, were provided by Axel Behrens (Cancer Research UK, London Research Institute). Nestin-Cre$^{ER(T2)}$, originally from the Eisch laboratory, and R26R-EYFP mice were provided by Xin Lu (Ludwig Institute, Oxford). ReNcell CX cells were provided by Eric O'Neill (Oxford Institute for Radiation Oncology). Lentiviral transfer and packaging vectors were from Elisa Vigna (IRCC, Turin). The following gave help and advice: Thomas Cadoux-Hudson, Nick De Pennington, Keith Morris, Wellcome Trust Centre for Human Genetics Genomics and Cellular Imaging Cores, Jerome Nicod, Carme Mont, James Hillis, Bin Sun, Martin Ducker, and Julie Davies. Patrick Pollard died during the preparation of this manuscript. We acknowledge his important contributions to cancer metabolism research.

Received: December 29, 2015
Revised: June 22, 2016
Accepted: August 29, 2016
Published: September 29, 2016

## REFERENCES

Aghili, M., Zahedi, F., and Rafiee, E. (2009). Hydroxyglutaric aciduria and malignant brain tumor: a case report and literature review. J. Neurooncol. *91*, 233–236.

Akbay, E.A., Moslehi, J., Christensen, C.L., Saha, S., Tchaicha, J.H., Ramkissoon, S.H., Stewart, K.M., Carretero, J., Kikuchi, E., Zhang, H., et al. (2014). D-2-hydroxyglutarate produced by mutant IDH2 causes cardiomyopathy and neurodegeneration in mice. Genes Dev. *28*, 479–490.

Amary, M.F., Damato, S., Halai, D., Eskandarpour, M., Berisha, F., Bonar, F., McCarthy, S., Fantin, V.R., Straley, K.S., Lobo, S., et al. (2011). Ollier disease and Maffucci syndrome are caused by somatic mosaic mutations of *IDH1* and *IDH2*. Nat. Genet. *43*, 1262–1265.

Borger, D.R., Tanabe, K.K., Fan, K.C., Lopez, H.U., Fantin, V.R., Straley, K.S., Schenkein, D.P., Hezel, A.F., Ancukiewicz, M., Liebman, H.M., et al. (2012). Frequent mutation of isocitrate dehydrogenase (IDH)1 and *IDH2* in cholangiocarcinoma identified through broad-based tumor genotyping. Oncologist *17*, 72–79.

Chowdhury, R., Yeoh, K.K., Tian, Y.M., Hillringhaus, L., Bagg, E.A., Rose, N.R., Leung, I.K., Li, X.S., Woon, E.C., Yang, M., et al. (2011). The oncometabolite 2-hydroxyglutarate inhibits histone lysine demethylases. EMBO Rep. *12*, 463–469.

Comte, I., Kim, Y., Young, C.C., van der Harg, J.M., Hockberger, P., Bolam, P.J., Poirier, F., and Szele, F.G. (2011). Galectin-3 maintains cell motility from the subventricular zone to the olfactory bulb. J. Cell Sci. *124*, 2438–2447.

Dang, L., White, D.W., Gross, S., Bennett, B.D., Bittinger, M.A., Driggers, E.M., Fantin, V.R., Jang, H.G., Jin, S., Keenan, M.C., et al. (2009). Cancer-associated *IDH1* mutations produce 2-hydroxyglutarate. Nature *462*, 739–744.

Doetsch, F., García-Verdugo, J.M., and Alvarez-Buylla, A. (1997). Cellular composition and three-dimensional organization of the subventricular germinal zone in the adult mammalian brain. J. Neurosci. *17*, 5046–5061.

Duncan, C.G., Barwick, B.G., Jin, G., Rago, C., Kapoor-Vazirani, P., Powell, D.R., Chi, J.T., Bigner, D.D., Vertino, P.M., and Yan, H. (2012). A heterozygous *IDH1* R132H/WT mutation induces genome-wide alterations in DNA methylation. Genome Res. *22*, 2339–2355.

Feliciano, D.M., Quon, J.L., Su, T., Taylor, M.M., and Bordey, A. (2012). Postnatal neurogenesis generates heterotopias, olfactory micronodules and cortical infiltration following single-cell *Tsc1* deletion. Hum. Mol. Genet. *21*, 799–810.

Figueroa, M.E., Abdel-Wahab, O., Lu, C., Ward, P.S., Patel, J., Shih, A., Li, Y., Bhagwat, N., Vasanthakumar, A., Fernandez, H.F., et al. (2010). Leukemic IDH1 and IDH2 mutations result in a hypermethylation phenotype, disrupt TET2 function, and impair hematopoietic differentiation. Cancer Cell *18*, 553–567.

Fomchenko, E.I., and Holland, E.C. (2006). Mouse models of brain tumors and their applications in preclinical trials. Clin. Cancer Res. *12*, 5288–5297.

Gaidzik, V.I., Paschka, P., Spath, D., Habdank, M., Kohne, C.H., Germing, U., von Lilienfeld-Toal, M., Held, G., Horst, H.A., Haase, D., et al. (2012). *TET2* mutations in acute myeloid leukemia (AML): results from a comprehensive genetic and clinical analysis of the AML study group. J. Clin. Oncol. *30*, 1350–1357.

Guilhamon, P., Eskandarpour, M., Halai, D., Wilson, G.A., Feber, A., Teschendorff, A.E., Gomez, V., Hergovich, A., Tirabosco, R., Fernanda Amary, M., et al. (2013). Meta-analysis of IDH-mutant cancers identifies EBF1 as an interaction partner for TET2. Nat. Commun. *4*, 2166.

Gupta, R., Webb-Myers, R., Flanagan, S., and Buckland, M.E. (2011). Isocitrate dehydrogenase mutations in diffuse gliomas: clinical and aetiological implications. J. Clin. Pathol. *64*, 835–844.

Haskins, W.E., Zablotsky, B.L., Foret, M.R., Ihrie, R.A., Alvarez-Buylla, A., Eisenman, R.N., Berger, M.S., and Lin, C.H. (2013). Molecular characteristics in MRI-classified Group 1 glioblastoma multiforme. Front. Oncol. *3*, 182.

Hirata, M., Sasaki, M., Cairns, R.A., Inoue, S., Puviindran, V., Li, W.Y., Snow, B.E., Jones, L.D., Wei, Q., Sato, S., et al. (2015). Mutant IDH is sufficient to initiate enchondromatosis in mice. Proc. Natl. Acad. Sci. USA *112*, 2829–2834.

Holland, E.C. (2001). Gliomagenesis: genetic alterations and mouse models. Nat. Rev. Genet. *2*, 120–129.

Huse, J.T., and Holland, E.C. (2009). Genetically engineered mouse models of brain cancer and the promise of preclinical testing. Brain Pathol. *19*, 132–143.

Ihrie, R.A., and Alvarez-Buylla, A. (2011). Lake-front property: a unique germinal niche by the lateral ventricles of the adult brain. Neuron *70*, 674–686.

Jablonska, B., Aguirre, A., Raymond, M., Szabo, G., Kitabatake, Y., Sailor, K.A., Ming, G.L., Song, H., and Gallo, V. (2010). Chordin-induced lineage plasticity of adult SVZ neuroblasts after demyelination. Nat. Neurosci. *13*, 541–550.

Jackson, E.L., and Alvarez-Buylla, A. (2008). Characterization of adult neural stem cells and their relation to brain tumors. Cells Tissues Organs *188*, 212–224.

Lagace, D.C., Whitman, M.C., Noonan, M.A., Ables, J.L., DeCarolis, N.A., Arguello, A.A., Donovan, M.H., Fischer, S.J., Farnbauch, L.A., Beech, R.D., et al. (2007). Dynamic contribution of nestin-expressing stem cells to adult neurogenesis. J. Neurosci. *27*, 12623–12629.

Ligon, K.L., Alberta, J.A., Kho, A.T., Weiss, J., Kwaan, M.R., Nutt, C.L., Louis, D.N., Stiles, C.D., and Rowitch, D.H. (2004). The oligodendroglial lineage marker OLIG2 is universally expressed in diffuse gliomas. J. Neuropathol. Exp. Neurol. *63*, 499–509.

Liu, H.K., Wang, Y., Belz, T., Bock, D., Takacs, A., Radlwimmer, B., Barbus, S., Reifenberger, G., Lichter, P., and Schutz, G. (2010). The nuclear receptor tailless induces long-term neural stem cell expansion and brain tumor initiation. Genes Dev. *24*, 683–695.

Lois, C., and Alvarez-Buylla, A. (1994). Long-distance neuronal migration in the adult mammalian brain. Science *264*, 1145–1148.

Losman, J.A., and Kaelin, W.G., Jr. (2013). What a difference a hydroxyl makes: mutant IDH, (R)-2-hydroxyglutarate, and cancer. Genes Dev. *27*, 836–852.

Lu, C., Ward, P.S., Kapoor, G.S., Rohle, D., Turcan, S., Abdel-Wahab, O., Edwards, C.R., Khanin, R., Figueroa, M.E., Melnick, A., et al. (2012). IDH mutation impairs histone demethylation and results in a block to cell differentiation. Nature *483*, 474–478.

Mardis, E.R., Ding, L., Dooling, D.J., Larson, D.E., McLellan, M.D., Chen, K., Koboldt, D.C., Fulton, R.S., Delehaunty, K.D., McGrath, S.D., et al. (2009). Recurring mutations found by sequencing an acute myeloid leukemia genome. N. Engl. J. Med. *361*, 1058–1066.

Moroni, I., Bugiani, M., D'Incerti, L., Maccagnano, C., Rimoldi, M., Bissola, L., Pollo, B., Finocchiaro, G., and Uziel, G. (2004). L-2-hydroxyglutaric aciduria and brain malignant tumors: a predisposing condition? Neurology *62*, 1882–1884.

Pansuriya, T.C., van Eijk, R., d'Adamo, P., van Ruler, M.A., Kuijjer, M.L., Oosting, J., Cleton-Jansen, A.M., van Oosterwijk, J.G., Verbeke, S.L., Meijer, D., et al. (2011). Somatic mosaic *IDH1* and *IDH2* mutations are associated with enchondroma and spindle cell hemangioma in Ollier disease and Maffucci syndrome. Nat. Genet. *43*, 1256–1261.

Parsons, D.W., Jones, S., Zhang, X., Lin, J.C., Leary, R.J., Angenendt, P., Mankoo, P., Carter, H., Siu, I.M., Gallia, G.L., et al. (2008). An integrated genomic analysis of human glioblastoma multiforme. Science *321*, 1807–1812.

Pastor, W.A., Aravind, L., and Rao, A. (2013). TETonic shift: biological roles of TET proteins in DNA demethylation and transcription. Nat. Rev. Mol. Cell Biol. *14*, 341–356.

Patay, Z., Mills, J.C., Lobel, U., Lambert, A., Sablauer, A., and Ellison, D.W. (2012). Cerebral neoplasms in L-2 hydroxyglutaric aciduria: 3 new cases and meta-analysis of literature data. AJNR Am. J. Neuroradiol. *33*, 940–943.

Patay, Z., Orr, B.A., Shulkin, B.L., Hwang, S.N., Ying, Y., Broniscer, A., Boop, F.A., and Ellison, D.W. (2015). Successive distinct high-grade gliomas in L-2-hydroxyglutaric aciduria. J. Inherit. Metab. Dis. *38*, 273–277.

Reitman, Z.J., and Yan, H. (2010). Isocitrate dehydrogenase 1 and 2 mutations in cancer: alterations at a crossroads of cellular metabolism. J. Natl. Cancer Inst. *102*, 932–941.

Sasaki, M., Knobbe, C.B., Itsumi, M., Elia, A.J., Harris, I.S., Chio, I.I., Cairns, R.A., McCracken, S., Wakeham, A., Haight, J., et al. (2012a). D-2-hydroxyglutarate produced by mutant IDH1 perturbs collagen maturation and basement membrane function. Genes Dev. *26*, 2038–2049.

Sasaki, M., Knobbe, C.B., Munger, J.C., Lind, E.F., Brenner, D., Brustle, A., Harris, I.S., Holmes, R., Wakeham, A., Haight, J., et al. (2012b). *IDH1*(R132H) mutation increases murine haematopoietic progenitors and alters epigenetics. Nature *488*, 656–659.

Singh, S.K., Clarke, I.D., Hide, T., and Dirks, P.B. (2004). Cancer stem cells in nervous system tumors. Oncogene *23*, 7267–7273.

Srinivas, S., Watanabe, T., Lin, C.S., William, C.M., Tanabe, Y., Jessell, T.M., and Costantini, F. (2001). Cre reporter strains produced by targeted insertion of EYFP and ECFP into the ROSA26 locus. BMC Dev. Biol. *1*, 4.

Sturm, D., Witt, H., Hovestadt, V., Khuong-Quang, D.A., Jones, D.T., Konermann, C., Pfaff, E., Tonjes, M., Sill, M., Bender, S., et al. (2012). Hotspot mutations in *H3F3A* and *IDH1* define distinct epigenetic and biological subgroups of glioblastoma. Cancer Cell *22*, 425–437.

Suzuki, A., Nobusawa, S., Natsume, A., Suzuki, H., Kim, Y.H., Yokoo, H., Nagaishi, M., Ikota, H., Nakazawa, T., Wakabayashi, T., et al. (2014). Olig2 labeling index is correlated with histological and molecular classifications in low-grade diffuse gliomas. J. Neurooncol. *120*, 283–291.

Tarhonskaya, H., Rydzik, A.M., Leung, I.K., Loik, N.D., Chan, M.C., Kawamura, A., McCullagh, J.S., Claridge, T.D., Flashman, E., and Schofield, C.J. (2014). Non-enzymatic chemistry enables 2-hydroxyglutarate-mediated activation of 2-oxoglutarate oxygenases. Nat. Commun. *5*, 3423.

TCGA. (2015). The molecular taxonomy of primary prostate cancer. Cell *163*, 1011–1025.

Tronche, F., Kellendonk, C., Kretz, O., Gass, P., Anlag, K., Orban, P.C., Bock, R., Klein, R., and Schutz, G. (1999). Disruption of the glucocorticoid receptor gene in the nervous system results in reduced anxiety. Nat. Genet. *23*, 99–103.

Verhaak, R.G., Hoadley, K.A., Purdom, E., Wang, V., Qi, Y., Wilkerson, M.D., Miller, C.R., Ding, L., Golub, T., Mesirov, J.P., et al. (2010). Integrated genomic analysis identifies clinically relevant subtypes of glioblastoma characterized by abnormalities in PDGFRA, IDH1, EGFR, and NF1. Cancer Cell *17*, 98–110.

Xu, W., Yang, H., Liu, Y., Yang, Y., Wang, P., Kim, S.H., Ito, S., Yang, C., Wang, P., Xiao, M.T., et al. (2011). Oncometabolite 2-hydroxyglutarate is a competitive inhibitor of alpha-ketoglutarate-dependent dioxygenases. Cancer Cell *19*, 17–30.

Yan, H., Parsons, D.W., Jin, G., McLendon, R., Rasheed, B.A., Yuan, W., Kos, I., Batinic-Haberle, I., Jones, S., Riggins, G.J., et al. (2009). *IDH1* and *IDH2* mutations in gliomas. N. Engl. J. Med. *360*, 765–773.

Zhao, S., Lin, Y., Xu, W., Jiang, W., Zha, Z., Wang, P., Yu, W., Li, Z., Gong, L., Peng, Y., et al. (2009). Glioma-derived mutations in *IDH1* dominantly inhibit IDH1 catalytic activity and induce HIF-1alpha. Science *324*, 261–265.

Zhou, J., Shrikhande, G., Xu, J., McKay, R.M., Burns, D.K., Johnson, J.E., and Parada, L.F. (2011). *Tsc1* mutant neural stem/progenitor cells exhibit migration deficits and give rise to subependymal lesions in the lateral ventricle. Genes Dev. *25*, 1595–1600.

Cancer Cell, Volume *30*

## Supplemental Information

# Expression of *Idh1*$^{R132H}$ in the Murine Subventricular Zone Stem Cell Niche Recapitulates Features of Early Gliomagenesis

**Chiara Bardella, Osama Al-Dalahmah, Daniel Krell, Pijus Brazauskas, Khalid Al-Qahtani, Marketa Tomkova, Julie Adam, Sébastien Serres, Helen Lockstone, Luke Freeman-Mills, Inga Pfeffer, Nicola Sibson, Robert Goldin, Benjamin Schuster-Böeckler, Patrick J. Pollard, Tomoyoshi Soga, James S. McCullagh, Christopher J. Schofield, Paul Mulholland, Olaf Ansorge, Skirmantas Kriaucionis, Peter J. Ratcliffe, Francis G. Szele, and Ian Tomlinson**

**SUPPLEMENTAL DATA**

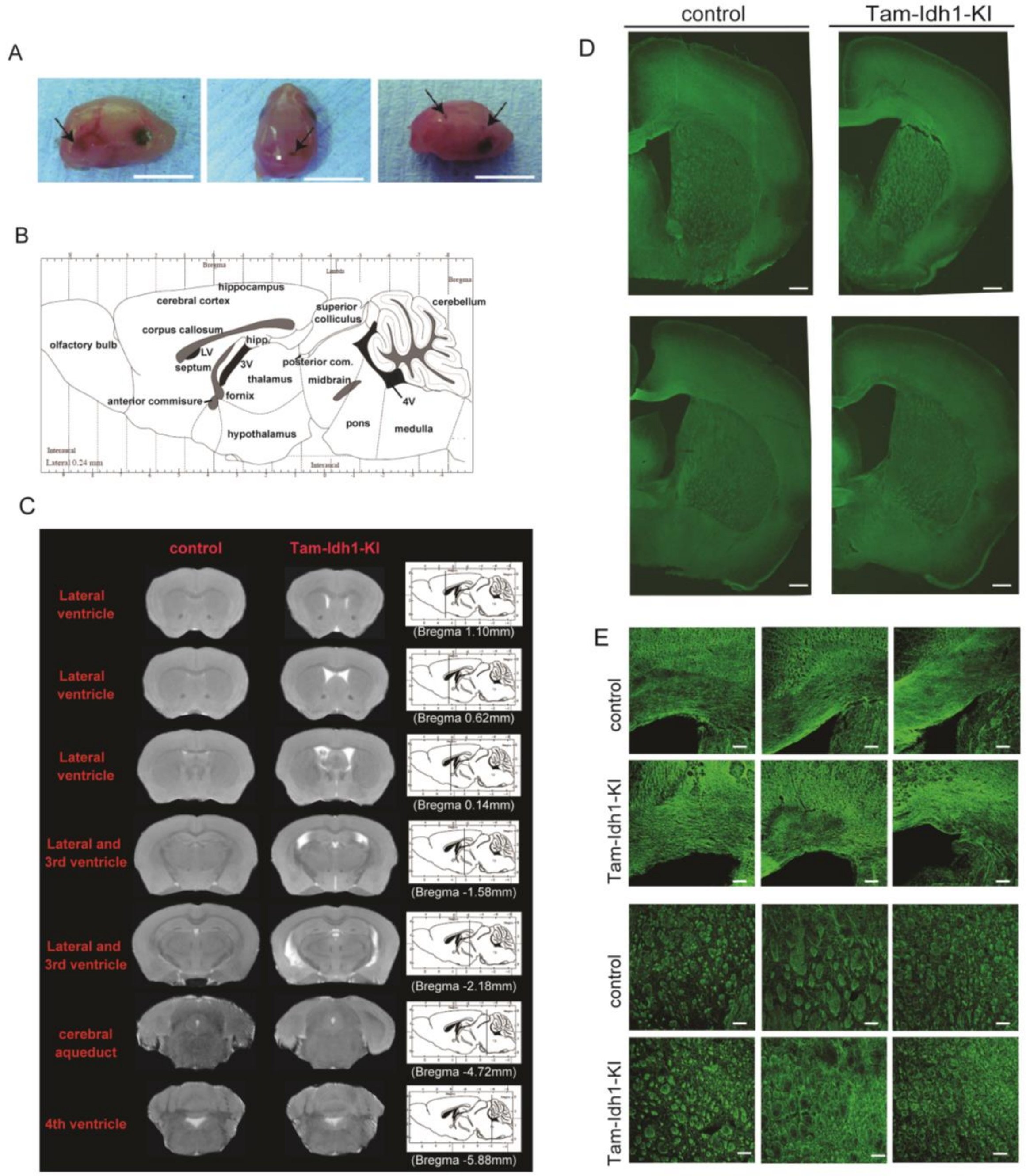

**Figure S1 related to Figure 1: Phenotypes caused by expression of *Idh1*$^{R132H}$ in the embryonic and adult brain.**

A. Dorsal and lateral views of embryonic mouse heads dissected from Idh1-KI animals.
Areas of hemorrhage in these animals, derived from an *Idh1*$^{fl(R132H)/+}$ *x* Nes-Cre cross, are shown (black arrows). Scale bars 5 mm.

B. Schematic of sagittal section of mouse brain.
A section of the mouse brain is taken in sagittal section 0.24 mm lateral to the midline. Major structures are indicated. Ventricles are shown in black. Modified from "The Mouse Brain" by G. Paxinos and K.J.B Franklin (Elsevier, 2015, ISBN: 978-0-12-391057-8).

C. Sequential coronal images of MRI scans of Tam-Idh1-KI (adult) and control brains.
The images shown are at comparable positions from Tam-Idh1-KI and control mice. Bregma coordinates for each MRI image and the corresponding sagittal position are shown with vertical lines.

D. Myelin basic protein (MBP) in coronal Tam-Idh1-KI brain sections compared with controls using immunofluorescence. MBP is a marker of mature oligodendrocytes and brain damage. Scale bars are 500 µm.

E. MBP expression in the corpus callosum (middle panels) and striatum (bottom panels) of Tam-Idh1-KI mice and controls. Scale bars 100 µm.

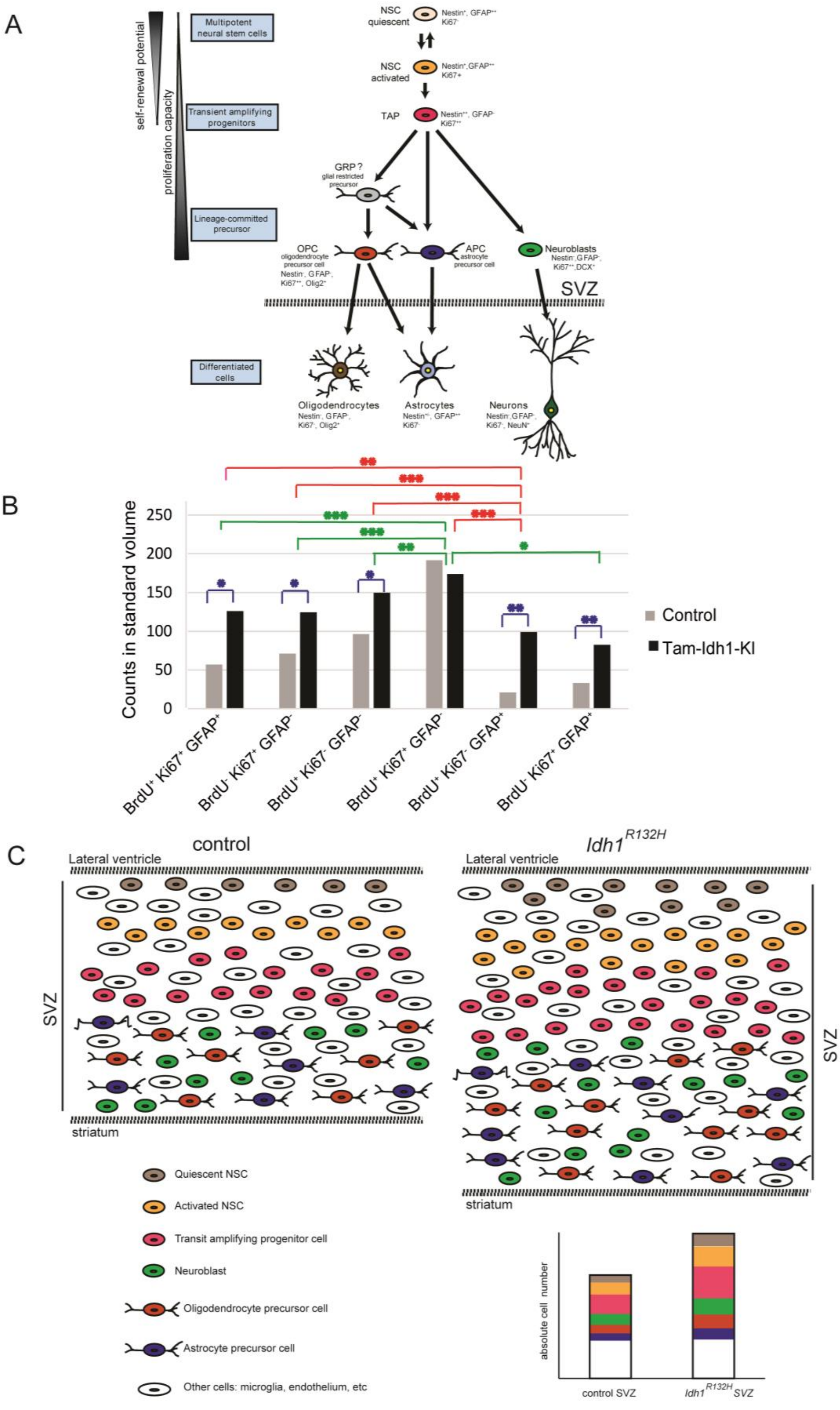
A
self-renewal potential
proliferation capacity
Multipotent neural stem cells
Transient amplifying progenitors
Lineage-committed precursor
Differentiated cells
NSC quiescent
Nestin+, GFAP++ Ki67-
NSC activated
Nestin+, GFAP++ Ki67+
TAP
Nestin++, GFAP- Ki67++
GRP ? glial restricted precursor
OPC oligodendrocyte precursor cell
Nestin-, GFAP-, Ki67++, Olig2+
APC astrocyte precursor cell
Neuroblasts
Nestin-, GFAP-, Ki67++, DCX+
SVZ
Oligodendrocytes
Nestin-, GFAP-, Ki67-, Olig2+
Astrocytes
Nestin+-, GFAP++ Ki67-
Neurons
Nestin-, GFAP-, Ki67-, NeuN+
B
Counts in standard volume
250
200
150
100
50
0
Control
Tam-Idh1-KI
BrdU+ Ki67+ GFAP+
BrdU- Ki67+ GFAP-
BrdU+ Ki67- GFAP-
BrdU+ Ki67+ GFAP-
BrdU+ Ki67- GFAP+
BrdU- Ki67+ GFAP+
C
control
Idh1 R132H
Lateral ventricle
SVZ
striatum
Quiescent NSC
Activated NSC
Transit amplifying progenitor cell
Neuroblast
Oligodendrocyte precursor cell
Astrocyte precursor cell
Other cells: microglia, endothelium, etc
absolute cell number
control SVZ
Idh1 R132H SVZ

**Figure S2 related to Figure 3. Canonical model of the adult mouse SVZ cell lineages and detailed analysis of lineage changes in the SVZ of Tam-Idh1-KI mice.**

A. The SVZ is well characterized and has become a major system for studying mechanisms of neural development and tumorigenesis. Slowly dividing NSCs give rise to frequently dividing transit amplifying progenitor cells (TAPs), which in turn generate lineage-committed immature neuroblasts or glioblasts (OPCs and APCs). SVZ "niche astrocytes" (not shown) do not exhibit stem cell features and physically separate neuroblasts from the surrounding parenchyma. Ependymal cells (not shown) line the lateral ventricles, forming the cerebrospinal fluid–brain barrier, and surround apical primary cilia of NSCs (Doetsch et al., 1999; Mamber et al., 2013; Mirzadeh et al., 2008; Sajad et al., 2011; Silva-Vargas et al., 2013). The neuroblasts move in the rostral migratory stream (RMS) from the SVZ to the olfactory bulbs (OBs), where they differentiate into functional interneurons (Lois and Alvarez-Buylla, 1994). In addition to neurons, the adult SVZ also produces a small number of astrocytes and oligodendrocytes, the latter via resident oligodendrocyte progenitor cells. Neurogenesis in the adult human SVZ has been controversial (Curtis et al., 2007; Sanai et al., 2011), but was recently confirmed by a series of retrospective analyses of nuclear bomb test [$^{14}$C]-labelling of adult-born human neurons (Ernst et al., 2014). Based on these data, it is likely the human SVZ generates cells that constitutively migrate to the adjacent striatum rather than to the OB as in rodents (Ernst et al., 2014). This makes it more likely that tumors derived from SVZ cells also infiltrate structures adjacent to the lateral ventricle, such as the striatum.

B. We postulated that the *Idh1*$^{R132H}$ mutation could act in two ways: (i) changing total numbers of cells; and/or (ii) changing the proportions of each cell type. Importantly, SVZ volume was larger in mutants, whereas overall cellular density was not significantly changed. Total SVZ cell numbers were therefore increased in the mutants. It also follows that the density of any cell type can be used as a measure of its proportion in the cell population, but *not* as a measure of total numbers of those cells. More specifically, cell types with increased density in mutants must also have increased total numbers, but cells with decreased density may have lower, unchanged or somewhat increased numbers (because the size of the expanded SVZ must be taken into account). Although we could not simultaneously assess large numbers of cell lineage markers using IF, we were able to count cells for 6 of the possible 8 combinations of BrdU, Ki67 and GFAP:

BrdU$^+$/Ki67$^+$/GFAP$^+$ - label-retaining GFAP$^+$ cells that had re-entered the cycle, most likely quiescent NSCs becoming activated NSCs;

BrdU$^-$/Ki67$^+$/GFAP$^-$ - one or more of TAPs, oligodendrocyte precursors and neuroblasts;

BrdU$^+$/Ki67$^-$/GFAP$^-$ - "other" stem-like or slowly dividing cells of uncertain type;

BrdU$^+$/Ki67$^+$/GFAP$^-$ - other label-retaining cells of uncertain type, such as endothelial cells or microglia, which had re-entered the cell cycle;

BrdU$^+$/Ki67$^-$/GFAP$^+$ - probably quiescent NSCs;

BrdU$^-$/Ki67$^+$/GFAP$^+$ - activated NSCs, or possibly cycling niche astrocytes (but not ependymal cells as these were Ki67$^-$); and

BrdU$^-$/Ki67$^-$/GFAP$^-$ and BrdU$^-$/Ki67$^-$/GFAP$^+$ - could not be assessed for technical reasons.

Some of the above counts were obtained indirectly (e.g. BrdU$^+$/Ki67$^-$/GFAP$^+$ was calculated by subtracting BrdU$^+$/Ki67$^+$/GFAP$^+$ counts from BrdU$^+$/GFAP$^+$ counts). Significant differences in the proportions of cells in Tam-Idh1-KIs and controls are shown in blue (*p<0.05). We then assessed whether each cell type was affected to a similar extent by the *Idh1*$^{R132H}$ mutation. For this, we used a contingency table approach, in which columns contained each of the 6 cell types, and rows comprised mutant or wildtype mice. To obtain standardized data, we rebased cell type counts for each mouse to an SVZ volume of 750,000 µm$^3$ (as this was the smallest SVZ volume assessed in any animal). With the caveat that BrdU$^-$/Ki67$^-$/GFAP$^+$ and BrdU$^-$/Ki67$^-$/GFAP$^-$ populations could not be assessed in this experiment, pairwise assessments of each of the 6 cell types were performed to determine which populations were differentially affected by the *Idh1* mutation (significant results shown in red and green; *p<0.05, **p<0.01, ***p<0.005).

C. A schematic summary of the SVZ cell proliferation and lineage analysis is shown. Numbers and proportions of quiescent NSCs, activated NSCs, TAPs, oligodendrocyte precursors, neuroblasts and putative astrocyte precursors (shown in colors) were directly assessed. Changes in the density/proportion and numbers of other cell types (shown in white) were deduced from measurements of total cell numbers, although the identities of these cells are not well defined. For simplicity, we have not reproduced the complex cytoarchitectural arrangement of SVZ cell types. The bar chart denotes the absolute numbers of each cell type in the SVZs of control and Tam-Idh1-KI mice.

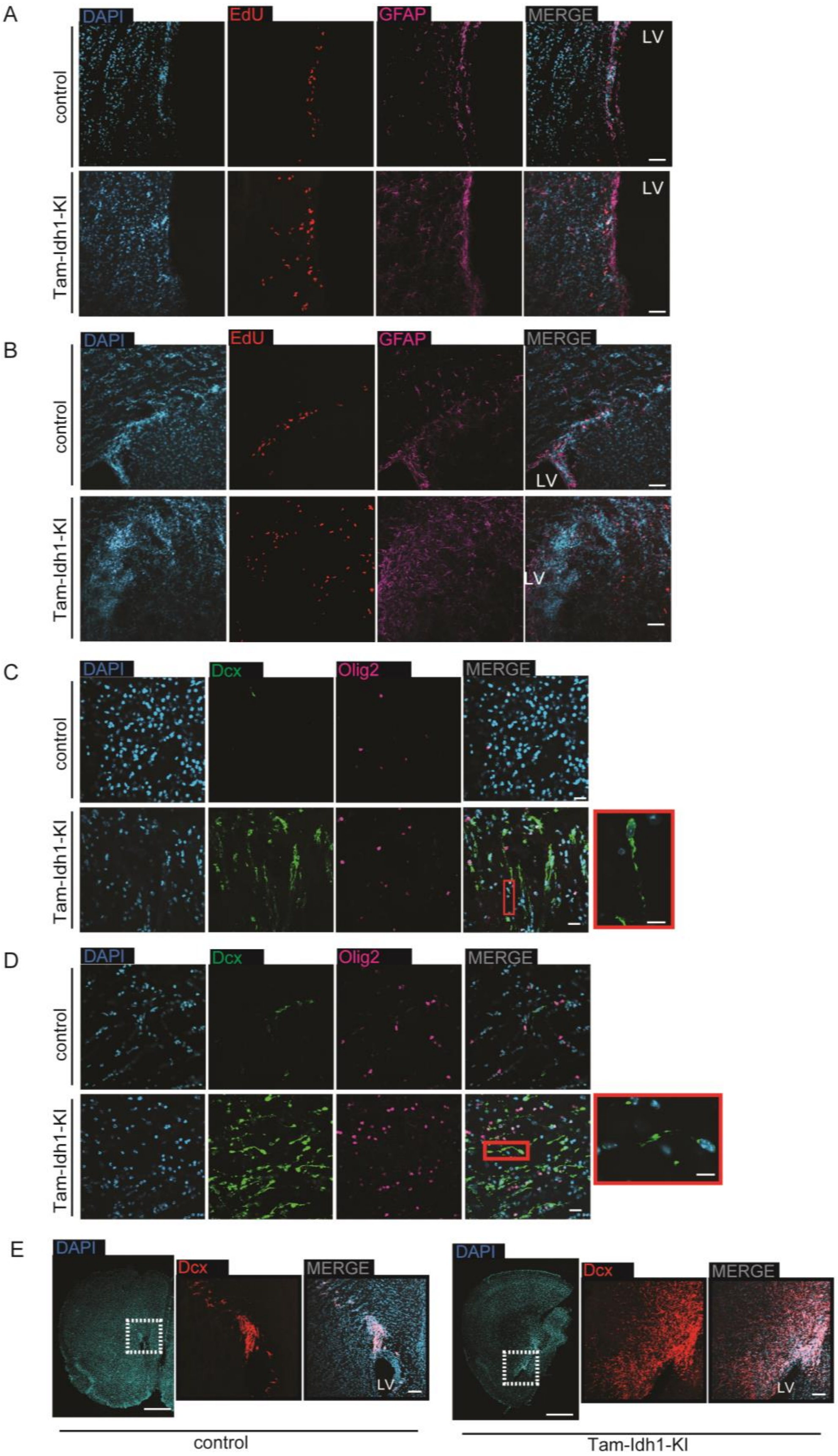
A
DAPI
EdU
GFAP
MERGE
LV
control
Tam-Idh1-KI
B
C
Dcx
Olig2
D
E
control
Tam-Idh1-KI

**Figure S3 related to Figure 4. Further cell lineage analysis in tissues surrounding the SVZ.**

A. Ectopic proliferating ($EdU^+$) cells of the astrocyte lineage ($GFAP^+$) in the striatum of Tam-Idh1-KI mice compared with controls.
LV: lateral ventricles. The area within the red box is also shown magnified. Scale bars 50 µm. Sagittal sections.

B. Ectopic proliferating ($EdU^+$) cells of the astrocyte lineage ($GFAP^+$) in the corpus callosum of Tam-Idh1-KI mice compared with controls.
Annotation is as per (A).

C. Immature neuroblasts ($Dcx^+$) and oligodendrocytes ($Olig2^+$) in the striatum of Tam-Idh1-KI mice compared with controls.
Scale bars 20 µm. In the red squares, $Dcx^+$ cells, marking migratory neuroblasts with extended leading processes are shown in normal and magnified views. Scale bars 10 µm. Sagittal sections.

D. Immature neuroblasts ($Dcx^+$) and oligodendrocytes ($Olig2^+$) in the corpus callosum of Tam-Idh1-KI mice compared with controls.
Annotation is as per (C).

E. Neuroblasts emigrating from the dorsolateral corner of the SVZ in Tam-Idh1-KI brains compared with controls.
Areas in the inset are magnified. LV: lateral ventricle. Scale bars 1.5 mm and 100 µm. Coronal sections.

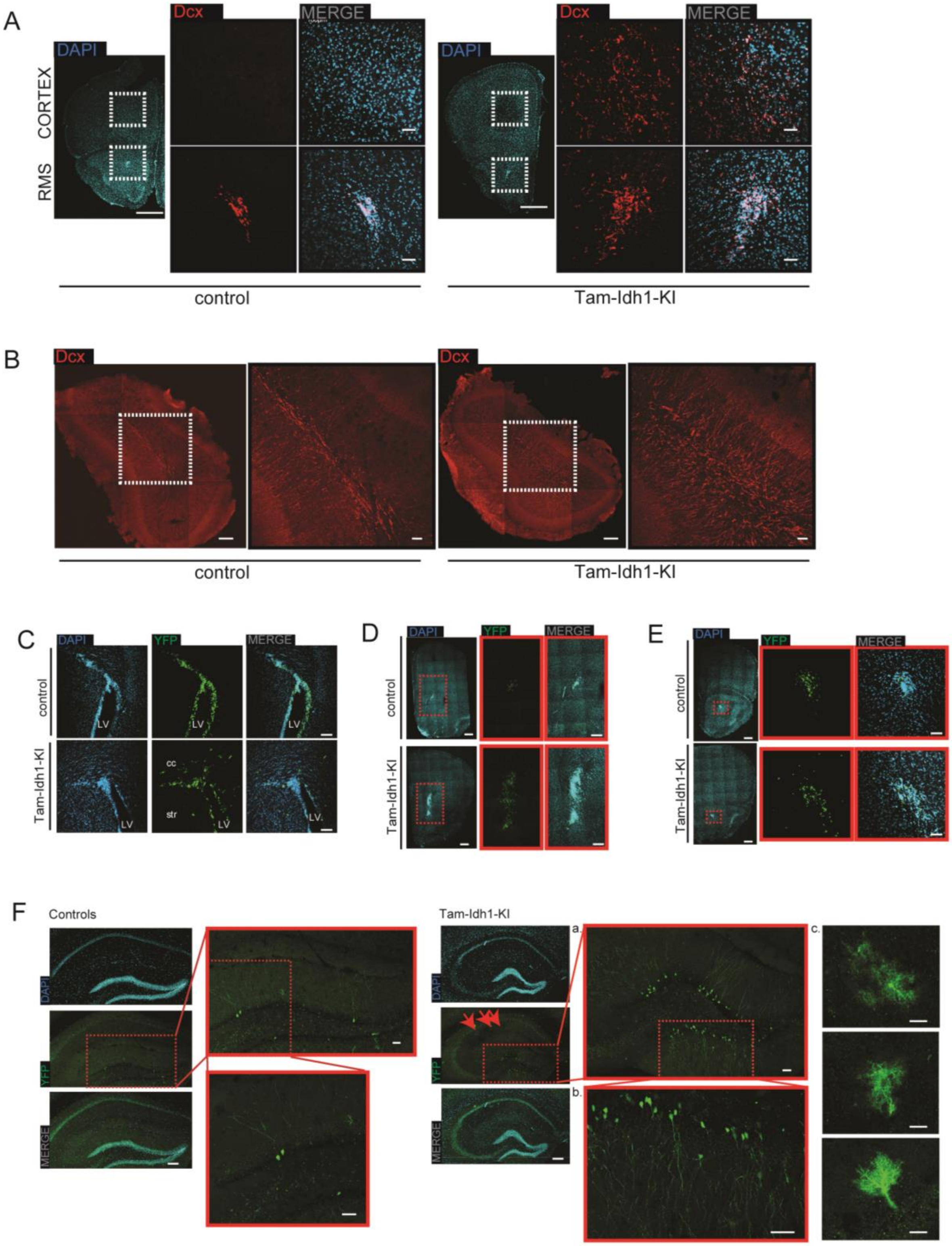
A
DAPI
Dcx
MERGE
CORTEX
RMS
control
Tam-Idh1-KI
B
Dcx
control
Tam-Idh1-KI
C
DAPI
YFP
MERGE
control
Tam-Idh1-KI
LV
cc
str
D
E
F
Controls
Tam-Idh1-KI
a.
b.
c.

**Figure S4 related to Figure 5: Ectopic cell migration.**

A. Neuroblasts migrating outside the RMS in Tam-Idh1-KI mice compared with controls.
Areas in the inset are magnified. Scale bars 1.5 mm (DAPI) and 100 µm (Dcx and MERGE).

B. Diffuse migration of neuroblasts outside the RMS in the OB of Tam-Idh1-KI mice compared with controls.
Once migrated into the core of the OB, $Dcx^+$ neuroblasts normally detach from the RMS, and move radially into the granular and glomerular OB layers, where they differentiate into subtypes of $NeuN^+$ interneurons (Ihrie and Alvarez-Buylla, 2011). Here we assess whether *Idh1*$^{R132H}$ may have caused a redirection of SVZ progenitors from the OB to ectopic periventricular regions or may have inhibited normal SVZ neurogenic differentiation. Areas in the inset are magnified. Scale bars 200 µm and 50 µm (magnified).

C. $YFP^+$ cells emigrating outside the dorsolateral corner of the SVZ in Tam-Idh1-KI reporter (tamoxifen-treated *Nes-Cre*$^{ER(T2)}$*;Idh1*$^{R132H/+}$*;R26R-EYFP*) mice compared with control reporter mice (*Nes-CreER*$^{(T2)}$*;Idh1*$^{+/+}$*;R26R-EYFP*).
LV: lateral ventricle; str: striatum; cc: corpus callosum. Scale bars 100 µm,

D. Migratory stream in the proximal RMS in Tam-Idh1-KI and control YFP reporter mice.
Areas in the inset are magnified. Scale bars 100 µm in A, 400 µm and 200 µm in B, and 400 µm and 50 µm in C.

E. Diffuse migratory stream in the main RMS in Tam-Idh1-KI reporter mice.
Areas in the inset are magnified. Scale bars 100 µm in A., 400 µm and 200 µm in B., and 400 µm and 50 µm in C..

F. $YFP^+$ cells in the hippocampal dentate gyrus (HDG) and cornu ammonis of Tam-Idh1-KI and control reporter mice.
In addition to the SVZ, the other major adult neurogenic niche is the SGZ of the HDG. Areas in the insets are magnified in (a) and (b). Scale bars 50 µm. The cornu ammonis region of the hippocampus, which is normally non-neurogenic, is indicated by red arrows and shown magnified in (c), scale bars 20 µm.

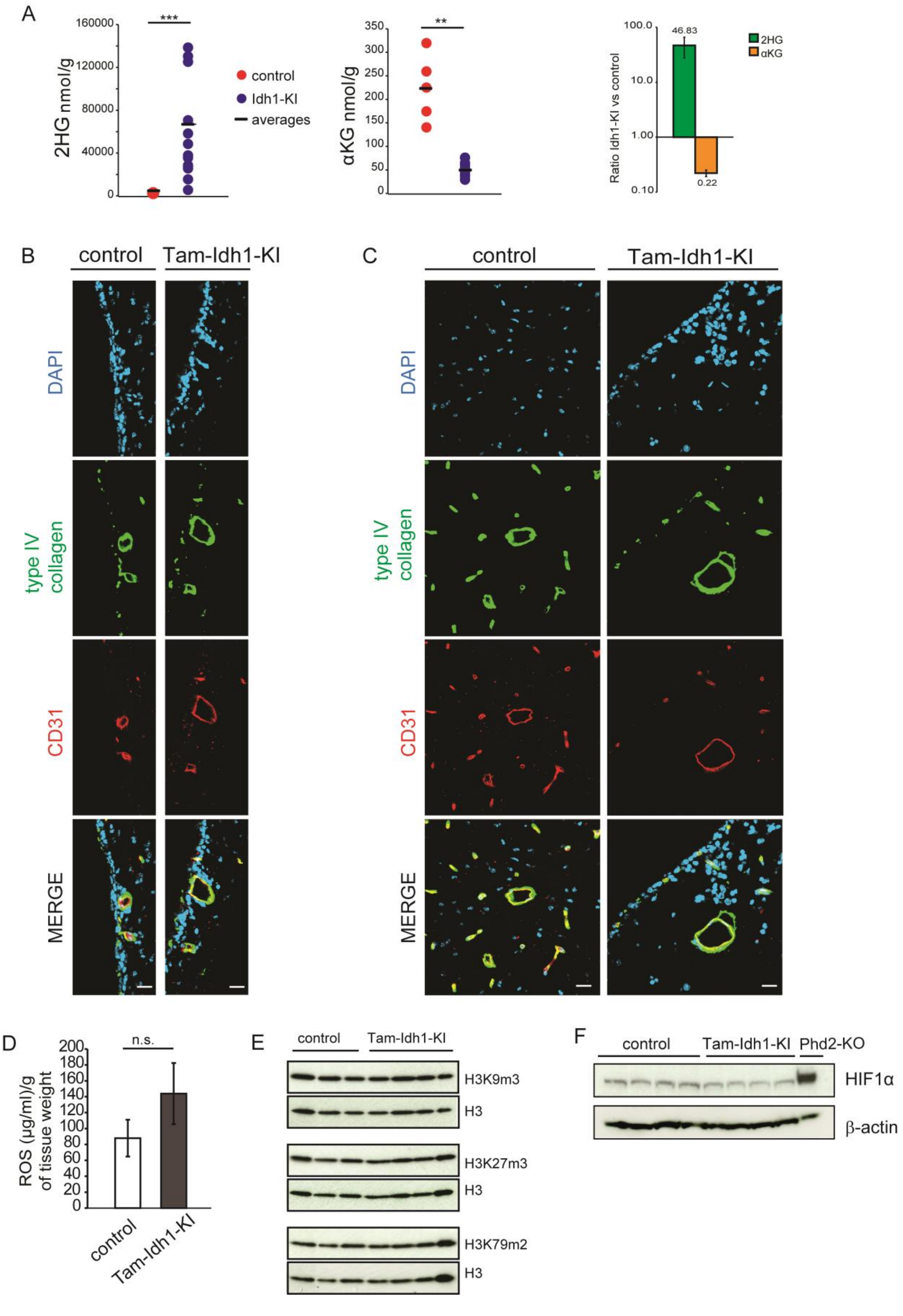
A
2HG nmol/g
160000
120000
80000
40000
0
***
control
Idh1-KI
averages
αKG nmol/g
350
300
250
200
150
100
50
0
**
Ratio Idh1-KI vs control
100.0
10.0
1.00
0.10
46.83
0.22
2HG
αKG
B
control
Tam-Idh1-KI
DAPI
type IV collagen
CD31
MERGE
C
control
Tam-Idh1-KI
DAPI
type IV collagen
CD31
MERGE
D
ROS (μg/ml)/g of tissue weight
200
180
160
140
120
100
80
60
40
20
0
n.s.
control
Tam-Idh1-KI
E
control
Tam-Idh1-KI
H3K9m3
H3
H3K27m3
H3
H3K79m2
H3
F
control
Tam-Idh1-KI
Phd2-KO
HIF1α
β-actin

**Figure S5 related to Figure 8: Collagen expression, blood vessel morphology, ROS levels, histone methylation and hypoxia pathway in Tam-Idh1-KIs and controls.**

A. Metabolic analysis of brain tissue from Idh1-KI and control embryos.
Whole brains from Idh1-KI (n=16) and control embryos (n=6) at E16-17 were dissociated to a single cell suspension and we performed metabolic profiling using ion chromatography-mass spectrometry (IC-MS). The bar chart shows the data normalized to the average values measured in control brains. Data are presented as mean ± SD. **$p<0.01$, ***$p<0.005$.

B. Type IV collagen expression and blood vessel morphology in the SVZ of adult Tam-Idh1-KI brains compared with controls.
Sections from our mice were immunolabeled with type IV collagen and CD31 antibodies (scale bars 20 µm) to identify blood vessels.

C. Analysis of the adult brain parenchyma for collagen and blood vessel abnormalities in adult Tam-Idh1-KI brains compared with controls.
Details are as for B.

D. ROS levels in the adult brain of adult Tam-Idh1-KI brains and to controls.
Brains from Tam-Idh1-KI (n=3) and control (n=3) mice were analyzed. The difference in levels was not significant ($p>0.05$)

E. Histone methylation in the adult brain.
H3K9m3, H3K27m3 and H3K79m2 were assessed separately using Western blotting. Each blot was probed separately for total H3 as a comparison.

F. Hypoxia pathway in the adult brain.
Western blot for HIF1α was performed on forebrains of Tam-Idh1-KI and control mice. The same blot was probed separately for β-actin as a loading control. Phd2-null fibroblasts were a positive control.

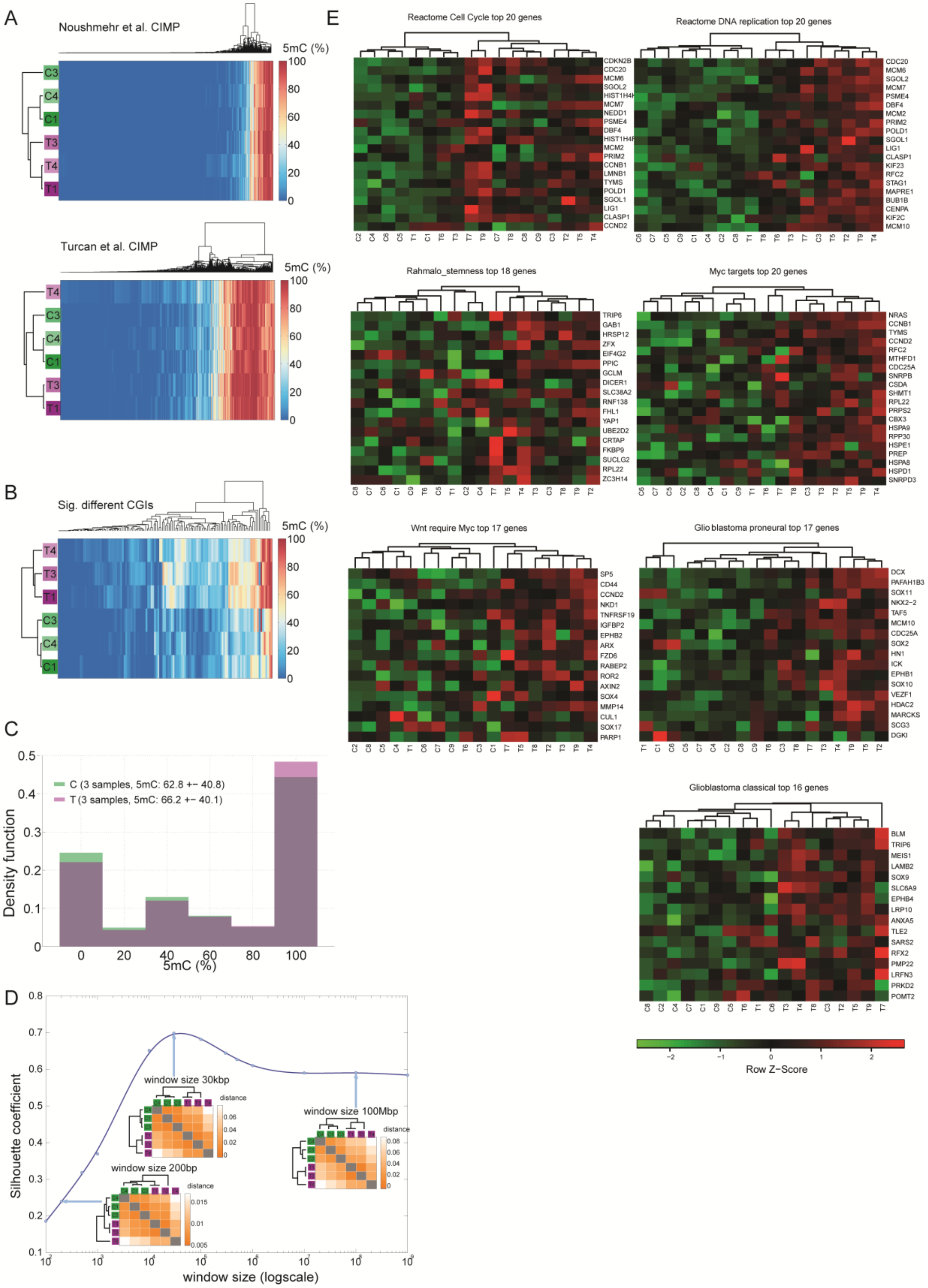

A
Noushmehr et al. CIMP
5mC (%)
C3
C4
C1
T3
T4
T1
Turcan et al. CIMP
B
Sig. different CGIs
C
C (3 samples, 5mC: 62.8 +− 40.8)
T (3 samples, 5mC: 66.2 +− 40.1)
Density function
5mC (%)
D
Silhouette coefficient
window size 30kbp
window size 100Mbp
window size 200bp
distance
window size (logscale)
E
Reactome Cell Cycle top 20 genes
Reactome DNA replication top 20 genes
Rahmalo_stemness top 18 genes
Myc targets top 20 genes
Wnt require Myc top 17 genes
Glio blastoma proneural top 17 genes
Glioblastoma classical top 16 genes
Row Z−Score

**Figure S6 related to Figure 8: Further details of the OxBSseq for 5mC and of gene expression analysis.**

A. Hierarchical clustering of methylated CpGs for genes postulated as targeted by the CpG island methylator phenotype in human glioma by other groups (Noushmehr et al., 2010; Turcan et al., 2012).

B. Hierarchical clustering of significantly methylated CpGs in our data.
Significance was assessed using Fisher's exact test (after Bonferroni correction of p values).

C. Density plot of methylation level at CpGs genome-wide in our data (proportion of Cs methylated) in Tam-Idh1-KI (T) animals and control (C) animals. The large proportion of completely methylated and completely unmethylated sites is likely to represent both a true biological phenomenon and an effect of some single or low coverage sites.

D. Silhouette analysis to determine the methylation window size that discriminates most between Tam-Idh1-KI and control mice.

E. Heat maps of the genes showing largest differences in mRNA expression for selected pathways identified using GSEA analysis.
The pathways represented were chosen as strong candidates for involvement in gliomagenesis that were significantly different, after correction for multiple testing, between Tam-Idh1-KI (T1-9) and wildtype mice (C1-9). Unsupervised hierarchical clustering was used to group mice, with the purpose of assessing a tendency for Tam-Idh1-KI and wildtype mice to cluster separately.

**Table S1 related to Figure 8. Gene set enrichment analysis of mRNA expression profiling in Tam-Idh1-KI and control SVZs. Provided as an Excel file.**

SIZE: no. of genes in set; ES: enrichment score; NES: normalized enrichment score; NOM p val: nominal p value; FDR q val: Benjamini-Hochberg false discovery rate; FWER p val: family-wise error rate p value; RANK AT MAX: position in the ranked list at which the maximum enrichment score occurred; LEADING EDGE: results are explained in detail at http://software.broadinstitute.org/gsea/doc/GSEAUserGuideFrame.html/.

Gene sets concordant with Tam-Idh1-KI at FDR<0.05 or discordant at FDR<0.01 are shown. Sets with putative importance for gliomagenesis are in bold. These include cell cycle components, DNA replication, telomere maintenance, Myc targets, Wnt targets, Tlx targets, stem cell behavior and human glioblastomas.

## SUPPLEMENTAL EXPERIMENTAL PROCEDURES

### Generation of mice

We designed a targeting construct for replacement of the endogenous *Idh1* gene in ES cells, comprising a 5' homology arm (~4.0 kb), a conditional knock-in region (~2.5 kb), a 3' homology arm (~3.9 kb) that included the R132H mutation, and a DTA expression cassette. The knock-in region comprised a 5' loxP site, a wildtype mini-gene (exons 3-9 and 3'UTR), an SV40 polyA signal, a $Neo^R$ cassette flanked by Frt sites, and a 3' LoxP site (Figure 1). These fragments were sequentially cloned into the LoxFtNwCD vector and were confirmed by restriction digestion and end-sequencing. NotI was used to linearize the final vector prior to electroporation into C57BL/6 ES cells, which were selected with 200 µg/ml G418. The construct was incorporated by homologous recombination into C57BL/6N ES cells. After PCR-based screening and R132H mutation sequencing, 3 clones carrying the R132H mutation were expanded for further analysis. After additional Southern and PCR/sequencing confirmation analysis two clones were confirmed to be correctly targeted. These cells were injected into a C57BL/6J-tyr albino blastocyst donor strain, and, after confirming germline transmission of the mutant allele, we maintained the resulting $Idh1^{fl(R132H)/+}$ animals on the C57BL/6 background. We subsequently used Cre-mediated recombination to remove the $Neo^R$ cassette and knock-in the R132H allele *in vivo*. Other mice were provided by collaborators or from public sources. All mouse procedures were carried out in accordance to Home Office UK regulations and the Animals (Scientific Procedures) Act 1986. All mice were housed at the animal unit at Functional Genomics Facility, Wellcome Trust Centre for Human Genetics, Oxford University.

### Excision of the $Neo^R$ cassette

In order to remove the Frt-flanked $Neo^R$ cassette, we crossed $Idh1^{fl(R132H)/+}$ animals with homozygous FLPe mice. These crosses, however, repeatedly failed to produce *Idh1*-mutant offspring, and FLPe x $Idh1^{fl(R132H)/fl(R132H)}$ crosses produced no offspring at all. Since construct design meant that Cre-mediated recombination would both remove the $Neo^R$ cassette and lead to knock-in of the R132H allele, we decided to retain the $Neo^R$ until the time of mutation knock-in using constitutive or tamoxifen-inducible Nestin-Cre.

### Tamoxifen treatment and DNA labelling

To create the Tam-Idh1-KO model, $Nes\text{-}Cre^{ER(T2;)}Idh1^{fl(R132H)/+}$ animals received intraperitoneal (i.p.) tamoxifen injections at 5 or 6 weeks of age. For labelling studies, 5-Bromo-2′-deoxyuridine (BrdU) (Sigma Aldrich) and 5-ethynyl-2′-deoxyuridine (EdU) (Life Technologies) were dissolved in sterile normal saline (10 mg/ml) and injected i.p. 18-28 weeks after tamoxifen (3 daily single injections of BrdU at 50 mg/kg and 13 days later, 2 hours before sacrifice, one dose of EdU at 50 mg/kg).

### Crosses with reporter R26R-EYFP mice

To verify that recombination of the *Idh1* conditional allele had been targeted in cells that specifically express *Nestin*, we crossed $Nes\text{-}Cre^{ER(T2)};Idh1^{fl(R132H)/+}$ and $Nes\text{-}Cre^{ER(T2)};Idh1^{+/+}$ animals with a R26-lox-STOP-lox-EYFP (R26R-EYFP) reporter mouse line. These transgenic mice have a *loxP*-flanked STOP sequence followed by the Enhanced Yellow Fluorescent Protein gene (EYFP) inserted into the *Gt(ROSA)26Sor* locus (Srinivas et al., 2001). When bred to mice expressing Cre recombinase, the STOP sequence is deleted and EYFP expression is observed in the *Cre*-expressing tissue(s) of the mutant offspring.

### Mouse genotyping

The mouse lines used in the study were genotyped from ear clipping or brain DNA using the PCR primers shown.

| Purpose | Primer ID | Forward | Reverse | Size (bp) |
|---|---|---|---|---|
| Presence of sequence between Frt sites | Flpe | ACGGAACAGCAATCAAGAGAGCCA | TCGATCCTACCCCTTGCGCTAAA | 450 |
| Presence of NeoR cassette | Neo | AGGATCTCCTGTCATCTCACC | AAGAACTCGTCAAGAAGGCGA | 493 |
| *Idh1* wildtype allele | Idh1-KI | GGTTGGACTTGGCTTTAATTTG | ATTGGTGGCATCACGATTCT | 393 |
| *Idh1* "floxed" allele | Idh1-KI | GGTTGGACTTGGCTTTAATTTG | ATTGGTGGCATCACGATTCT | 529 |
| *Idh1* Cre-recombined allele | Idh1-KI | GGTTGGACTTGGCTTTAATTTG | ATTGGTGGCATCACGATTCT | 551 |
| Cre recombinase | Cre1 | TTACCGGTCGATGCAACGAG | CCACCGTCAGTACGTGAGAT | 500 |
| Cre recombinase | Cre2 | GCGGTCTGGCAGTAAAAACTATC | GTGAAACAGCATTGCTGTCACTT | 100 |
| Internal positive control | IC | CTAGGCCACAGAATTGAAAGATCT | GTAGGTGGAAATTCTAGCATCATCC | 324 |
| Presence of YFP transgene at R26 locus | YFP | AAAGTCGCTCTGAGTTGTTAT | AAGACCGCGAAGAGTTTGTC | 320 |
| Presence of wild type allele at R26 locus | YFP | AAAGTCGCTCTGAGTTGTTAT | GGAGCGGGAGAAATGGATATG | 600 |

Mouse behavior

The open field test (OFT) was used to investigate mutant mouse behavior. A new environment causes mice to move and explore, but anxiety and fear cause mice to crouch and freeze. In general, mice that are inactive in an OFT are assumed to have anxiety and fear, and mice that are active are assumed to have less apprehension and fear. The OF used in this study was a rectangular enclosure, marked inside with 16 square areas, and with surrounding walls to prevent escape. Tam-Idh1-KI and control mice of same age were placed individually into the OF. Numbers of squares crossed and rears made were measured during a 5 minute time period. For the test we used 4 Tam-Idh1-KI mice and 4 controls mice aged between 3 to 5 months.

Post mortem examination

The following organs were analyzed in Tam-Idh1-KI and aged-matched control mice: brain, kidneys, liver, heart, lung, spleen, esophagus, lymph nodes, gall bladder, pancreas, small intestine and large intestine and blood.

Ex vivo magnetic resonance imaging

5 and 12 month-old Tam-Idh1-KI and control mice were sacrificed under terminal anesthesia with an i.p. overdose of pentobarbitone, followed by exsanguination using intracardial perfusion with a solution 0.9% of NaCl, and then a fixative solution of 4% paraformaldehyde (PFA). Heparin at a concentration of 10 IU/ml was added to the saline solution (hepsal). To allow imaging of the brains, gadolinium-DTPA (Gd-DTPA) contrast at a concentration of 27.93 mg/ml (Bracco Diagnostics) was added to both saline and PFA solutions. Briefly, after injection with 100 µL of 200 mg/ml of pentobarbitone (Pentoject, Animalcare Limited). The thorax was then opened and the heart located. The right atrium was cut from the vena cava. A 21 gauge butterfly needle was inserted into the left ventricle and 20 ml of hepsal was injected through the butterfly to wash the blood out of the mouse. Subsequently 20 ml of the 4% PFA/contrast mix was injected to fix the mouse tissues. The mouse's head was then dissected from the body, the fur and skin were removed, and the jaw dissected. The sample, comprising the skull and the brain, was then incubated in 4% PFA solution at 4C° overnight. The following day, one sample dissected from Tam-Idh1-KI and one sample dissected from control mice were placed into 20 ml tubes (in opposite orientation one to each other), embedded entirely in 1% agarose, and left to set. MRI was performed on a horizontal bore 9.4T magnet with a Varian DirectDrive™ (Agilent Technologies, Santa Clara, CA, USA). 20 ml tubes containing one Tam-Idh1-KI sample and one control sample were positioned in a quadrature birdcage coil (2.0 cm internal diameter; RAPID MR International). In brief, $T_1$-weighted 3D multi-echo multi slices (MEMS) dataset was acquired with the following parameters: TR=500ms TE=21ms, matrix size of 256 x 256 x 256 and field of view of 2.25cm x 2.25cm x 2.25 cm. Only one 3D echo dataset was shown. To quantify ventricle volumes, 3D dataset were converted into .nifti files and segmented using itk-SNAP version 3.0 (http://www.itksnap.org/) (Yushkevich et al., 2006).

SVZ microdissection and cultures

After having killed the mice using terminal anesthesia (with an i.p. overdose of pentobarbitone when working with adult mice, or with hypothermia when working with pups) followed by decapitation, brains were extracted, and sectioned in a Zivic mouse brain slicer. 500 µm brain sections were placed in Hanks balanced salt solution and the SVZ carefully microdissected under a Leica MZ12 dissecting microscope. Adult SVZ tissues were snap-frozen in liquid nitrogen and kept at -80°C for DNA, RNA or protein extractions. Primary cultures of SVZ cells were performed from brains of C57BL6 pups at P4. SVZ tissues were dissected and dissociated with accutase (Sigma) at 37ºC. SVZ cells were then cultured in Neurobasal medium (Gibco, cat#10888-022) containing B27 (Gibco, cat#17504) glutamax (Gibco 35050-038), Pen Strep (Gibco 15070-063), EGF (20 ng/mL, Sigma) and FGF-2 (20 ng/mL, R&D).

Fluorescent immunohistochemistry, confocal microscopy and image quantification

Mice were killed as for SVZ microdissection, transcardially perfused with normal saline and then 4% paraformaldehyde (PFA). Brains were extracted, post-fixed in 4% PFA, cryoprotected in 30% sucrose and frozen. 30 µm coronal brain sections were cut on a sliding microtome (Leica), and kept in cryoprotectant at -20°C. Sections were stained using standard free-floating immunohistochemistry. Images were acquired on Zeiss 510 Metahead and Leica SP8 SMD X confocal microscopes. Around 6 brain sections per mouse were used in each staining for three regions (RMS: Bregma 2.34mm to 1.78mm; SVZ: Bregma 1.70mm to -0.82mm, Hippocampus: Bregma -0.94 to -2.30mm). At least 3 z-stacks of 15-20 optical slices (1-2 µm intervals) or 8 different single optical planes per section were acquired. For each immunofluorescence, numbers of immune-positive cells were counted in z-stacks. Quantification of immune-positive cells in single optical planes were represented as specified in the figure legends. All quantifications were done by an observer blinded to experimental conditions. All the images were quantified on Volocity 6.3 (Improvision).

Antibodies

For immunofluorescence, the following primary antibodies were used: rabbit α-type IV collagen (Rockland, cat#600-401-106-0.1), rabbit α-GFP (Abcam, cat#6556), rat α-BrdU (Novus Biologicals, cat#nb500-169), rat α-CD31 (BD Pharmingen, cat#557355), goat α-Dcx (Santa Cruz Biotechnology, cat#sc8066), rabbit α-Dcx (Abcam, cat#ab18723), chicken α-GFAP (Abcam, cat#ab4674), rabbit α-Ki67 (Abcam, cat#ab16667), mouse α-NeuN (Milliphore, cat#MAB377), rabbit α-Caspase 3 (Cell Signaling, cat#9664), rabbit α-Olig2 (Millipore, cat#ab9610), mouse α-S100β (Sigma, cat#s2657), goat α-MBP (Santa Cruz Biotechnology, cat#sc13914), mouse α-nestin (Millipore, cat#mab353), rat α-PDGFRα (CD140a) (BD Biosciences, cat# 558774). Secondary antibodies conjugated to Alexa Fluor 488, 568, 594, or 647 (Invitrogen, Sigma and Jackson Laboratory) were used as appropriate. For EdU detection, the Click-iT® EdU detection kit (Invitrogen) was used and the manufacturer's protocol was adapted to Free-Floating IHC.

Non-fluorescent immunohistochemistry

Tissue samples were fixed in 10% buffered formalin or 4% paraformaldehyde (PFA) at 4°C and embedded in paraffin. Sections (5 µm) were stained with Harris hematoxylin and eosin according to standard protocols. EnVision™+ Kits (Dako) was used for immunostaining, accordingly to manufacturer's instructions. In brief, sections (5 µm) were de-waxed in xylene and rehydrated through graded alcohols to water. For antigen retrieval, sections were pressure cooked in 1x antigen retrieval solution for 10 min and allowed to cool down. Endogenous peroxidase was blocked for 5 min. Slides were then incubated with primary antibody for 1 h at room temperature. HRP labeled polymer, conjugated with the appropriate secondary antibodies, was then applied for 30 min at room temperature. Staining was completed by a 2–10 minute incubation with 3-amino-9-ethylcarbazole (AEC)+ substrate chromogen and the development of the color reaction at the antigen site was monitored microscopically. Slides were counterstained with hematoxylin, dehydrated, cleared and then mounted. Microscopic observations and images were acquired using a Nikon wide-field TE2000U Microscope. The following primary antibodies were used: rabbit α-Ki-67 (Cell Signaling, cat#12202).

Western blotting

Total protein extraction from cells was performed according to standard methods. Tissue culture medium was removed, and cells washed twice with PBS. SDS-containing tissue lysis buffer was then added, and cells were detached using a Corning cell scraper (Sigma-Aldrich), transferred to Eppendorf tubes and incubated in SDS at 95°C for 5 min. Extracts were then homogenized by ultrasound using an acoustic transducer, and centrifuged at 14,000 rpm for 10 min. The protein-containing supernatant was removed and protein concentration was established. Total protein extraction from mouse tissue was performed using urea tissue lysis buffer, made up of 7 M urea, 10% glycerol, 10 mM Tris-HCl (pH 6.8), 1% SDS, 5mM DTT, and a cOmplete Mini Protease Inhibitor Tablet (Roche). 200 µL of buffer was added to tissue which was then homogenized through a 21 gauge needle. This homogenate was then sonicated for 10 sec x3, and then mixed by rotation for 30 min at 4°C, before being clarified by centrifugation at 13000 rpm for 15 minutes at 4°C. Protein concentrations were determined using the Pierce BCA Protein Assay Kit (Thermo Scientific). Western blots were performed with the NuPAGE Gel system (Invitrogen) according to the manufacturer's protocol. In brief, denatured lysates were loaded onto a gel to run at 150V for 2 h. The gels were then transferred onto an Immobilon-P PVDF transfer membrane (Millipore) in a semi-dry tank, and blocked by incubating for 1 h at room temperature in 10% bovine serum albumin (BSA) or 10% milk. The membranes were then incubated overnight in the appropriate primary antibody in 5% BSA, or 5% milk. After washing, the membranes were incubated in the appropriate peroxidase-conjugated secondary antibody for 1 h at room temperature. After further washes, the blots were incubated in ECL reagents (GE healthcare) and bound antibodies were detected using enhanced chemiluminescence with developing chemiluminescence film (GE Healthcare) with a developer. The primary antibodies utilized were the following: rabbit α-HIF1 (Cayman Chemical Company, cat#10006421), mouse β-Actin (Santa Cruz Biotechnology, cat#sc-47778), rabbit α-IDH1 (Cell signaling technology, cat#3997), mouse α-IDH1-R132H (Millipore, cat#MABC171), rabbit α-H3K9me3 (Abcam, cat#ab8898), rabbit α-H3K79me2 (Abcam, cat#ab3594), rabbit α-H3 (Abcam, cat#ab131711), and rabbit α-H3K27me3 (Millipore, cat#07-449).

RNA extraction and RT-PCR

RNA was purified using the RNeasy microkit (Qiagen) according to the manufacturer's instructions. When required, complementary DNA was reverse transcribed *in vitro* using the High Capacity cDNA Reverse Transcription Kit (Applied Biosystems). cDNA generated from SVZ dissected cells, was pre-amplified before qRT-PCR, using The TaqMan PreAmp (Applied Biosystems) kit and following manufacturer's instructions. Absolute quantification qRT-PCR was performed on the ABI 7900HT cycler (Applied Biosystems) with β-actin serving as an endogenous control.

A list of TaqMan Gene Expression assays (Applied Biosystems) used is available on request. Each target gene's expression was evaluated using a relative quantification approach ($2^{-\Delta\Delta CT}$ method).

DNA extraction
DNA was extracted using the QiaAmp Micro kit (Qiagen) following the manufacturer's instructions.

Nucleic acid sequencing
Sequencing of gDNA and cDNA was carried out using the 2× Big Dye Terminator v3.1 reagent (Applied Biosystems). Unincorporated dye terminators were removed with the DyeEx 2.0 Spin kit (Qiagen) and the purified products were run on the ABI 3730 DNA analyzer (Applied Biosystems). To sequence gDNA, the following primers were have been used: Idh1_ex3_F TCAAGTTGAAACAAATGTGGAAA and Idh1_IN3_R GGGTGTAGATGCCCAAAGAA. To sequence cDNA, the following primers were used: Idh1_ex3_F TCAAGTTGAAACAAATGTGGAAA and Idh1_ex4_ex5_R GCAACACCACCACCTTCTTC. The short isoform of *Idh1*, missing exons 1 and 2, was identified by sequencing of cDNAs amplified using the following primers: Idh1_fw_geno_IN2_a CAGGCTAAGCACCCATGTTT and Idh1_ex4_ex5_R GCAACACCACCACCTTCTTC. To avoid DNA contamination, RNA samples were DNAse treated and the resulting cDNA amplified with PCR primers spanning exons. All PCR conditions are available upon request.

PCR amplification of alternative "leaky" *Idh1* transcript
The *Idh1* transcript lacking exons 1 and 2, was amplified from cDNA by end-point RT-PCR (amplicon size 562bp) and sequenced using the following primers: Idh1_fw_geno_IN2_a CAGGCTAAGCACCCATGTTT and Idh1_ex4_ex5_R GCAACACCACCACCTTCTTC, the former located in the canonical intron 2, and the latter spanning canonical exons 4 and 5. Further details are available on request.

ReNcell CX immortalized cell line
The ReNcell CX immortalized cell line (Millipore Cat. No. SCC007) was originally derived from 14-week human cortical brain tissue and were cultured in ReNcell NSC Maintenance Medium (Millipore Cat. No. SCM005) according to the supplier's instructions.

Neurosphere assays
For unknown reasons, possibly relating to the central roles of the IDHs in metabolism, culture of *IDH1*-mutant human tumors has proved difficult in general and our attempts to culture primary *Idh1*-mutant neurospheres from the SVZ of Tam-Idh1-KI mice were similarly unsuccessful. We therefore adopted an alternative strategy by using lentiviral vectors to express the R132H mutation in neuronal stem/progenitor cells (NPCs) from the cerebral cortex of the human fetal brain (ReNcell CX immortalized cells) or dissected from the SVZ of a wildtype BL6 mouse of P4 age. For human neurosphere assays, ReNcell CXs were transduced with $IDH1^{R132H}$, with untransduced cells as controls; GFP-expressing constructs were used to ensure transduction efficiency (estimated 60%). For mouse neurosphere assays, NSC/NPC cells were transduced with $IDH1^{R132H}$, with cells transduced with wildtype IDH1 as controls; GFP-expressing constructs were again used to ensure transduction efficiency (estimated 60%). For assessment of neurosphere formation, cells were plated at low density (10 cells/μL, 2000 cells/$cm^2$) or at high density (100 cells/μL, 20000 cells/ $cm^2$) in non-tissue culture treated 96 or 24 well plates. The number of neurospheres was counted 7-10 days after plating for cells plated at low density and 4 days for cells plated at high density. Microscopic images were acquired using a Nikon wide-field TE2000U Microscope and analyzed using ImageJ 1.47v.

Transwell cell motility assay
The motility assay was performed in polycarbonate Transwell filter chambers of 8.0-μm pore size and 6.5-mm diameter (Costar, Cambridge, MA). The upper and bottom side of each porous polycarbonate membrane were coated with laminin at 20 mg/mL overnight. Filters were then washed with PBS and plated with cells ($2.5x10^4$/well; 2 wells for each experimental condition) on the upper side of the filter. The lower chamber was filled with ReNcell NSC Maintenance Medium containing 20 ng/mL FGF-2 and 20 ng/mL EGF (basal condition) or medium containing twice as much FGF (40 ng/mL) or 5% FCS. After 24h, cells on the upper side of the filters were mechanically removed. The cells that had migrated to the lower side were fixed with 11% glutaraldehyde in PBS and stained with 0.1% crystal violet in 20% methanol. In the assay, cells expressing $IDH1^{R132H}$ were compared to untransduced controls. Microscopic images of the cells that had passed through the filters were acquired using a Nikon wide-field TE2000U microscope and counted using NIS-Element microscope imaging software 4.0.

Molecular cloning and cell transduction with lentiviral vectors

Human *IDH1* cDNA was amplified by PCR using primers which added 1X-FLAG tag to the 3' end of the sequence. This was cloned as ClaI-NheI fragment into the pCC.sin.36.MCS.PPTWpre.CMV.tTA-S2tet lentiviral transfer vector. Subsequently a recombinant PCR-based approach, using *IDH1* cDNA as a template was applied to construct the *IDH1* R132H mutant, which was cloned into the same lentiviral transfer vector as a ClaI-BmgBI fragment, by substituting the *IDH1* cassette with the *IDH1*-R132H mutant cassette. As a control, cells were transduced with a pCC.sin.36.eGFP.PPT.Wpre.CMV.tTA-s2tet, encoding eGFP and pCC.sin.36.IDH1WT.PPTWpre.CMV.tTA-S2tet, encoding the wild type sequence of human *IDH1*. PCR primers for the above are available upon request. Vector stocks were produced by transient transfection of 293T cells. Serial dilutions of freshly harvested conditioned medium were used to infect $10^5$ cells in a six-well plate, in the presence of Polybrene (8 µg/ml). The viral p24 antigen concentration was measured by an HIV-1 p24 core profile enzyme-linked immunosorbent assay ELISA assay (Lenti-X p24 rapid titer kit, Clontech) to determine the number of infective particles before transduction and to demonstrate that transduced cells did not produce viral particles after transduction.

ROS measurements

Reactive oxygen species in brain samples (Tam-Idh1-KI n=3 controls n=3, aged between 4 and 6 months) were quantified using the Mouse Reactive Oxygen Species ELISA kit (BG Bluegene), following the manufacturer's instructions.

Quantitation of genomic 5hmC and 5mC by HPLC

Purified genomic DNA (1-10 µg) was extracted from a forebrains dissected from Tam-Idh1-KI n=13 and controls n=8 (aged between 4 to 8 months) and then incubated with 200 U of RNase A/T1 Mix (#EN0551, Thermo Scientific) in 1x NEB Buffer 2 for 2 hours at 37°C. The DNA was extracted using Phenol:Chloroform:Isoamyl Alcohol 25:24:1 (#P3803, Sigma) followed by ethanol precipitation. The recovered DNA was hydrolyzed as described (Kriaucionis and Heintz, 2009). Proteins were removed by 20 min centrifugation at 14000 g using Amicon Ultra-0.5 (3 kDa) column (#UFC5003BK, EMD Millipore). The flow-through was lyophilized and re-suspended in 50 µl Buffer A (100 mM Ammonium acetate pH 6). 40 µl of the solution was injected into the Agilent 1290 Infinity Series HPLC system. The flow rate was set at 0.400 mL/min. Nucleosides were separated using Eclipse Plus C18, 2.1x150 mm, 1.8 µm column (Agilent) at 40 °C. The run was performed using 1.8% to 30% linear gradient of 40% Acetonitrile. Acquired data were analyzed using Agilent 1290 Infinity Series software. Statistical analysis was performed using ANOVA with age and sex as an additional explanatory variables.

Quantification of genomic 5mC by sequencing

Genomic DNA (at least 200 ng) was extracted from SVZ cells dissected from Tam-Idh1-KI n=3 and controls n=3 (aged between 9 to 11 months). DNA was then sonicated to an average size of 500 bp using the Covaris S220 instrument (Covaris). Oxidative bisulfite treatment of DNA was performed using the TrueMethyl24 Kit (Cambridge Epigenetix) according to the manufacturer's instructions. 10 µL of converted material were used for sequencing library preparation using the Accel-NGS Methyl-Seq DNA Library Kit (cat#DL-ILMMS-12, Swift Bioscience) according to manufacturer's instructions with some minor adjustments. Briefly, the libraries were amplified using 10 PCR cycles with barcoded primers from the Methyl-Seq Dual Indexing Kit (cat#DI-ILMMS-12, Swift Bioscience). Amplicons were selected for an average library size of 350 bp using SPRI Select Beads (cat#B23317, Beckman Coulter). Sequencing libraries were sequenced using three lanes of the HiSeq2500 Rapid Mode. After removal of low quality bases, overlapping read pairs and duplicate reads, genome sequencing coverage of CpG cytosines was a median of 2.4x. This was sufficient for global measures of methylation changes (across CpG islands, genes or other features), but did not permit analysis at the level of specific genes or regions. To determine whether observed differences were focal or evenly dispersed, we calculated the density of 5mC in genomic bins of increasing size (ranging from 200 bp to 100 Mbp) and performed unsupervised clustering.

Metabolic analysis of embryonic brains

Ion chromatography-mass spectrometry (IC-MS) was used for analysis. Metabolites were extracted using ice cold 80% aqueous methanol. Briefly, frozen mouse tissues were weighed, added to a 6-fold volume of ice-cold 80% methanol and disrupted to obtain a single cell suspension, with a tissue homogenizer (Polytron PT1200E Homogenizer, Kinematica). The extracts were then clarified by centrifugation at 14,800 rpm for 10 min at 4 °C (this step was repeated twice), and then dried in a SpeedVac and subsequently stored at -80°C. On the day of analysis the dried extracts were re-constituted in 60 µL of Milli-Q water. Organic acids and sugar phosphates where analyzed using a Thermo Scientific ICS-5000+ ion chromatography system coupled directly to a Q Exactive HF Hybrid

Quadrupole-Orbitrap mass spectrometer using a HESI II Electrospray ionization source (Thermo Scientific, San Jose, CA). The ICS-5000+ HPLC system incorporated an electrolytic anion generator (KOH) which was programmed to produce the desired hydroxide ion gradient. An inline electrolytic suppressor removed the OH- ions and cations from the post-column eluent prior to MS analysis (Thermo Scientific Dionex AERS 500). A 10 µL partial loop injection was used for all analyses and the separation was performed using a Thermo Scientific Dionex IonPac AS11-HC 2 × 250 mm, 4 µm particle size column with a Dionex Ionpac AG11-HC 4 µm 2x50 guard column inline. The IC flow rate was 0.250 mL/min. The total run time was 37 minutes and the hydroxide ion gradient comprised the following: 0 min, 0 mM; 1min, 0 mM; 15 min, 60 mM; 25 min, 100 mM; 30 min, 100 mM; 30.1min, 0 mM; 37 min, 0 mM. Analysis was performed in negative ion mode using a scan range from 80-900 and resolution set to 70,000. The tune file source parameters were set as follows: Sheath gas flow 60; Aux gas flow 20; Spray voltage 3.6; Capillary temperature 320; S-lens RF value 70; Heater temperature 450. AGC target was set to 1e6 and the Max IT value was 250 ms. The column temperature was kept at 30°C throughout the experiment. Data were acquired in the full scan, continuum mode. Peak retention times were identified from the injection of authentic standards and peaks from unknown samples were identified using a combination of accurate mass analysis (<2 ppm) and retention time using Thermo Scientific Quanbrowser software (Thermo Fisher Scientific, Hemel Hempstead, UK).

Metabolic analysis of adult brains

All adult brain samples were analyzed with IC-MS as above, and additionally using capillary electrophoresis time-of-flight mass spectrometry (CE-TOFMS) or (for α-ketoglutarate) the higher sensitivity method of liquid chromatography time-of-flight mass spectrometry (LC-TOF-MS) method. Forebrain extractions were used, because the quantity of material obtained from the SVZ was too small for optimal analysis. To prepare samples, frozen tissues were completely homogenized by a cell disrupter (Shake Master NEO; Bio Medical Science) at 4°C, after adding 500 µL of methanol containing internal standards [20 µM each of methionine sulfone and 2-(N-morpholino)ethanesulfonic acid (MES)]. The homogenate was then mixed with 200 µL of Milli-Q water and 500 µL of chloroform and centrifuged at 9100 *g* for 3 h at 4°C. Subsequently, the aqueous solution was centrifugally filtered through a 5-kDa cut-off filter (Millipore) to remove proteins. The filtrate was centrifugally concentrated and dissolved in 50 µL Milli-Q water containing reference compounds (200 µM each of 3-aminopyrrolidine and trimesate). Prior to CE-TOFMS analysis, the sample solution for anion was diluted 10 times and that for cation was diluted five times with Milli-Q water, respectively. For the α-ketoglutarate analysis, the sample solution was not diluted. The concentration of each metabolite was calculated as described previously (Soga et al., 2009). In all CE-TOFMS experiments, the Agilent CE capillary electrophoresis system (Agilent Technologies) was used, and in LC-TOFMS experiments, the Agilent G3250AA LC/MSD TOF system was used. For anionic metabolite profiling, the original Agilent stainless steel ESI needle was replaced with the Agilent G7100-60041 platinum needle (Soga et al., 2009). IC-MS and TOFMS data were found to be concordant in direction of effect. Statistical analysis was performed using ANOVA with source of the data, age and sex as additional explanatory variables.

Gene expression microarray analysis

RNA was extracted from SVZ cells of 9 Tam-Idh1-KI and 9 control mice (aged between 5 to 9 months old), and hybridized to Illumina Mouse WG6-v2 Expression BeadChip microarrays following the manufacturer's protocols. Raw data were imported into the R statistical software for further processing and analysis using BioConductor packages (Gentleman et al., 2004). Raw signal intensities were background corrected (using array-specific measures of background intensity based on negative control probes), prior to being transformed and normalized using the 'vsn' package (Huber et al., 2002). A range of quality control checks were made and all samples produced high quality data. The dataset was then filtered to only include probes detected above background levels (detection score >0.95) in at least 3 samples, resulting in a final dataset of 25580 probes. Statistical analysis to identify differential gene expression between the Tam-Idh1-KI and control groups was performed with the Linear Models for Microarray Analysis (limma) package (Smyth et al., 2005). Raw p values were corrected for multiple testing using the false discovery rate (FDR) controlling procedure of Benjamini and Hochberg (Benjamini and Hochberg, 1995). At 5% FDR, 33 probes showed significant changes in their expression level between experimental groups. Results were annotated with gene information using the relevant BioConductor annotation package (https://bioconductor.org/packages/release/data/annotation/html/illuminaHumanv4.db.html). Gene set enrichment analysis (GSEA) was performed using the 4725 gene set from the BROAD Institute with the molecular signature C2 (http://www.broadinstitute.org/gsea/msigdb/collections.jsp) using Kolmogorov-Smirnov statistics and gene shuffling permutations as described (Subramanian et al., 2005). Genes were ranked by the moderated t-statistics from the limma package. If multiple probes were present for a gene, the probe with the most significant p value for differential expression was selected. We used gene shuffling with 1,000 permutations to compute the p value for the enrichment

score. In Figure 8, illustrative plots are shown for the following gene sets: Cell cycle and DNA replication using Reactome gene sets; Stemness (Ramalho-Santos et al., 2002); Genes enriched in glioblastoma proneural (Verhaak et al., 2010); Myc target genes (Zeller et al., 2003); and Wnt pathway targets requiring Myc (Sansom et al., 2007)).

## SUPPLEMENTAL REFERENCES


Benjamini, Y., and Hochberg, Y. (1995). Controlling the false discovery rate: a practical and powerful approach to multiple testing. J. Roy. Stats. Soc. Series B *57*: 289–300.

Curtis, M. A., Kam, M., Nannmark, U., Anderson, M. F., Axell, M. Z., Wikkelso, C., HoltÂs, S., van Roon-Mom, W. M., Bjork-Eriksson, T., Nordborg, C., et al. (2007). Human neuroblasts migrate to the olfactory bulb via a lateral ventricular extension. Science *315*, 1243-1249.

Doetsch, F., Caille, I., Lim, D. A., Garcia-Verdugo, J. M., and Alvarez-Buylla, A. (1999). Subventricular zone astrocytes are neural stem cells in the adult mammalian brain. Cell *97*, 703-716.

Ernst, A., Alkass, K., Bernard, S., Salehpour, M., Perl, S., Tisdale, J., Possnert, G., Druid, H., and Frisen, J. (2014). Neurogenesis in the striatum of the adult human brain. Cell *156*, 1072-1083.

Gentleman, R. C., Carey, V. J., Bates, D. M., Bolstad, B., Dettling, M., Dudoit, S., Ellis, B., Gautier, L., Ge, Y., Gentry, J., et al. (2004). Bioconductor: open software development for computational biology and bioinformatics. Genome Biol. *5*, R80.

Huber, W., von Heydebreck, A., Sultmann, H., Poustka, A., and Vingron, M. (2002). Variance stabilization applied to microarray data calibration and to the quantification of differential expression. Bioinformatics *18 Suppl. 1*, S96-104.

Kriaucionis, S., and Heintz, N. (2009). The nuclear DNA base 5-hydroxymethylcytosine is present in Purkinje neurons and the brain. Science *324*, 929-930.

Mamber, C., Kozareva, D. A., Kamphuis, W., and Hol, E. M. (2013). Shades of gray: The delineation of marker expression within the adult rodent subventricular zone. Progress Neurobiol. *111*, 1-16.

Mirzadeh, Z., Merkle, F. T., Soriano-Navarro, M., Garcia-Verdugo, J. M., and Alvarez-Buylla, A. (2008). Neural stem cells confer unique pinwheel architecture to the ventricular surface in neurogenic regions of the adult brain. Cell Stem Cell *3*, 265-278.

Noushmehr, H., Weisenberger, D. J., Diefes, K., Phillips, H. S., Pujara, K., Berman, B. P., Pan, F., Pelloski, C. E., Sulman, E. P., Bhat, K. P., et al. (2010). Identification of a CpG island methylator phenotype that defines a distinct subgroup of glioma. Cancer Cell *17*, 510-522.

Ramalho-Santos, M., Yoon, S., Matsuzaki, Y., Mulligan, R. C., and Melton, D. A. (2002). "Stemness": transcriptional profiling of embryonic and adult stem cells. Science *298*, 597-600.

Sajad, M., Chawla, R., Zargan, J., Umar, S., Sadaqat, M., and Khan, H. A. (2011). Cytokinetics of adult rat SVZ after EAE. Brain Res. *1371*, 140-149.

Sanai, N., Nguyen, T., Ihrie, R. A., Mirzadeh, Z., Tsai, H. H., Wong, M., Gupta, N., Berger, M. S., Huang, E., Garcia-Verdugo, J. M., et al. (2011). Corridors of migrating neurons in the human brain and their decline during infancy. Nature *478*, 382-386.

Sansom, O. J., Meniel, V. S., Muncan, V., Phesse, T. J., Wilkins, J. A., Reed, K. R., Vass, J. K., Athineos, D., Clevers, H., and Clarke, A. R. (2007). *Myc* deletion rescues Apc deficiency in the small intestine. Nature *446*, 676-679.

Silva-Vargas, V., Crouch, E. E., and Doetsch, F. (2013). Adult neural stem cells and their niche: a dynamic duo during homeostasis, regeneration, and aging. Curr. Opinion Neurobiol. *23*, 935-942.

Smyth, G. K., Michaud, J., and Scott, H. S. (2005). Use of within-array replicate spots for assessing differential expression in microarray experiments. Bioinformatics *21*, 2067-2075.

Soga, T., Igarashi, K., Ito, C., Mizobuchi, K., Zimmermann, H. P., and Tomita, M. (2009). Metabolomic profiling of anionic metabolites by capillary electrophoresis mass spectrometry. Analytical Chem. *81*, 6165-6174.

Subramanian, A., Tamayo, P., Mootha, V. K., Mukherjee, S., Ebert, B. L., Gillette, M. A., Paulovich, A., Pomeroy, S. L., Golub, T. R., Lander, E. S., and Mesirov, J. P. (2005). Gene set enrichment analysis: a knowledge-based approach for interpreting genome-wide expression profiles. Proc. Natl. Acad. Sci. USA *102*, 15545-15550.

Turcan, S., Rohle, D., Goenka, A., Walsh, L. A., Fang, F., Yilmaz, E., Campos, C., Fabius, A. W., Lu, C., Ward, P. S., et al. (2012). *IDH1* mutation is sufficient to establish the glioma hypermethylator phenotype. Nature *483*, 479-483.

Yushkevich, P. A., Piven, J., Hazlett, H. C., Smith, R. G., Ho, S., Gee, J. C., and Gerig, G. (2006). User-guided 3D active contour segmentation of anatomical structures: significantly improved efficiency and reliability. NeuroImage *31*, 1116-1128.

Zeller, K. I., Jegga, A. G., Aronow, B. J., O'Donnell, K. A., and Dang, C. V. (2003). An integrated database of genes responsive to the Myc oncogenic transcription factor: identification of direct genomic targets. Genome Biol. *4*, R69.
